# Synthesis of C_7_/C_8_-cyclitols and C_7_N-aminocyclitols from maltose and X-ray crystal structure of *Streptomyces coelicolor* GlgEI V279S in a complex with an amylostatin GXG–like derivative

**DOI:** 10.3389/fchem.2022.950433

**Published:** 2022-09-09

**Authors:** Radhika Thanvi, Thilina D. Jayasinghe, Sunayana Kapil, Babatunde Samuel Obadawo, Donald R. Ronning, Steven J. Sucheck

**Affiliations:** ^1^ Department of Chemistry and Biochemistry, The University of Toledo, Toledo, OH, United States; ^2^ Department of Pharmaceutical Sciences, University of Nebraska Medical Center, Omaha, NE, United States

**Keywords:** C7/C8-cyclitols, C7N-aminocyclitols, enzyme inhibitors, *Streptomyces coelicolor* GlgEI, protein X-ray structure

## Abstract

C_7_/C_8_-cyclitols and C_7_N-aminocyclitols find applications in the pharmaceutical sector as α-glucosidase inhibitors and in the agricultural sector as fungicides and insecticides. In this study, we identified C_7_/C_8_-cyclitols and C_7_N-aminocyclitols as potential inhibitors of *Streptomyces coelicolor* (*Sco*) GlgEI-V279S based on the docking scores. The protein and the ligand (targets **11**, **12**, and **13**) were prepared, the states were generated at pH 7.0 ± 2.0, and the ligands were docked into the active sites of the receptor *via* Glide™. The synthetic route to these targets was similar to our previously reported route used to obtain 4-⍺-glucoside of valienamine (**AGV**), except the protecting group for target **12** was a *p*-bromobenzyl (PBB) ether to preserve the alkene upon deprotection. While compounds **11**–**13** did not inhibit *Sco* GlgEI-V279S at the concentrations evaluated, an X-ray crystal structure of the *Sco* GlgE1-V279S/**13** complex was solved to a resolution of 2.73 Å. This structure allowed assessment differences and commonality with our previously reported inhibitors and was useful for identifying enzyme–compound interactions that may be important for future inhibitor development. The Asp 394 nucleophile formed a bidentate hydrogen bond interaction with the exocyclic oxygen atoms (C(3)-OH and C(7)-OH) similar to the observed interactions with the *Sco* GlgEI-V279S in a complex with **AGV** (PDB:7MGY). In addition, the data suggest replacing the cyclohexyl group with more isosteric and hydrogen bond–donating groups to increase binding interactions in the + 1 binding site.

## Introduction

**FIGURE 1:**
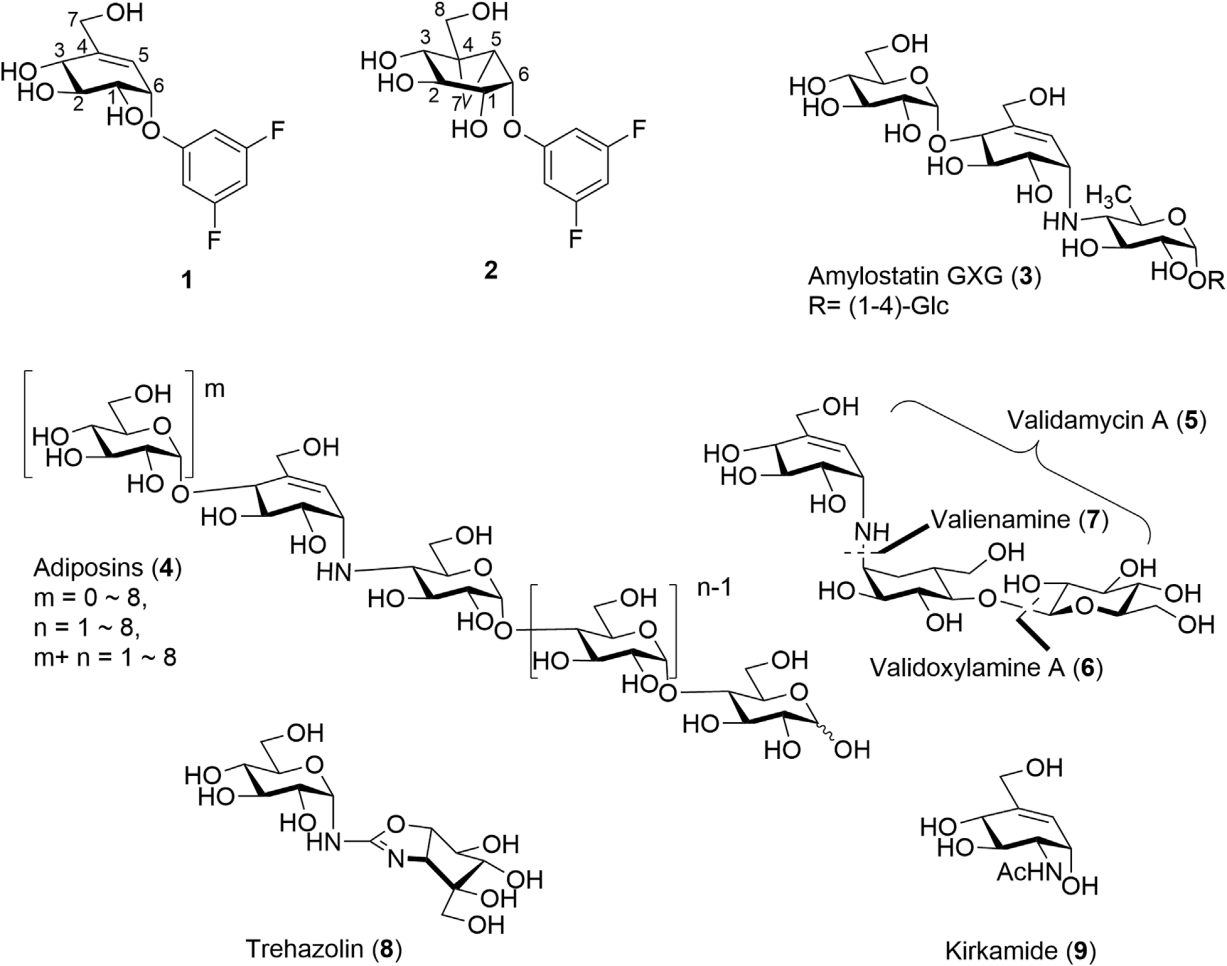
Previously isolated/synthesized C_7_/C_8_-cyclitols and C_7_N-aminocyclitols inhibitors.

Cyclitols and aminocyclitols are an important class of compounds found as both natural products or produced by chemical means. Of these, C_7_-cyclitols, some C_8_-cyclitols, and C_7_N-aminocyclitols stand out for their ability to mimic carbohydrates and interfere with a variety of enzymatic processes. For example, derivatives of valienol (streptol) (Hsiao et al., 2019) or some inositols such as C_7_-cyclitols **1** and C_8_-cyclitols **2**, Figure 1 each with good leaving groups at the C-1 position have found roles as mechanism-based inhibitors of the α-glucosidases (Chakladar et al., 2014; Adamson et al., 2016; Shamsi Kazem Abadi et al., 2017). The strained cyclopropyl in C_8_-cyclitol **2** is believed to enable the enzyme-catalyzed formation of cationic intermediates within the active sites of retaining α-galactosidases (Chakladar et al., 2014). Closely related C_7_N-aminocyclitols such as the amylostatins (**3**) (Fukuhara et al., 1982a;b;Sakairi and Kuzuhara, 1982) and adiposins (**4**) are also known inhibitors of α-glucosidases, Figure 1. On the other hand, validamycin A (**5**) has important applications in agriculture due to its insecticidal, fungicidal, and fungistatic activities. The antifungal activity of **5** is attributed to its ability to inhibit trehalase (Treh) in the fungi (Neyman et al., 2022; Ren et al., 2022). (+)-Validoxylamine-A (**6**) is a common core for validamycins A, C, D, E, and F and is also known inhibitor of Treh of various origins (Kameda et al., 1987; Ishikawa et al., 2005). Valienamine (**7**), an essential unit in many commercial glucosidase inhibitors such as **6** and acarbose, is a potent inhibitor itself. Other closely related aminosugars such as trehazolin (**8**) (Liebl et al., 2010; El Nemr and El Ashry, 2011) and kirkamide (**9**) (Sieber et al., 2020) share similar abilities to inhibit Treh, while the C6 epimer of valienamine, epi-valienamine is an inhibitor for various hexosaminidases (Scaffidi et al., 2007) and mannosidases (Ramstadius et al., 2009). The latter has potential as a therapeutic treatment for Gaucher disease (Lin et al., 2004).

**FIGURE 2:**
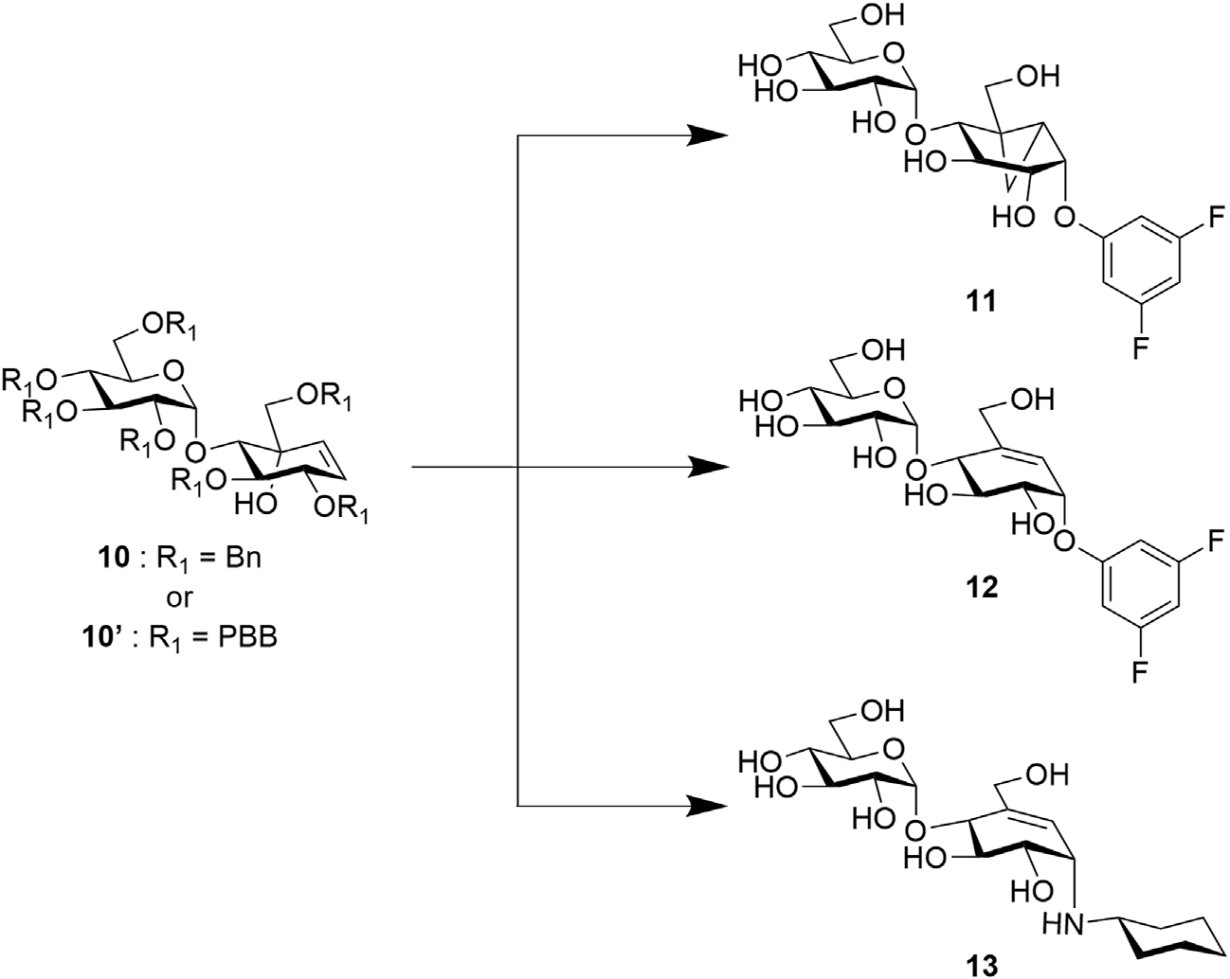
Proposed potential inhibitors **11**, **12**, and **13** from carbasugar intermediate **10**.

Although most of these covalent and non-covalent inhibitors are naturally occurring, various synthetic approaches have been adopted to chemically synthesize the C_7_/C_8_-cyclitols and C_7_N-aminocyclitols. Bennett et al. have synthesized **1** and **2**, among other covalent inhibitors, using a linear synthetic route from 2,3,4,6-tetra-*O*-benzyl glucopyranose (Chakladar et al., 2014; Adamson et al., 2016; Shamsi Kazem Abadi et al., 2017). The leaving groups were introduced toward the end using a nucleophilic aromatic substitution (Beenakker et al., 2017). Compounds **3**, **4**, **5**, and **6** and their derivatives have been synthesized by various groups (Knapp et al., 1992; Kapferer et al., 1999; Scaffidi et al., 2007) using similar approaches starting with glucose or mannose scaffolds (Ramstadius et al., 2009). Oligosaccharides such as adiposins (**4**) and acarbose (Ogawa et al., 1985; Shibata and Ogawa, 1989) have been obtained using common glycosylation methods (Mcauliffe et al., 1996). Birch reduction was widely used to deprotect the benzyl protecting groups as the conditions leave the C5–C6 double bond intact in the valienamine-derived series (Kapferer et al., 1999). Recently, our group has reported an optimized stereoselective route to obtain maltose-based carbasugar derivatives of valienamine and epi-valienamine starting from α-D-maltose (Si et al., 2021). In this work, we used a common intermediate (**10** (Si et al., 2021) or **10′**) to obtain the targets **11**, **12**, and **13** (Figure 2), which we hoped would be inhibitors of *Streptomyces coelicolor (Sco)* GlgEI V279S.

**FIGURE 3:**
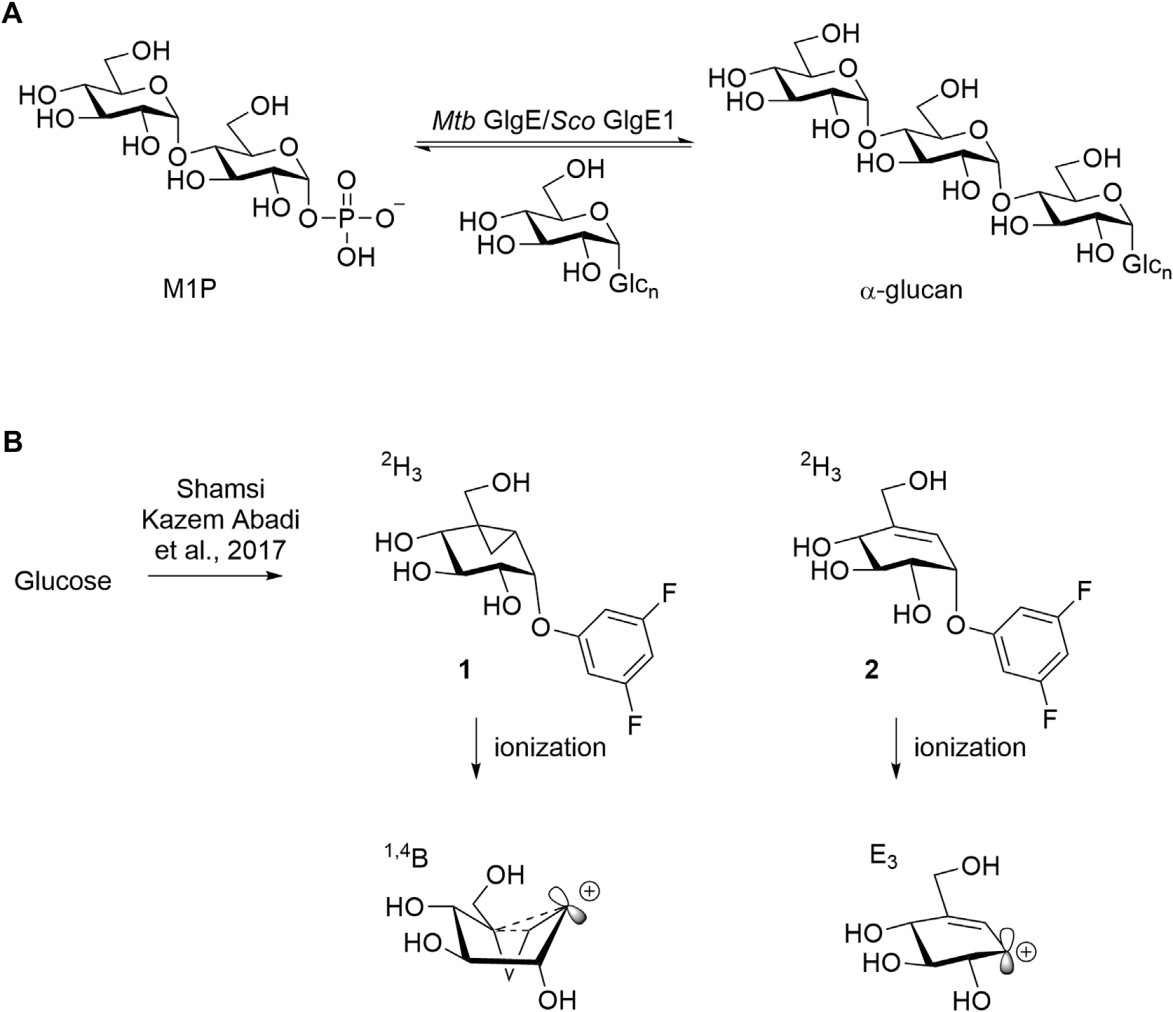
**(A)** *Mtb* GlgE *Sco* GlgEI catalyzing the conversion of M1P to α-glucan. **(B)** Structures of mechanism-based inactivators of glycoside hydrolases and proposed conformations of their ionized intermediates.

*Sco* GlgEI is a homolog of *Mycobacteria tuberculosis* (*Mtb*) GlgE. In *Mtb*, GlgE is part of a biosynthetic pathway that generates α-glucans from trehalose and is a genetically validated anti-TB target (Kalscheuer et al., 2010; Miah et al., 2013). GlgE enzymes polymerize maltose-1-phosphate (M1P) to linear α-(1→4) glucans, Figure 3A. Some of this GlgE-derived glycogen is exported in *Mtb* to form a capsule correlated with increased virulence (Sambou et al., 2008; Koliwer-Brandl et al., 2016). Mechanistically, *Mtb* GlgE and *Sco* GlgEI are similar to glycoside hydrolases (GH), but have no hydrolase activity on α-glucan, and are present in over 10% of sequenced genomes from bacteria and archaea, see Figure 3A (Chandra et al., 2011). Increasingly, new connections between bacterial glycogen and trehalose metabolism are being discovered (Chandra et al., 2011). Recently, it was found that carbon flux is diverted from cell wall synthesis in a process called the trehalose-catalytic shift which diverts trehalose into the GlgE pathway during the early stage of drug-tolerant persister bacilli formation, suggesting a vital role for the pathway in both bacilli persistence and drug resistance (Lee et al., 2019). In these studies, we used *Sco* GlgEI-V279S, which possesses the same active site topology and active site residues as *Mtb* GlgE; however, *Sco* GlgEI-V279S is more stable and is highly amenable to structural studies (Veleti et al., 2014a). For some time, our lab has been exploring the development of compounds which can be used to inhibit *Sco* GlgEI-V279S and *Mtb* GlgE (Lindenberger et al., 2015; Thanna et al., 2015; Veleti et al., 2017; Veleti et al., 2017; Si et al., 2021).

These current studies seek to identify additional reversible and covalent inhibitors which can inhibit *Sco* GlgEI-V279S. 2-Deoxy-2-fluoroglycosides have already been shown to covalently inhibit *Sco* GlgEI, and these types of deoxy glycoside inhibitors also work on several other classes of glycoside hydrolases (Vocadlo and Davies, 2008; Syson et al., 2014). Other emerging inhibitor classes include the cyclophellitol-based epoxides, aziridines, and cyclosulfates that have been shown to modify retaining α- or β-glucosidases (Gloster et al., 2007; Kallemeijn et al., 2012; Artola et al., 2017; Chen et al., 2021). Quinone methide–based glycosides also serve as activity-based inhibitors to this class of enzymes (Chauvigne-Hines et al., 2012). Our attention was drawn to the vinyl **1** and cyclopropyl **2** carbasugar inhibitors, as shown in Figure 3B, which were shown to inhibit α-retaining hydrolases (Adamson et al., 2016; Shamsi Kazem Abadi et al., 2017; Ren et al., 2018) based on our prior maltose-based carbasugar work (Si et al., 2021).

Vinyl **1** and cyclopropyl **2** carbasugars have been suggested to ionize to their resonance stabilized carbocations followed by a reaction with the active site residues. The cyclopropyl cation is noteworthy as it can adopt the bisected conformation leading to an overall ^1,4^B conformer in the carbasugar, a conformation close to the ^1^S_3_ conformation predicted to exist immediately after a nucleophilic attack on the natural substrate for GH13 class enzymes (Figure 3B). If the vinyl carbasugar were to ionize, it would be predicted to adopt an E_3_ conformation for maximal orbital overlap with the π-system, as shown in Figure 3B (Shamsi Kazem Abadi et al., 2017). The E_3_ conformation is very close to ^4^H_3_ conformation, which is generally invoked as the transition state conformation for GH13 enzymes (Shamsi Kazem Abadi et al., 2017). Thus, in this work, we sought to investigate whether maltose-based homologs of cyclopropyl and vinyl carbasugars, compounds **11** and **12**, respectively, could potentially inhibit *Sco* GlgEI-V279S. In addition, we report that the common starting material **10** can be used for entry into amylostatin GXG-like derivatives **13**, which were found to form a complex with *Sco* GlgEI-V279S.

## Results and discussion

**SCHEME 1:**
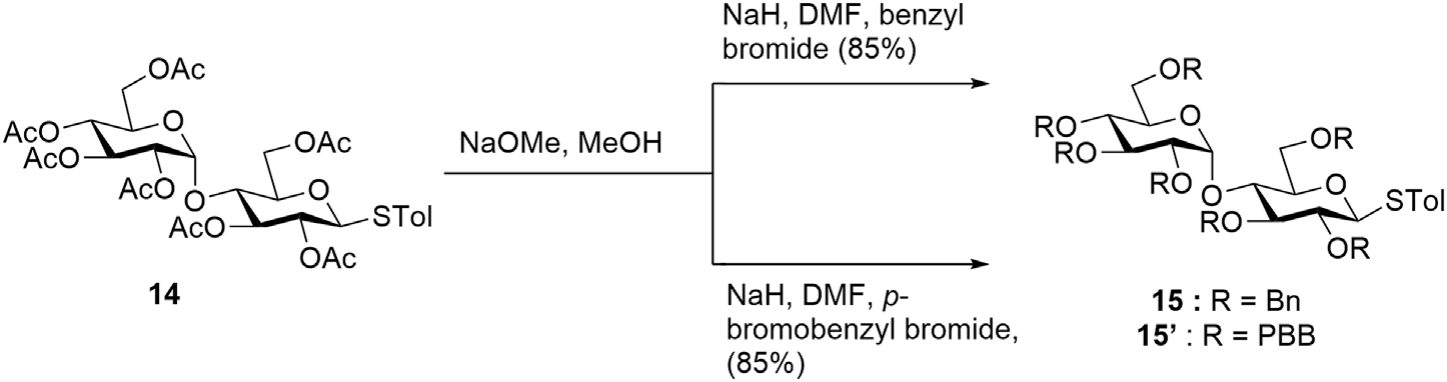
Using different protecting groups to make intermediates **15** and **15′**.

Chemistry: Targets **11** and **12** were obtained *via* a 14-step synthetic route but using two different global protecting groups. While the benzyl group was convenient to synthesize **11**, it proved to be challenging to deprotect benzyl using traditional methods without affecting the alkene group in **12**. Therefore, we used a modifiable global protecting group *p*-bromobenzyl (PBB) which could later be converted into a labile leaving group *via* a Buchwald–Hartwig amination (Plante et al., 2000). We started the synthesis using commercially available D-(+)-maltose on a gram scale. Compound **14** was obtained using reported literature in two steps (Veleti et al., 2014b). After acetyl deprotection of **14** using Zemplén conditions, we installed benzyl and *p*-bromobenzyl protecting group to access previously reported thiomaltoside **15** (Si et al., 2021) and also the new PBB-protected **15′** compound, respectively (Scheme 1).

**SCHEME 2:**
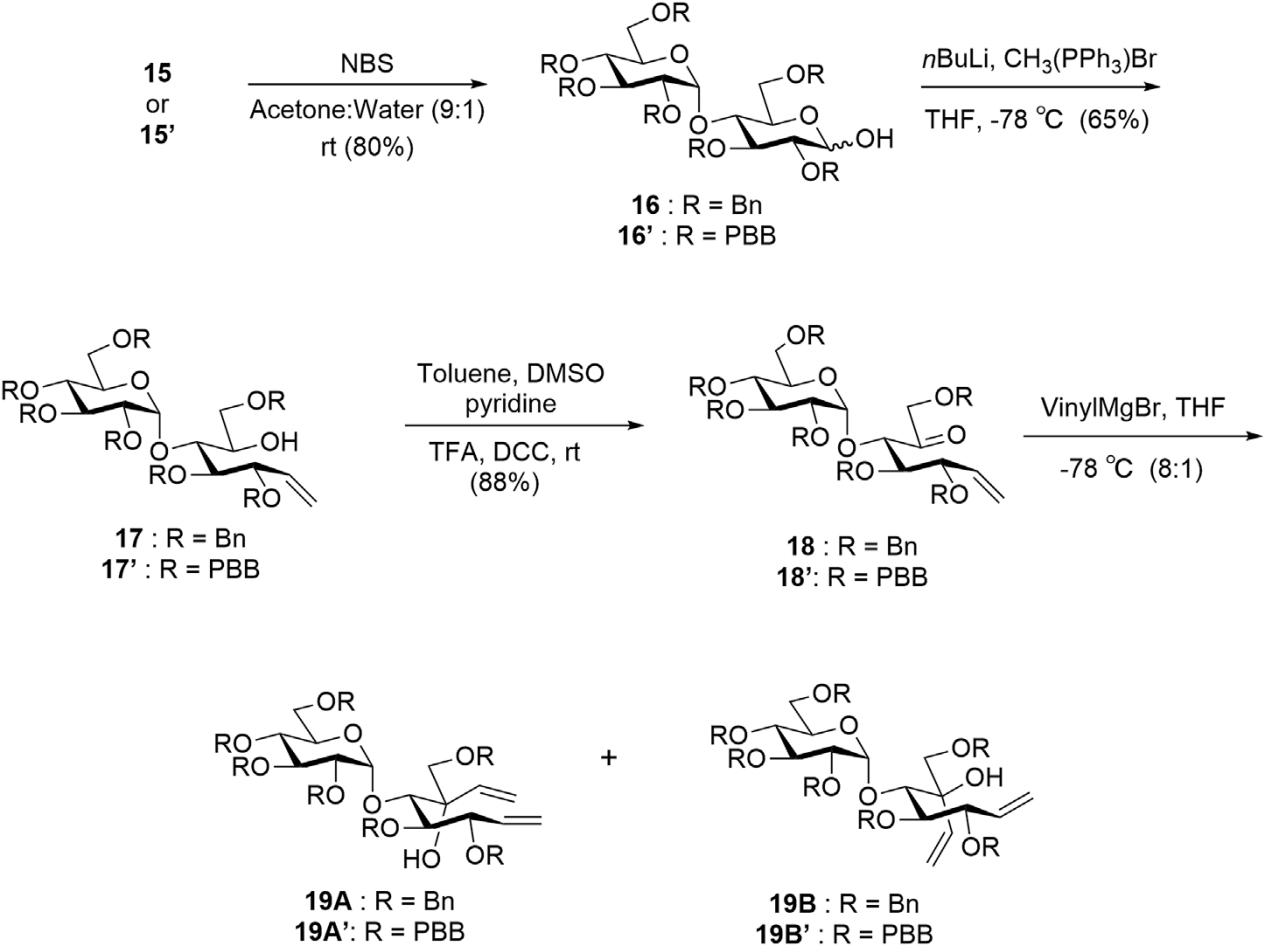
Synthesis of intermediates **19A**, **19B**, **19A′**, and **19B′**. Yields are reported for new compounds **15′** to **19A′**/**19B′**. Compounds **15**–**19A**/**19B** are previously reported and have similar yields (Si et al., 2021).

The protected thiomaltosides **15** and **15′** were each subjected to *N*-bromosuccinimide in 9:1 acetone:water to obtain hemiacetals **16** and **16′**. Compounds **16** and **16′** were subjected to Wittig olefination, Moffatt oxidation, and Grignard reactions to obtain the dienes **19A**, **19B**, **19A′**, and **19B′** as intermediates (Scheme 2) (Pfitzner and Moffatt, 1963; Chiara et al., 2008). After obtaining dienes **19A′** and **19B′**, we could conclude that **19A′** was the desired diene owing to the similarities in the NMR splitting pattern of the alkene protons with previously reported **19A** in comparison to **19A**′ and previously reported **19B** in comparison to **19B′** (Si et al., 2021). The upfield shift observed for **19A**′ (H-1′ = 5.26 ppm) relative to **19B′** (H-1′ = 5.41 ppm) was similar to the upfield shift of **19A** (H-1′ = 5.30 ppm) relative to **19B** (H-1′ = 5.44 ppm), which is indicative of the desired isomer.

**SCHEME 3:**
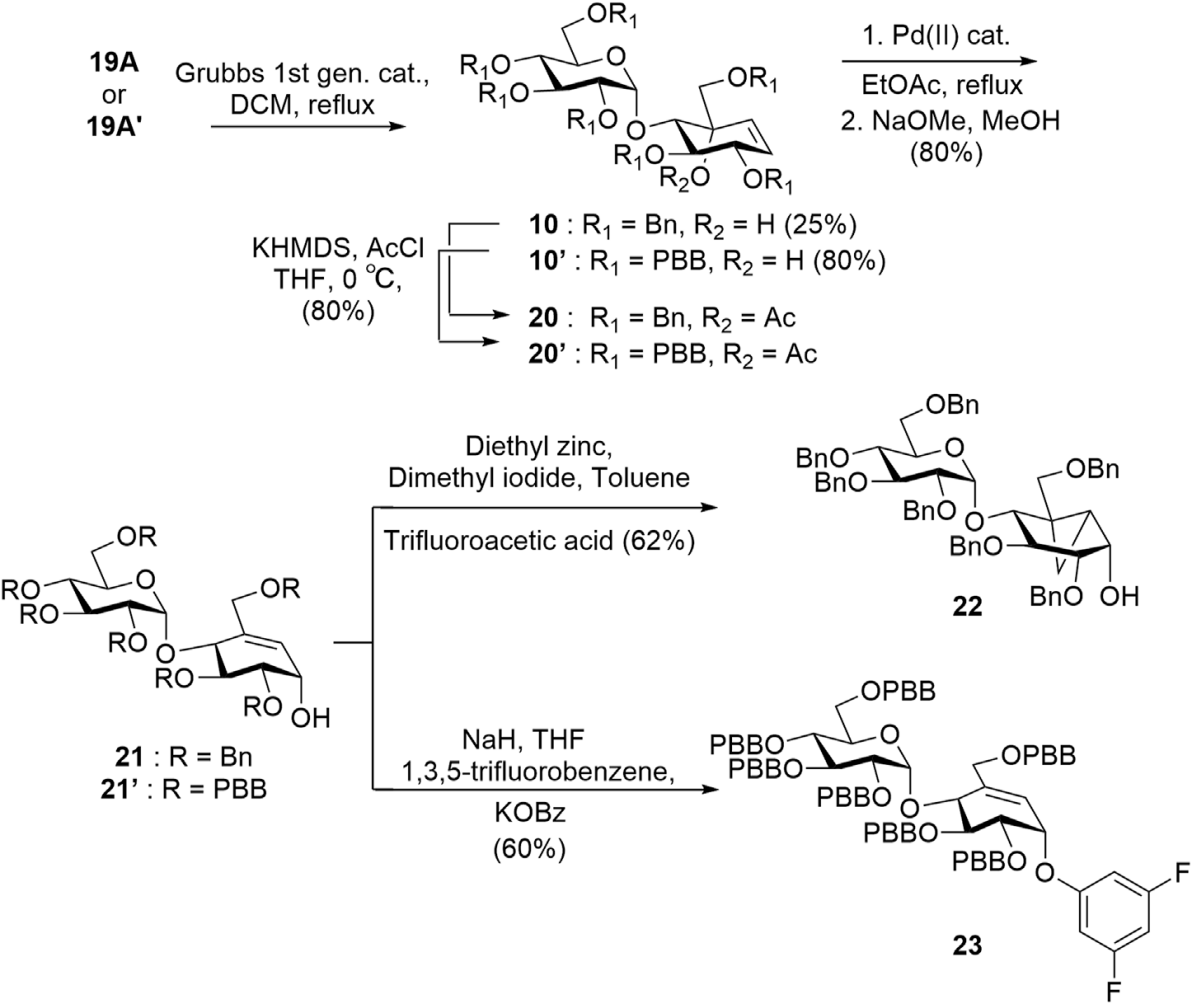
Grubbs ring-closing metathesis reaction to obtain alkenes **20** and **20′** and synthesis of key intermediates **22** and **23**.

Intermediate **19A′** was subjected to a ring-closing metathesis to afford **10A′** in 80% yield, which was notably higher than the same reaction reported for the benzyl-protected analogue **19A** which proceed in 25% yield (Scheme 3) (Si et al., 2021). Although the intermediates **19A** and **19A′** both experience similar steric bulk from their protecting groups, the presence of an electron-withdrawing group in **19A′** appears to affect the yield significantly. After obtaining the desired pseudosugars **10** and **10′**, we protected the tertiary alcohols with an acetyl group affording **20** and **20′** to set up a [1,3]-sigmatropic shift catalyzed by palladium (II) under reflux conditions to afford **21** and **21′** after Zemplén deacetylation (Lim et al., 2009; Shamsi Kazem Abadi et al., 2017). Compound **21** was subjected to Simmons–Smith conditions (Davies et al., 2007) to convert the C5–C6 alkene to the cyclopropyl adduct **22**. Compound **21′** was treated with 1,3,5-trifluorobenzene under nucleophilic aromatic substitution conditions to afford compound **23** (Scheme 3) (Chakladar et al., 2014).

**SCHEME 4:**
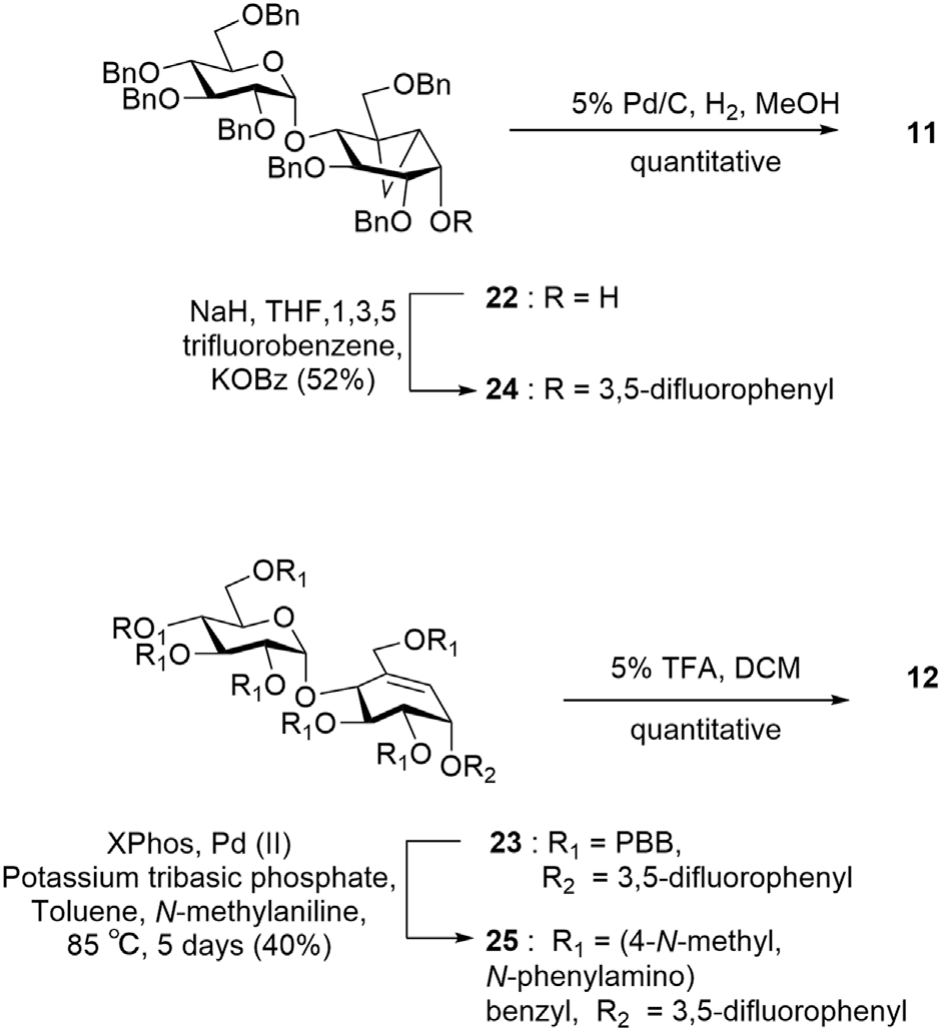
Synthesis of targets **11** and **12**.

Target **11** was obtained from intermediate **22** in two steps involving nucleophilic substitution using 1,3,5-trifluorobenzene and, ultimately, global deprotection using hydrogenolysis (Scheme 4). To obtain target **12**, we converted the *p*-bromobenzyl groups of compound **23** into labile leaving groups *via* Buchwald–Hartwig amination using *N*-methylaniline and, finally, deprotection in a weakly acidic medium as per the reported literature (Scheme 4) (Plante et al., 2000).

**SCHEME 5:**
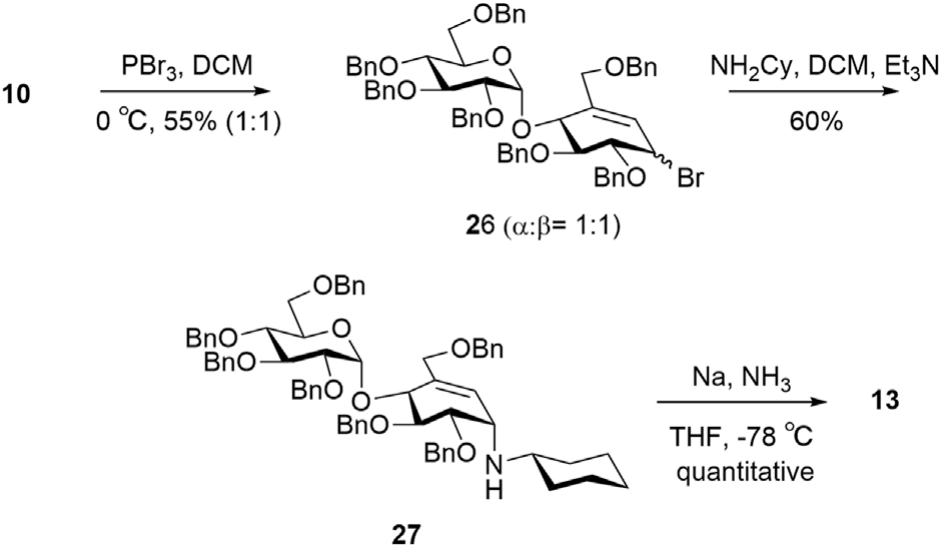
Synthesis of target **13**.

For target **13**, intermediate **10** was converted to an intermediate allylic bromide **26** (α:β, 1:1) formed using phosphorous tribromide in 55% yield (Cumpstey et al., 2011). Compound **27** was obtained by subjecting the mixture of allylic bromide **26** to an excess of cyclohexylamine and triethylamine. The final compound **13** was quantitatively obtained by Birch reduction (Scheme 5) (Kapferer et al., 1999; Si et al., 2021).

**FIGURE 4:**
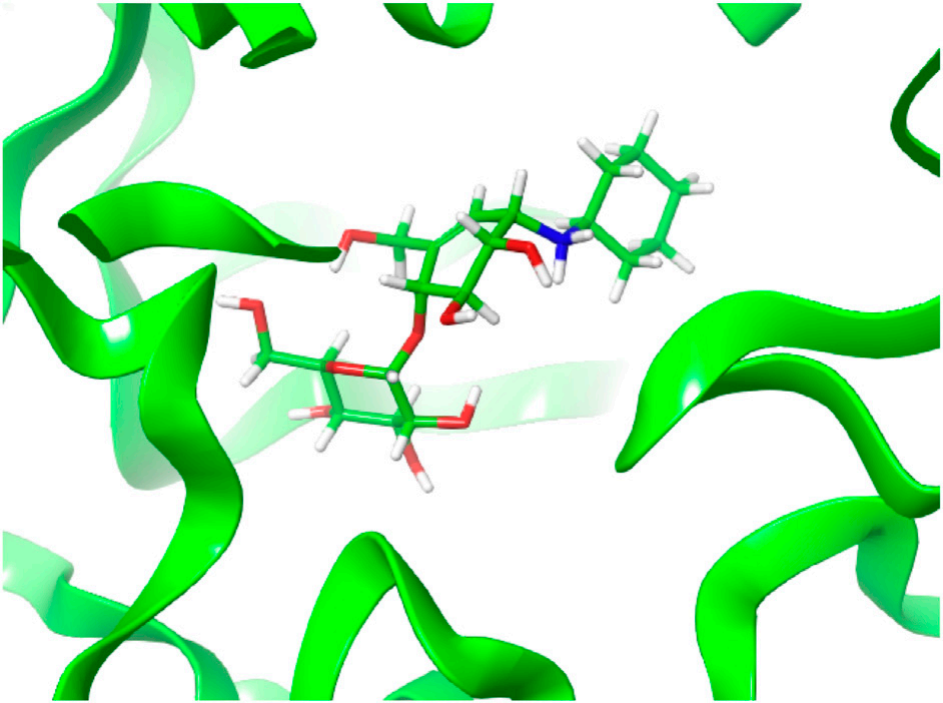
Compound **13** docked in the Sco GlgEI-V279S M1P binding site.

Computational studies: Since compounds **11–13** did not inhibit the enzyme at the concentrations tested, the *Sco* GlgEI-V279S/**13** complex crystal structure was used as a basis for additional computational docking studies. The protein and the ligands **11–13** were prepared, states generated at pH 7.0 ± 2.0, and the ligands docked into the active sites of the receptor *via* Glide™ module of the Schrödinger suite (Supplementary Figures S1, S2) (Schrödinger, L.L.C. (2017); Kumar et al., 2020; Oyeneyin et al., 2021). The docked *Sco* GlgEI-V279S/**13** complex is shown in Figure 4.

Among the three compounds, the docking scores were similar, where the hexylamine derivative recorded the highest docking score (−12.363 kcal/mol) and XP GScore (−12.377 kcal/mol) (Supplementary Table S1).

**FIGURE 5:**
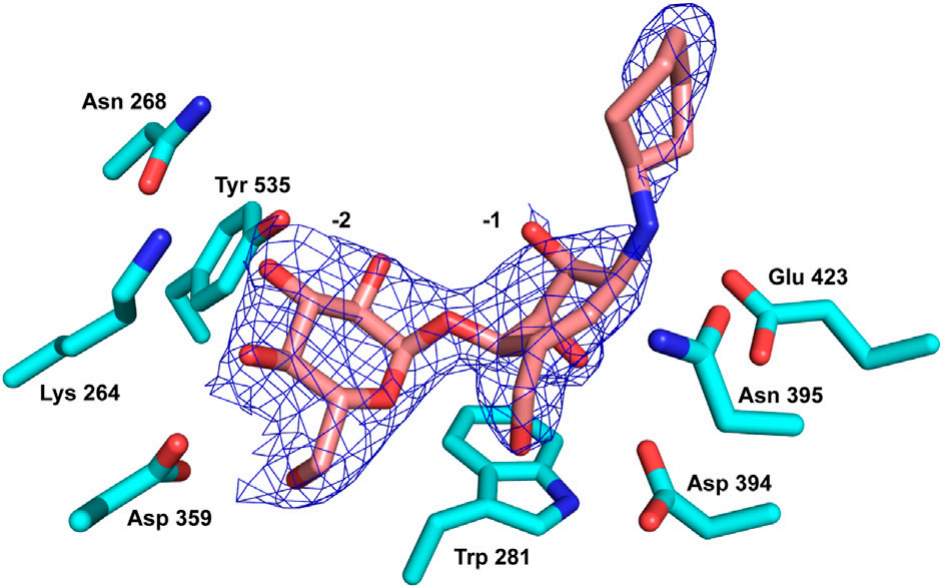
Compound **13** (pink carbon atoms) bound within the active site of *Sco* GlgEI-V279S (cyan carbon atoms). The 2Fo-Fc map is contoured at 1.5σ.

As noted, compound **13** lacks any apparent inhibition of *Sco* GlgEI-V279S at concentrations as high as 1 mM. To assess any difference or commonality with our previously tested inhibitors and identify any additional enzyme–compound interactions that may be important for future drug development, we analyzed the X-ray crystal structure of the *Sco* GlgEI-V279S/**13** complex. In particular, it is important to assess the conformation of the carbocycle in the −1 site as the computational docking model of compound **13** differs from the carbocycle conformation in the previously published *Sco* GlgEI-V279S complex with a 4-α-glucoside of valienamine (**AGV**). The *Sco* GlgEI-V279S/**13** complex structure was resolved to a resolution of 2.73 Å, and the 2Fo-Fc composite omit map illustrated the presence of compound **13** in the M1P binding site of *Sco* GlgEI-V279S (Figure 5). The difference density maps show strong density for the glucose moiety in the -2 subsite and the carbocycle in the −1 subsite. However, the cyclohexyl moiety of compound **13** exhibits only weak difference density suggesting a lack of sufficient binding interactions with the residues forming the *Sco* GlgEI-V279S +1 site. This is consistent with computational modeling.

**FIGURE 6:**
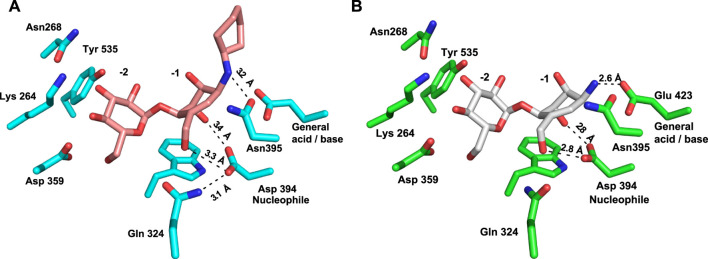
Comparison of interactions in the −1 sites of **(A)** *Sco* GlgEI-V279S/**13** (pink carbon atoms) and **(B)** *Sco* GlgEI-V279S/**AGV** (gray carbon atoms) structures.

Inspection of the enzyme–compound interactions shows many similarities to previously determine *Sco* GlgEI structures. Specifically, the glucose moiety of compound **13** bound in the −2 subsite resembles the interactions observed in previously published *Sco* GlgEI-V279S/inhibitor complex structures (Syson et al., 2011; Lindenberger et al., 2015) (Figure 6A).

Inspection of the −1 subsite illustrates that the carbocycle C(3)-OH and C(7)-OH functional groups are in an axial conformational and form a bidentate hydrogen-bonded interaction with the side chain oxygen atoms of the Asp 394 nucleophile (Figure 6A). A similar bidentate interaction was observed with the *Sco* GlgEI-V279S in a complex with **AGV** (Figure 6B) (PDB:7MGY) (Si et al., 2021). As **AGV** was used as the basis for designing compound **13**, this consistency gives confidence to the molecular design but contrasts with the computational modeling. The computational model shows these hydroxyls taking an equatorial orientation, which is conformationally lower in energy but apparently lacks appropriate interactions with the *Sco* GlgEI −1 subsite to support binding. Further comparison of these complexes demonstrates that the polar interactions within the −1 subsite of the *Sco* GlgEI-V279S/**AGV** complex are shorter and likely stronger than those of the *Sco* GlgEI-V279S/**13** complex. Specifically, the side chain of Asp 394 in the *Sco* GlgEI-V279S/**AGV** complex is well oriented toward the hydroxyl groups of C2 and C7, thereby facilitating the formation of shorter hydrogen-bonded interactions (Figure 6B). But in the *Sco* GlgEI-V279S/**13** complex, the Asp 394 side chain has slightly rotated away from the hydroxyl groups of C2 and C7 (Figure 6A).

Since both *Sco* GlgEI-V279S/**AGV** and *Sco* GlgEi-V279S/**13** crystals were formed at pH 7.5 and due to the proximity of the secondary amine of compound **13** with the side chain of Glu 423, it is hypothesized that the nitrogen atom of compound **13** is protonated and harbors a positive charge as anticipated for the primary ammonium moiety in **AGV**. Therefore, the 3.2 Å distant interaction between Glu 423 and the amine of **13** should be considered an ionic interaction. In both cases this is meant to emulate the positively charged oxocarbenium intermediate formed during the GlgE reaction mechanism. Furthermore, Figure 6A illustrates that the Glu 423 (general acid/base) in the *Sco* GlgEI-V279S/**13** complex lacks interactions with compound **13**. That is, due to the secondary amine of compound **13** shifting away from Glu 423, thereby forming a longer ionic interaction between the side chain of Glu 423 and the amine linker. In addition, this movement facilitates the formation of a hydrogen-bonded interaction between the amine and the side chain of the Asn 395 (Figure 6A).

**FIGURE 7:**
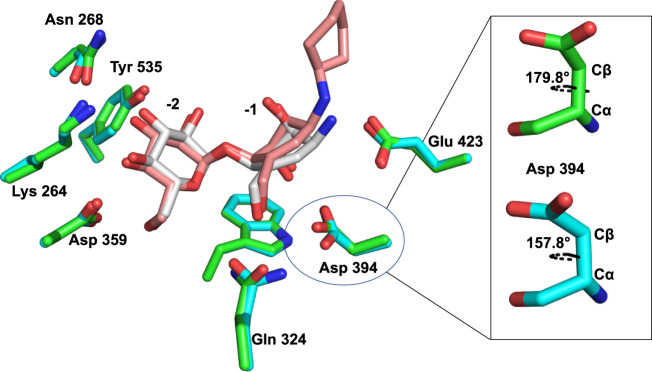
Superimposed active sites of *Sco* GlgEI-V279S/**13** (pink carbon atoms) and *Sco* GlgEI-V279S/**AGV** (gray carbon atoms) structures.

Furthermore, the oxocarbenium ion formed during catalysis is expected to take a flat chair conformation in each transition state. Figure 6A illustrates that the carbocycle of compound **13** successfully resembles the flat chair conformation. The superimposed active sites of *Sco* GlgEI-V279S/**13** and *Sco* GlgEI-V279S/**AGV** complexes further illustrate that the carbocycles of compounds **13** and **AVG** have the same conformation and the introduction of cyclohexane moiety does not affect the conformation of **13**. Superposition of the *Sco* GlgEI-V279S/**13** and *Sco* GlgEI-V279S/**AGV** complexes further highlights the shift of the amine linker in compound **13** away from the Glu 423 side chain, compared to the ammonium ion of the AGV in the *Sco* GlgEI-V279S/**AVG** complex (Figure 7).

The superimposed structures of *Sco* GlgEI-V279S/**13** and *Sco* GlgEI-V279S/**AGV** complexes further highlight a 22° χ1 dihedral angle (between Cα and Cβ atoms) difference between Asp 394 in the *Sco* GlgEI-V279S/**13** and *Sco* GlgEI-V279S/**AGV** complexes (Figure 7). The rotated Asp 394 in *Sco* GlgEI-V279S/**13** complex is further stabilized by forming a hydrogen-bonded interaction with the side chain NH of Gln 324 (Figure 6A).

Finally, the modeling of the cyclohexyl moiety of **13** in the X-ray crystal structure indicates the possible formation of only three hydrophobic interactions with the side chains of residues Asp 480, Ile 481, and Phe 425. These interactions, in contrast to the docking results, suggest a little role in supporting compound **13** binding. Alternatively, the weak difference density in Figure 5 may suggest undesired rotation of the sigma bonds flanking the amine, which could afford relatively free rotation of this moiety within the enzyme active site, which would weaken binding affinity.

## Conclusion

Based on literature (Si et al., 2021), a 750 μM concentration of **AGV** reduced *Sco* GlgEI-V279S activity by 55%. To attempt to improve on the modest inhibitory activity, we thought to couple a cyclohexane moiety to the amine moiety to examine the effect of the 3rd ring on interacting with the amino acid residues in the +1 binding site. To achieve that goal, we discovered that allylic bromide **26** could be coupled to amine nucleophiles to extend the pseudosaccharide core. This may represent a useful strategy for entry into other naturally occurring C_7_N-aminocyclitols such as the amylostatins. As expected, the *Sco* GlgEI-V279S/**13** complex structure illustrates that the carbocycle of compound **13** resembles a flat chair confirmation and maintains the axial positions for the hydroxyls at positions 2 and 3. Unfortunately, the compound **13** carbocycle interactions with the Glu 423 (general acid/base) were weakened by addition of the cyclohexyl moiety due to steric hindrance. This also promotes bond rotation in the Asp 394 side chain, which weakens an otherwise strong bidentate hydrogen-bonded interaction between Asp 394 and hydroxyls 2 and 7 of compound **13**. Unfortunately, these minor disruptions in binding lack compensating interactions between the +1 subsite and the cyclohexyl moiety of **13**, which appears to form only transitory interactions with side chains of the +1 subside. However, it is still possible to use compound **13** as the lead molecule in future GlgE inhibitor design studies. Taken together, the structure of the *Sco* GlgEI-V279S/**13** complex strongly supports changing the stereo centers at carbocycle positions 2 and 3 to allow for equatorial hydroxyl groups at these positions. However, this would eliminate one of the hydrogen bonds to Asp 394 and lower conformational energy of the molecule and the addition of three other hydrogen bonds with Arg 392 and Asp 480 as seen in GlgE structures with bound maltose or maltose analogs (Veleti et al., 2014a; Lindenberger et al., 2015; Thanna et al., 2015; Veleti et al., 2017; Veleti et al., 2017; Si et al., 2021). In addition, replacing the cyclohexyl moiety with isosteric groups possessing hydrogen bond donors may support binding specificity as well as stabilizing hydrophobic interactions with the side chains of nearby residues.

## Experimental methods

### General methods

All chemicals and solvents were purchased from Fisher Scientific, Acros Organics, Alfa Aesar, or Sigma-Aldrich. Solvents were dried by using a solvent purification system by passing through activated alumina and copper catalyst columns. All reactions were carried out at room temperature under a nitrogen atmosphere using a nitrogen balloon, unless mentioned otherwise. Reactions were monitored by TLC (silica gel, f_254_) under UV light or by charring (5% H_2_SO_4_-MeOH), and the purification was performed by column chromatography on a silica gel (230–400 mesh) using the solvent system specified. Solvents were used without purification for chromatography. ^1^H NMR was recorded on the Bruker Avance III 600 MHz spectrometer using CDCl_3_ and D_2_O as solvents with residual CDCl_3_ and D_2_O as references. ^13^C were recorded on the Bruker Avance III 600 MHz spectrometer using CHCl_3_ and HDO as internal references. High resolution mass spectrometry was carried out using the TOF MS-ES + instrument. Low resolution mass spectrometry was carried out on ESquire-LC-MS.

#### 4-Methylthiophenyl-2,3,6-tri-*O*-(4-bromobenzyl)-4-*O*-(2′,3′,4′,6′-tetra-*O*-(4-bromobenzyl)-α-D-glucopyranosyl)-β-D-glucopyranoside (15′)

A solution of deacetylated **14** (2.95 g, 9.04 mmol) in dry *N,N-*dimethylformamide (50 ml) was cooled to 0°C. The solution was treated dropwise with a suspension of sodium hydride (60% dispersion in mineral oil) (4.55 g, 114.0 mmol) in dry *N,N-*dimethylformamide. *p*-Bromobenzyl bromide (23.8 g, 95.3 mmol) was added dropwise over 15 min, and the solution was stirred at room temperature for 16 h. The reaction was poured over ice and extracted with ethyl acetate (50 ml). The combined organic layers were washed with brine. The resulting organic phase was dried (anhydrous Na_2_SO_4_) and filtered, and the filtrate was concentrated under reduced pressure to obtain a product. The product was purified by silica gel flash column chromatography. The product fractions were combined, concentrated, and dried in vacuum to afford a yellow oily product **15′**: yield 85% (2.50 g); silica gel TLC *R*_*f*_ = 0.61 (25% ethyl acetate:hexane). ^1^H NMR (600 MHz, CDCl_3_) δ 7.52–7.35 (m, 17H, Ar), 7.33–6.89 (m, 15H, Ar), 5.51 (d, *J =* 3.6 Hz, 1H, H-1′), 4.80 (d, *J =* 10.6 Hz, 1H, OCH_2_Ph), 4.76–4.66 (m, 5H, OCH_2_Ph), 4.63 (dd, *J =* 11.3, 6.3 Hz, 2H, OCH_2_Ph), 4.58 (d, *J =* 9.7 Hz, 1H, H-1), 4.52–4.42 (m, 6H, OCH_2_Ph), 4.35–4.29 (m, 2H, OCH_2_Ph), 4.23 (d, *J =* 12.4 Hz, 1H, OCH_2_Ph), 4.00 (t, *J =* 9.3 Hz, 1H, H-6a′), 3.84 (dd, *J =* 11.3, 3.9 Hz, 1H, H-5), 3.81–3.74 (m, 2H, H-3, H-3′), 3.70 (t, *J =* 8.9 Hz, 1H, H-5′), 3.55 (d, *J =* 9.8 Hz, 1H, H-4), 3.51 (ddd, *J =* 9.7, 3.8, 2.1 Hz, 1H, H-4′), 3.48 (d, *J =* 9.5 Hz, 1H, H-6b′), 3.46–3.42 (m, 1H, H-6a), 3.39 (dd, *J =* 9.9, 3.7 Hz, 1H, H-2), 3.32 (dd, *J =* 10.5, 1.8 Hz, 1H, H-2′), 2.93 (d, *J =* 44.1 Hz, 1H, H-6b), and 2.35 (s, 3H, -CH_3_). ^13^C NMR (151 MHz, CDCl_3_) δ 136.93 (Ar), 136.61 (Ar), 132.65 (Ar), 131.64 (Ar), 131.56 (Ar), 131.54 (Ar), 131.52 (Ar), 131.51 (Ar), 131.46 (Ar), 131.43 (Ar), 130.73 (Ar), 129.79 (Ar), 129.73 (Ar), 129.65 (Ar), 129.20 (Ar), 129.18 (Ar), 128.98 (Ar), 128.60 (Ar), 127.65 (Ar), 122.12 (Ar), 121.83 (Ar), 120.72 (Ar), 120.68 (Ar), 120.52 (Ar), 97.34 (C1′), 87.67 (C1), 86.95 (C5′), 81.91 (C4′), 81.11 (C4), 79.59 (C2), 78.91 (C2′), 77.70 (C5′), 76.82 (C3′), 74.74 (C3), 74.38 (OCH_2_Ph), 72.90 (OCH_2_Ph), 71.12 (OCH_2_Ph), 69.30 (C6′), 68.36 (C6), 64.85 (OCH_2_Ph), and 21.20 (-CH_3_). Mass spectrum (HRMS), *m/z* = 1654.8255 (M+Na)^+^; C_68_H_63_Br_7_O_10_S requires 1654.8335 (M+Na)^+^.

#### 2,3,6-Tri-*O*-(4-bromobenzyl)-4-*O*-(2′,3′,4′,6′-tetra*-O-*(4-bromobenzyl)-α-D-glucopyranosyl)-α/β-D-glucopyranoside (16′)

*N*-bromosuccinimide (0.807 g, 4.56 mmol) was added to a solution of **15′** (2.47 g, 1.52 mmol) in 9:1 acetone:water (70 ml) and stirred at room temperature for 45 min. The solvent was evaporated at room temperature until turbid. A solution of the residue in ethyl acetate (100 ml) was washed successively with satd. aq. NaHCO_3_, (3 X 50 ml) and water (3 X 50 ml). The solution was dried with anhydrous Na_2_SO_4_ and evaporated. The product was purified by silica gel flash column chromatography. The product fractions were combined, concentrated, and dried in vacuum to afford as yellow oil **16′**: yield 80% (2.00 g); silica gel TLC *R*_*f*_ = 0.19 (30% ethyl acetate:hexane). ^1^H NMR (600 MHz, CDCl_3_) δ 7.50—7.33 (m, 26 H, Ar), 7.17—6.90 (m, 30H, Ar), 5.54 (dd, *J =* 14.1, 3.6 Hz, 2H), 5.27 (d, *J =* 3.3 Hz, 1H), 4.89–4.82 (m, 2H), 4.81 (d, *J =* 12.2 Hz, 1H), 4.78–4.73 (m, 4H), 4.68 (dd, *J =* 19.6, 11.0 Hz, 4H), 4.63 (d, *J =* 12.2 Hz, 1H), 4.55 (dt, *J =* 11.9, 10.3 Hz, 4H), 4.47 (dd, *J =* 22.4, 11.0 Hz, 8H), 4.42 (s, 1H), 4.35 (dd, *J =* 11.2, 2.4 Hz, 2H), 4.30–4.23 (m, 3H), 4.18–4.11 (m, 4H), 4.05 (t, *J =* 9.0 Hz, 1H), 3.98 (s, 1H), 3.96 (dd, *J =* 18.0, 8.8 Hz, 1H), 3.81 (ddd, *J =* 19.0, 17.0, 9.4 Hz, 2H), 3.70 (ddd, *J =* 27.0, 11.7, 3.2 Hz, 4H), 3.60–3.52 (m, 5H), 3.43 (ddd, *J =* 14.3, 9.3, 4.0 Hz, 3H), and 3.38–3.32 (m, 2H). ^13^C NMR (151 MHz, CDCl_3_) δ 137.69, 137.49, 137.39, 137.35, 136.98, 136.94, 136.89, 136.72, 136.66, 136.64, 136.62, 136.42, 131.71, 131.68, 131.67, 131.66, 131.62, 131.61, 131.59, 131.57, 131.56, 131.53, 131.51, 131.49, 131.49, 131.47, 131.46, 131.42, 131.41, 131.40, 129.69, 129.68, 129.67, 129.65, 129.62, 129.60, 129.36, 129.33, 129.32, 129.31, 129.30, 129.28, 129.15, 129.14, 129.13, 129.09, 129.07, 129.06, 129.05, 129.05, 129.04, 129.04, 129.03, 129.02, 129.01, 127.92, 127.80, 122.06, 121.85, 121.83, 121.77, 121.75, 121.71, 121.63, 121.55, 121.54, 121.50, 121.07, 121.06, 97.41, 96.94, 96.84, 90.56, 84.38, 82.82, 81.72, 81.32, 80.16, 79.45, 79.28, 77.56, 77.34, 77.13, 76.92, 74.58, 74.24, 74.20, 73.68, 73.43, 73.21, 73.13, 73.07, 72.75, 72.74, 72.69, 72.58, 72.22, 71.06, 70.96, 69.70, 69.34, 69.22, 68.06, and 60.50. Mass spectrum (HRMS), *m/z* = 1548.8017 (M+Na)^+^; C_61_H_57_Br_7_O_11_ requires 1548.8057 (M+Na)^+^.

#### 3,4,7-Tri-*O*-(4-bromobenyl)-5-*O*-(2′,3′,4′,6′-tetra-*O*-(4-bromobenzyl)-α-D-glucopyranosyl)-D-gluchept-1-enitol (17′)

*n*-Butyl lithium (2.22 M) in hexanes (2.25 ml, 5.00 mmol) was added dropwise to a suspension of methyltriphenylphosphonium bromide (1.78 g, 5.00 mmol) in tetrahydrofuran (50 ml) at −20°C. The solution was stirred at −20°C for 15 min and raised to ambient temperature over 1 h. The solution was cooled to −20°C and compound **16′** (1.9 g, 1.25 mmol) dissolved in 50 ml of tetrahydrofuran was added dropwise. The solution was stirred at −20°C for 15 min and allowed to warm to ambient temperature and stirred for an additional 12 h. The solution was diluted with acetone (6 ml) and stirred for 30 min. Diethyl ether (40 ml) was added to precipitate triphenylphosphine oxide. The latter was removed by filtration using a Celite™ 545 filter aid. The filtrate was washed successively with saturated aq. NaHCO_3_ and brine (50 ml X 3). The solution was dried with anhydrous Na_2_SO_4_ and filtered, and the filtrate concentrated under reduced pressure to obtain the crude product. The product was purified by silica gel flash column chromatography on a silica gel to give product as a yellow oil **17′**: yield 65% (1.30 g); *R*_*f*_ = 0.63 (30% ethyl acetate:hexane). ^1^H NMR (600 MHz, CDCl_3_) δ 7.50–7.33 (m, 10H, Ar), 7.22 –7.06 (m, 15H, Ar), 6.97 (d, *J =* 8.3 Hz, 3H, Ar), 5.88 (ddd, *J =* 17.5, 10.5, 7.4 Hz, 1H, -C*H*=CH_2_), 5.27 (dd, *J =* 17.3, 8.7 Hz, 2H, -CH=C*H*_2_), 5.00 (d, *J =* 3.5 Hz, 1H, H-1′), 4.80 (d, *J =* 11.5 Hz, 1H, OCH_2_Ph), 4.74 (s, 1H, OCH_2_Ph), 4.72 (s, 1H, OCH_2_Ph), 4.70 (s, 1H, OCH_2_Ph), 4.68 (s, 1H, OCH_2_Ph), 4.66 (s, 1H, OCH_2_Ph), 4.64 (s, 1H, OCH_2_Ph), 4.62 (s, 1H, OCH_2_Ph), 4.54 (d, *J =* 12.4 Hz, 2H, OCH_2_Ph), 4.49 (dd, *J =* 12.9, 5.6 Hz, 4H, OCH_2_Ph), 4.45 (d, *J =* 12.2 Hz, 1H, OCH_2_Ph), 4.41 (s, 1H, OCH_2_Ph), 4.39 (d, *J =* 3.5 Hz, 1H, OCH_2_Ph), 4.37 (d, *J =* 9.2 Hz, 1H, OCH_2_Ph), 4.34 (s, 1H), 4.23 (d, *J =* 12.0 Hz, 1H, H-2), 4.20 (d, *J =* 7.0 Hz, 1H), 4.14 (t, *J =* 7.1 Hz, 1H), 4.10 (d, *J =* 5.2 Hz, 1H, H-6b), 4.01 (ddd, *J =* 10.2, 3.2, 2.1 Hz, 1H, H-5′), 3.90 (dt, *J =* 18.8, 7.0 Hz, 3H, H-3′, H-6b′, H-6a), 3.73 (dd, *J =* 5.4, 4.4 Hz, 1H, H-3), and 3.60–3.45 (m, 3H, H-2′, H-4′, H-6a′). ^13^C NMR (151 MHz, CDCl_3_) δ 137.42 (Ar), 137.05 (Ar), 137.04 (Ar), 136.91 (Ar), 136.81 (Ar), 136.66 (Ar), 135.06 (Ar), 131.61 (Ar), 131.61 (Ar), 131.60 (Ar), 131.59 (Ar), 131.58 (Ar), 131.55 (Ar), 131.50 (Ar), 131.49 (Ar), 129.65 (Ar), 129.49 (Ar), 129.37 (Ar), 129.28 (Ar), 129.27 (Ar), 129.16 (Ar), 121.57 (Ar), 121.52 (Ar), 119.33 (=CH_2_), 98.43 (C1′), 81.66 (C3), 81.39 (C2′), 80.62 (C3), 79.83 (C2′), 78.96 (C4′), 77.78 (-OCH_2_Ph), 77.08 (-OCH_2_Ph), 76.87 (-OCH_2_Ph), 74.55 (-OCH_2_Ph), 74.22 (-OCH_2_Ph), 73.50 (-OCH_2_Ph), 72.71 (-OCH_2_Ph), 72.54 (-OCH_2_Ph), 72.52 (-OCH_2_Ph), 71.29 (-OCH_2_Ph), 71.19 (-OCH_2_Ph), 70.85 (C5′), 69.77 (C6′), and 68.28 (C6). Mass spectrum (HRMS), *m/z*=1545.8219 (M+Na)^+^; C_62_H_59_Br_7_O_10_ requires 1545.8451 (M+Na)^+^.

#### 3,4,7-Tri-*O*-(4-bromobenzyl)-5-*O*-(2′,3′,4′,6′-tetra-*O*-(4-bromobenzyl)-α-D-glucopyranosyl)-D-gluchept-1-enone (18′)

Compound **17′** (1.20 g, 0.78 mmol) was dissolved by gentle warming in anhydrous toluene (3.0 ml). To this solution was added dry dimethyl sulfoxide (3.0 ml). To the clear solution was added anhydrous pyridine (62.5 µl, 0.78 mmol), trifluoracetic acid (23.65 µl, 0.39 mmol), and *N,N′*-dicyclohexylcarbodiimide (0.48 g, 2.34 mmol) in that order. The reaction was left to stir at room temperature for 18 h. After completion of reaction, which was monitored by TLC, toluene (10 ml) was added. The resulting crystalline dicyclohexylurea was removed by filtration and washed with benzene. The combined filtrates and washings were extracted with water (20 ml X 3) to remove dimethyl sulfoxide. The organic layer was dried over anhydrous Na_2_SO_4_, evaporated under reduced pressure, and subjected to flash column chromatography on a silica gel with 1:6 ethyl acetate–hexane to give product as colorless viscous liquid **18′**: yield 88% (1.1 g); silica gel TLC *R*_*f*_ = 0.72 (30% ethyl acetate:hexane). ^1^H NMR (600 MHz, CDCl_3_) δ 7.51–7.31 (m, 16H), 7.17–6.96 (m, 12H), 5.91 (ddd, *J =* 17.4, 10.5, 6.9 Hz, 1H, H-1), 5.35–5.27 (m, 1H, =CH_2_), 4.90–4.84 (m, 2H, OCH_2_Ph, H-1**′**), 4.82–4.75 (m, 1H, OCH_2_Ph), 4.71 (dd, *J =* 11.7, 3.9 Hz, 1H, OCH_2_Ph), 4.68 (d, *J =* 11.4 Hz, 1H, OCH_2_Ph), 4.55 (d, *J =* 4.3 Hz, 1H, OCH_2_Ph), 4.55–4.54 (m, 1H, OCH_2_Ph), 4.53 (d, *J =* 4.4 Hz, 1H, OCH_2_Ph), 4.46 (dd, *J =* 12.2, 9.0 Hz, 1H, H-6b), 4.38 (s, 1H, OCH_2_Ph), 4.36 (s, 1H, OCH_2_Ph), 4.33 (s, 1H, OCH_2_Ph), 4.31 (d, *J =* 5.0 Hz, 1H, OCH_2_Ph), 4.28 (d, *J =* 4.9 Hz, 1H, OCH_2_Ph), 4.25 (s, 1H, OCH_2_Ph), 4.23 (s, 1H, OCH_2_Ph), 4.20 (d, *J =* 6.6 Hz, 1H, OCH_2_Ph), 4.15 (s, 1H, OCH_2_Ph), 4.12 (s, 1H, H-6a), 4.08 (dd, *J =* 14.5, 5.4 Hz, 1H, H-3′), 3.95–3.89 (m, 1H, H-5′), 3.85 (d, *J =* 10.1 Hz, 1H, H-2), 3.78 (dd, *J =* 6.6, 4.3 Hz, 1H, H-3), 3.65–3.55 (m, 1H, H-6a′), 3.46 (dd, *J =* 9.8, 3.7 Hz, 1H, H-2′), and 3.37 (dd, *J =* 10.8, 1.9 Hz, 1H, H-6b′). ^13^C NMR (151 MHz, CDCl_3_) δ 203.97 (-C=O), 137.47 (Ar), 137.15 (Ar), 136.91 (Ar), 136.90 (Ar), 136.69 (Ar), 136.55 (Ar), 136.37 (Ar), 134.34 (Ar), 131.61 (Ar), 131.60 (Ar), 131.59 (Ar), 131.58 (Ar), 131.55 (Ar), 131.53 (Ar), 131.52 (Ar), 131.51 (Ar), 131.51 (Ar), 131.49 (Ar), 131.47 (Ar), 131.46 (Ar), 129.67 (Ar), 129.64 (Ar), 129.63 (Ar), 129.55 (Ar), 129.35 (Ar), 129.34 (Ar), 129.34 (Ar), 129.32 (Ar), 129.30 (Ar), 129.30 (Ar), 129.23 (Ar), 129.02 (Ar), 129.01 (Ar), 129.00 (Ar), 129.00 (Ar), 128.99 (Ar), 121.81 (Ar), 121.77 (Ar), 121.75 (Ar), 121.73 (Ar), 121.69 (Ar), 121.57(Ar), 119.47 (=CH_2_), 99.46 (C-1′), 81.40 (C3), 81.07 (C2′), 80.70 (C3′, C2), 79.82 (C4′), 79.07 (OCH_2_Ph), 77.50 (OCH_2_Ph), 77.40 (OCH_2_Ph), 77.19 (OCH_2_Ph), 76.97 (OCH_2_Ph), 74.63 (OCH_2_Ph), 74.38 (OCH_2_Ph), 73.99 (OCH_2_Ph), 73.83 (OCH_2_Ph), 72.64 (OCH_2_Ph), 72.51 (OCH_2_Ph), 72.31 (OCH_2_Ph), 70.96 (C5′), 69.95 (C6′), and 67.98 (C6). Mass spectrum (HRMS), *m/z*=1543.7928 (M+Na)^+^; C_62_H_57_Br_7_O_10_ requires 1543.8105 (M+Na)^+^.

#### 3,4,9-Tri-*O*-(4-bromobenzyl)-5-*O*-(2′,3′,4′,6′-tetra-*O*-(4-bromobenzyl)-α-D-glucopyranosyl)-D-gluco-octa-1,7-dienitol (19A′)

To a cooled (−78°C) solution of **18′** (1.08 g, 0.71 mmol) in tetrahydrofuran (15 ml) was added of vinylmagnesium bromide (0.7 M) in tetrahydrofuran (4.57 ml, 3.20 mmol) dropwise. The reaction mixture was stirred for 1 h at the same temperature. The reaction mixture was warmed to room temperature. Diethyl ether (30 ml) and aq. NH_4_Cl (30 ml) were added to the reaction mixture. The organic layer was separated, washed with brine (50 ml X 2), and dried over anhydrous Na_2_SO_4_. The solvent was evaporated under reduced pressure and purification was performed by flash column chromatography on a silica gel with 1:8 ethyl acetate:hexane to afford product as colorless viscous liquid **19A′**: yield 93% (0.97 g); silica gel TLC *R*_*f*_ = 0.65 (30% ethyl acetate:hexane). ^1^H NMR (600 MHz, CDCl3) δ 7.49–7.31 (m, 16H, Ar), 7.15–7.06 (m, 12H, Ar), 6.09 (dd, *J =* 17.4, 10.9 Hz, 1H, H-7), 5.65 (ddd, *J =* 17.8, 10.3, 7.7 Hz, 1H, H-2), 5.53 (dd, *J =* 17.4, 1.6 Hz, 1H, H-8a), 5.26 (ddd, *J =* 21.1, 8.9, 2.5 Hz, 4H, H-1′, H-1b, H-8b, H-1a), 4.80 (dd, *J =* 22.5, 11.3 Hz, 3H, OCH_2_Ph), 4.67 (dd, *J =* 21.9, 11.3 Hz, 3H, OCH_2_Ph), 4.54 (d, *J =* 11.2 Hz, 1H, OCH_2_Ph), 4.42 (dd, *J =* 16.8, 12.0 Hz, 4H, OCH_2_Ph), 4.37 (dd, *J =* 16.3, 11.8 Hz, 4H, OCH_2_Ph), 4.33 (s, 1H, OCH_2_Ph), 4.28 (d, *J =* 12.4 Hz, 3H, OCH_2_Ph), 4.20 (t, *J =* 8.2 Hz, 1H, H-3), 4.15 (d, *J =* 12.0 Hz, 1H, OCH_2_Ph), 3.95 (d, *J =* 1.2 Hz, 1H, OCH_2_Ph), 3.89–3.82 (m, 2H, H-5, H-3′), 3.79 (d, *J =* 10.1 Hz, 1H, H-5′), 3.63 (d, *J =* 8.8 Hz, 1H, H-4), 3.57–3.52 (m, 1H, H-4′, H-9a), 3.49 (dd, *J =* 9.7, 3.5 Hz, 1H, H-2′), and 3.33–3.22 (m, 3H, H-6a′, H-6b′, H-9b). ^13^C NMR (151 MHz, CDCl_3_) δ 139.19 (Ar), 137.65 (Ar), 137.45 (Ar), 137.35 (Ar), 137.01 (Ar), 136.88 (Ar), 136.71 (Ar), 136.47 (Ar), 135.47 (Ar), 131.57 (Ar), 131.52 (Ar), 131.44 (Ar), 131.42 (Ar), 131.33 (Ar), 129.81 (Ar), 129.70 (Ar), 129.27 (Ar), 129.22 (Ar), 129.16 (Ar), 129.08 (Ar), 128.92 (Ar), 121.92 (Ar), 121.72 (Ar), 121.56 (Ar), 121.49 (Ar), 121.33 (Ar), 121.32 (Ar), 120.06 (Ar), 116.20 (Ar), 95.86 (C1′), 83.64 (C5), 81.49 (C2′), 79.75 (C3′, C4), 79.36 (C4′), 77.43 (OCH_2_Ph), 77.33 (OCH_2_Ph), 77.03 (OCH_2_Ph), 76.81 (OCH_2_Ph), 74.84 (OCH_2_Ph), 74.54 (OCH_2_Ph), 74.19 (OCH_2_Ph), 73.95, 72.74 (OCH_2_Ph), 72.25 (OCH_2_Ph), 71.67 (OCH_2_Ph), 70.56 (C5′), 69.71 (C6′), and 67.68 (C9). Mass spectrum (HRMS), *m/z* = 1572.5124 (M + Na)^+^; C_64_H_61_Br_7_O_10_ requires 1572.5223 (M + Na)^+^.

#### 3,4,9-Tri-*O*-(4-bromobenzyl)-5-*O*-(2′,3′,4′,6′-tetra-*O*-(4-bromobenzyl)-α-D-glucopyranosyl)-L-ido-octa-1,7-dienitol (19B′)

Flash column chromatography on a silica gel with 1:6 ethyl acetate–hexane afforded product as colorless liquid **19B′**: yield 23% (0.24 g); silica gel TLC *R*_*f*_ = 0.72 (30% ethyl acetate:hexane). ^1^H NMR (600 MHz, CDCl_3_) δ 7.51 –7.30 (m, 15H, Ar), 7.19–6.85 (m, 13H, Ar)6.16 (dd, *J =* 17.3, 11.0 Hz, 1H, H-7), 5.76 (ddd, *J =* 17.2, 10.4, 8.4 Hz, 1H, H-2), 5.59 (dd, *J =* 17.4, 1.8 Hz, 1H, H-8a), 5.41 (d, *J =* 3.4 Hz, 1H, H-1′), 5.32 (d, *J =* 1.7 Hz, 1H, H-1b), 5.31–5.25 (m, 2H, H-8b, H-1a), 4.79 (dd, *J =* 26.0, 11.3 Hz, 2H, OCH_2_Ph), 4.66 (dd, *J =* 11.3, 2.8 Hz, 2H, OCH_2_Ph), 4.47 (d, *J =* 11.3 Hz, 1H, OCH_2_Ph), 4.45–4.39 (m, 2H, OCH_2_Ph), 4.38 (s, 1H, OCH_2_Ph), 4.34 (d, *J =* 12.1 Hz, 2H, OCH_2_Ph), 4.28–4.25 (m, 1H, H-3), 4.24 (d, *J =* 2.3 Hz, 1H, OCH_2_Ph), 4.19–4.11 (m, 2H, H-5, H-3), 3.97 (d, *J =* 1.8 Hz, 1H, H-4), 3.87 (dd, *J =* 8.4, 1.9 Hz, 1H, H-3′), 3.78 (t, *J =* 9.9 Hz, 2H, H-5′, H-9a), 3.64 (d, *J =* 10.1 Hz, 1H, H-7a), 3.57–3.52 (m, 1H, H-4′), 3.45 (dd, *J =* 9.6, 3.4 Hz, 1H, H-2′), 3.24 (d, *J =* 9.2 Hz, 1H, H-6b′), 3.19 (dd, *J =* 5.5, 2.2 Hz, 2H, H-6a′, H-9b), and 2.74 (s, 1H, OH). ^13^C NMR (151 MHz, CDCl_3_) δ 139.19 (Ar), 138.14 (Ar), 137.46 (Ar), 137.25 (Ar), 136.89 (Ar), 136.57 (Ar), 131.56 (Ar), 131.47 (Ar), 131.45 (Ar), 131.38 (Ar), 131.27 (Ar), 131.14 (Ar), 129.90 (Ar), 129.41 (Ar), 129.28 (Ar),129.25 (Ar), 129.18 (Ar),128.78 (Ar), 128.60 (Ar), 121.98 (Ar), 121.74 (Ar), 121.52 (Ar), 121.49 (Ar), 121.31 (Ar), 121.20 (Ar), 120.06 (Ar), 116.20 (Ar), 97.45 (C1′), 83.52 (C5), 81.41 (C2′), 79.75 (C4), 79.30 (C4′), 77.43 (C3′), 77.33 (OCH_2_Ph), 77.03 (OCH_2_Ph), 76.98 (OCH_2_Ph), 74.94 (OCH_2_Ph), 74.78 (OCH_2_Ph), 74.02 (C5), 73.55 (OCH_2_Ph), 72.74 (OCH_2_Ph), 72.25 (OCH_2_Ph), 71.67 (OCH_2_Ph), 70.56 (C5′), 68.91 (C9), and 67.89 (C6′). Mass spectrum (ESI-MS), *m/z* = 1572.4 (M + Na)^+^; C_64_H_61_Br_7_O_10_ requires 1572.52 (M + Na)^+^.

#### (1,3,4/2)-1,2-Di-*O*-(4-bromobenzyl)-4-*C*-[(4-bromobenzyloxy)methyl]-3-*O*-(2′,3′,4′,6′-tetra-*O*-(4-bromobenzyl)-α-D-glucopyranosyl)cyclohex-5-ene-1,2,3,4-tetrol (10′)

A solution of dialkene **19A′** in dichloromethane was degassed by passing nitrogen gas through it for 20 min. After that, 1st generation Grubbs catalyst (10 mol%) was added to the solution and the reaction was kept under nitrogen atmosphere using nitrogen balloon for 7 days or until the catalyst turned dark brown or black. Then, everything was evaporated under reduced pressure and purified using flash column chromatography on a silica gel. **10′**: yield 80% (0.60 g); silica gel TLC *R*_*f*_ = 0.5 (30% ethyl acetate:hexane). ^1^H NMR (600 MHz, CDCl_3_) δ 7.41–7.14 (m, 18H, Ar) 7.11–6.92 (m, 10H, Ar) 5.92 (dd, *J =* 10.2, 1.9 Hz, 1H, H-1), 5.67 (dd, *J =* 10.2, 1.7 Hz, 1H, H-6), 5.54 (d, *J =* 3.6 Hz, 1H, H-1′), 4.81 (d, *J =* 12.2 Hz, 1H, OCH_2_Ph), 4.75–4.65 (m, 3H, OCH_2_Ph), 4.61 (dd, *J =* 11.6, 7.7 Hz, 2H, OCH_2_Ph), 4.52–4.46 (m, 2H, OCH_2_Ph), 4.40 (d, *J =* 2.5 Hz, 4H, OCH_2_Ph), 4.35 (d, *J =* 11.3 Hz, 1H, OCH_2_Ph), 4.27 (d, *J =* 12.4 Hz, 1H, OCH_2_Ph), 4.17–4.13 (m, 1H, H-2), 4.10 (d, *J =* 3.1 Hz, 2H, H-3, H-4), 3.84 (dd, *J =* 20.7, 10.9 Hz, 2H, H-3′, H-5′), 3.64 (d, *J =* 9.1 Hz, 1H, H-7a), 3.52 (d, *J =* 9.4 Hz, 1H, H-4′), 3.44 (dd, *J =* 10.2, 3.6 Hz, 2H, H-2′, H-6a′), 3.38 (d, *J =* 1.8 Hz, 1H, H-7b), and 3.38–3.36 (m, 1H, H-6b′). ^13^C NMR (151 MHz, CDCl_3_) δ 137.89 (Ar), 137.31 (Ar), 137.02 (Ar), 136.90 (Ar), 136.78 (Ar), 136.68 (Ar), 136.50 (Ar), 131.56 (Ar), 131.52 (C6), 131.51 (Ar), 131.49 (Ar), 131.32 (C1), 130.76 (Ar), 130.00 (Ar), 129.66 (Ar), 129.28 (Ar), 129.21 (Ar), 129.11 (Ar), 128.99 (Ar), 128.22 (Ar), 121.85 (Ar), 121.77 (Ar), 121.70 (Ar), 121.60 (Ar), 121.59 (Ar), 121.55 (Ar), 120.99 (Ar), 96.86 (C1′), 81.59 (C3′), 80.68 (C3), 80.03 (C2′), 79.66 (C2), 77.48 (C5), 77.26 (C4′) (OCH_2_Ph), 77.05 (OCH_2_Ph), 76.83 (OCH_2_Ph), 74.85 (OCH_2_Ph), 74.55 (C4), 74.15 (OCH_2_Ph), 73.72 (OCH_2_Ph), 73.34 (C7), 73.01 (OCH_2_Ph), 72.73 (OCH_2_Ph), 72.36 (OCH_2_Ph), 72.13 (OCH_2_Ph), 70.72 (C5′), and 68.11 (C6′). Mass spectrum (HRMS), *m/z* = 1544.6995 (M + Na)^+^; C_62_H_57_Br_7_O_10_ requires 1544.7132 (M + Na)^+^.

#### (1,3,4/2)-1,2-Di-*O*-benzyl-4-C-[(benzyloxy)methyl]-4*-O-*acetyl-3*-O-*(2′,3′,4′,6′-tetra*-O-*benzyl-α-D-glucopyranosyl)cyclohex-5-ene-1,2,3,4-tetrol (20)

To a cooled (0°C) solution of **10** (0.200 g, 0.206 mmol) in tetrahydrofuran (5 ml) was added 200 µl of (2M sodium bis(trimethylsilyl) amide in tetrahydrofuran) dropwise. The reaction mixture was stirred for 30 min at the same temperature. After that acetyl chloride (19 μl, 0.268 mmol, 1.3 eq) was added to the reaction and let it run for 24 h at room temperature. After completion of the reaction, which was monitored by TLC, ethyl acetate (6 ml) was added to the reaction mixture. Washings were given to the organic layer with NaHCO_3_ solution (20 ml) and brine (20 ml), dried over anhydrous Na_2_SO_4_. The solvent was evaporated under reduced pressure and purification was performed by flash column chromatography on a silica gel to afford product as colorless viscous liquid **20**: yield (0.107 g, 52%); *R*_*f*_ = 0.79 (30% ethyl acetate:hexane). ^1^H NMR (600 MHz, CDCl_3_): δ 7.33–7.22 (m, 33H, Ar), 7.18–7.16 (m, 2H, Ar), 6.24 (dd, 1H, *J =* 10.3, 1.3, H-5′), 5.96 (dd, 1H, *J =* 10.3, 1.8, H-6′), 5.42 (d, 1H, *J =* 3.5, H-1), 5.1 (m, 1H, OCH_2_Ph), 4.87 (m, 3H, OCH_2_Ph), 4.76 (m, 1H, OCH_2_Ph), 4.66 (s, 2H, OCH_2_Ph), 4.53 (m, 5H, OCH_2_Ph), 4.45 (m, 1H, OCH_2_Ph), 4.38 (d, 1H, *J =* 12.1, OCH_2_Ph), 4.2 (m, 2H, H-1′, H-2′), 4.11 (s, 2H, H-7a′, 7-b’), 4.06 (m, 2H, H-3, H-5), 3.63 (m, 2H, H-4, H-6a), 3.52 (m, 2H, H-2, H-6b), and 2.03 (s, 3H, CH3). ^13^C NMR (150 MHz^,^ CDCl_3_): δ 169.85 (C=O), 139.42 (Ar), 138.80 (Ar), 138.58 (Ar), 138.38 (Ar), 138.21 (Ar), 138.05 (Ar), 137.91 (Ar), 130.95 (Ar), 129.42 (Ar), 128.41 (Ar), 128.38 (Ar), 128.33 (Ar), 128.19 (Ar), 128.08 (Ar), 127.98 (Ar), 127.97 (Ar), 127.71 (Ar), 127.67 (Ar), 127.60 (Ar), 127.52 (Ar), 127.44 (Ar), 127.42 (Ar), 127.06 (Ar), 126.98 (Ar), 98.40 (C1′), 81.75 (C3′), 81.26 (C2′), 81.18 (C3), 80.01 (C2), 79.67 (OCH_2_Ph), 77.74 (C4′), 77.37 (OCH_2_Ph), 75.47 (C5), 75.11 (OCH_2_Ph), 74.74 (OCH_2_Ph), 73.47 (C4), 73.09 (OCH_2_Ph), 72.12 (OCH_2_Ph), 70.91 (C5′), 69.51 (C7), 68.68 (C6′), and 21.90 (CH3). Mass spectrum (HRMS), *m/z* = 1033.4484 (M + Na)^+^; C_64_H_66_O_11_ requires 1033.4503 (M + Na)^+^.

#### (1,3,4/2)-1,2-Di*-O-*(4-bromobenzyl)-4-C-[(4-bromobenzyloxy)methyl]-4*-O-*acetyl-3*-O-*(2′,3′,4′,6′-tetra*-O-*(4-bromobenzyl)-α-D-glucopyranosyl)cyclohex-5-ene-1,2,3,4-tetrol (20′)

A solution of **10′** (0.58 g, 0.37 mmol) in tetrahydrofuran (10 ml) was cooled at 0°C. To this, excess (1.50 mmol) potassium bis(trimethylsilyl) amide (1.0 M) was added slowly. The reaction mixture was stirred for 45 min at the same temperature. 1.50 mmol of acetyl chloride was added dropwise to the reaction mixture. The reaction mixture was warmed to room temperature and stirred for 10–12 h. After completion of the reaction, ethyl acetate was added. The organic layer was washed with aq. brine and dried over anhydrous Na_2_SO_4_. The solvent was evaporated under reduced pressure and purification was performed by flash column chromatography on a silica gel to afford product as colorless viscous liquid **20′**: yield 80% (0.38 g); silica gel TLC *R*_*f*_ = 0.25 (30% ethyl acetate:hexane). ^1^H NMR (600 MHz, CDCl_3_) δ 7.45–7.39 (m, 15H, Ar), 7.35 (dd, *J =* 8.3, 6.3 Hz, 2H, Ar), 7.15–6.96 (m, 11H, Ar) 6.18 (d, *J =* 10.5 Hz, 1H, H-5′), 5.94 (d, *J =* 10.6 Hz, 1H, H-6′), 5.34 (d, *J =* 3.5 Hz, 1H, H-1), 4.90 (d, *J =* 12.2 Hz, 1H, OCH_2_Ph), 4.70 (dd, *J =* 11.7, 3.7 Hz, 2H, OCH_2_Ph), 4.70 (dd, *J =* 9.2, 6.0 Hz, 3H, OCH_2_Ph), 4.67 (s, 1H, OCH_2_Ph), 4.59 (dd, *J =* 11.6, 5.5 Hz, 3H, OCH_2_Ph), 4.50 (dd, *J =* 13.9, 12.2 Hz, 3H, OCH_2_Ph), 4.42–4.35 (m, 7H, OCH_2_Ph), 4.29 (d, *J =* 12.3 Hz, 2H, H-1′, H-2′), 4.10 (d, *J =* 4.7 Hz, 2H, H-7a, H-7b), 4.02 (dd, *J =* 24.7, 8.9 Hz, 2H, H-3, H-5), 3.92 (dd, *J =* 15.1, 5.7 Hz, 2H, H-4), 3.53 (d, *J =* 9.8 Hz, 1H, H-6a), 3.52–3.47 (m, 1H, H-2), 3.43 (dd, *J =* 9.8, 3.4 Hz, 1H, H-6b), and 2.03 (s, 3H, CH_3_). ^13^C NMR (151 MHz, CDCl_3_) δ 169.61 (C=O), 138.34 (Ar), 137.55 (Ar), 137.23 (Ar), 137.01 (Ar), 136.99 (Ar), 136.91 (Ar), 136.62 (Ar), 131.57 (Ar), 131.57 (Ar), 131.55 (Ar), 131.55 (Ar), 131.51 (Ar), 131.51 (Ar), 131.37 (Ar), 131.20 (Ar), 130.56 (Ar), 129.55 (Ar), 129.42 (Ar), 129.13 (Ar), 129.13 (Ar), 129.05 (Ar), 129.00 (Ar), 128.27 (Ar), 121.84 (Ar), 121.76 (Ar), 121.63 (Ar), 121.60 (Ar), 121.56 (Ar), 121.47 (Ar), 120.90 (Ar), 98.43 (C1′), 81.48 (C2′), 81.12 (C3), 81.05 (C3′), 80.94 (C2), 80.12 (OCH_2_Ph), 79.56 (OCH_2_Ph), 77.02 (OCH_2_Ph), 76.81 (C5), 74.49 (C4′), 74.27 (OCH_2_Ph), 72.69 (C4), 72.48 (OCH_2_Ph), 71.72 (C7), 71.13 (OCH_2_Ph), 70.88 (C5′), 68.62 (C6′), and 21.82 (CH_3_). Mass spectrum (HRMS), *m/z* = 1585.9246 (M + Na)^+^; C_64_H_59_Br_7_O_11_ requires 1585.9117 (M + Na)^+^.

#### (1,3,4/2)-1,2-Di*-O-*benzyl-4-C-[(benzyloxy)methyl]-3*-O-*(2′,3′,4′,6′-tetra*-O-*benzyl-α-D-glucopyranosyl)cyclohex-4-ene-1,2,3,6-tetrol (21)

To a solution of **20** (100 mg, 0.099 mmol) in ethyl acetate (4 ml) was added bis(benzonitrile) palladium (II) chloride (5 mg, 0.013 mmol, 10 mol %), and reaction was left for stirring under refluxing conditions for 36 h. The solvent was evaporated under reduced pressure and purification was performed by flash column chromatography on a silica gel (7% ethyl acetate:hexane) to afford product as colorless viscous liquid. *R*_*f*_ = 0.78 (30% ethyl acetate:hexane). ^1^H NMR (600 MHz, CDCl_3_): δ 7.34–7.2 (m, 33H, Ar), 7.15 (m, 2H, Ar), 5.98 (m, 1H, H-5), 5.63 (d, 1H, *J =* 3.7, H-1′), 4.96 (d, 1H, *J =* 11.6, OCH_2_Ph), 4.91 (d, 1H, *J =* 11.1, OCH_2_Ph), 4.82 (d, 2H, *J =* 9.4, OCH_2_Ph), 4.73 (d, 1H, *J =* 11.7, OCH_2_Ph), 4.59 (m, 6H, OCH_2_Ph, H-3), 4.45 (m, 2H, OCH_2_Ph), 4.37 (d, 1H, *J =* 12.1, OCH_2_Ph), 4.29 (d, 1H, *J =* 12.7, H-7a), 4.23 (dd, 1H, *J =* 9.2, 6.2, H-2), 3.98 (m, 2H, H-3′, H-7b), 3.91 (m, 1H, H-5′), 3.73 (dd, 1H, *J =* 9.3, 3.8, H-1), 3.69 (t, 1H, *J =* 9.5, H-4′), 3.57 (td, 2H, *J =* 9.4, 3.3, H-2, H-6a**′**), 3.42 (dd, 1H, *J =* 10.8, 1.7, H-6b**′**), and 2.13 (s, 3H, CH3); 13C NMR (150 MHz, CDCl_3_): δ 170.84 (C=O), 140.45 (Ar), 138.88 (Ar), 138.76 (Ar), 138.62 (Ar), 138.16 (Ar), 138.12 (Ar), 138.00 (Ar), 128.49 (Ar), 128.46 (Ar), 128.43 (Ar), 128.23 (Ar), 128.16 (Ar), 127.98 (Ar), 127.93 (Ar), 127.89 (Ar), 127.86 (Ar), 127.82 (Ar), 127.79 (Ar), 127.76 (Ar), 127.74 (Ar), 127.68 (Ar), 127.41 (Ar), 127.01 (Ar), 122.92 (C5′), 97.06 (C1′), 82.27 (C3′), 79.79 (C2), 79.57 (C2′), 77.96 (C4′), 75.65 (OCH_2_Ph), 75.07 (OCH_2_Ph), 74.64 (OCH_2_Ph), 73.74 (OCH_2_Ph), 73.66 (OCH_2_Ph), 73.10 (OCH_2_Ph), 72.45 (OCH_2_Ph), 72.45 (OCH_2_Ph), 72.13 (C5′), 70.45 (C7), 68.35 (C6′), and 21.40 (CH3). Mass spectrum (HRMS), *m/z* = 1033.499 (M + Na)^+^; C_64_H_66_O_11_ requires 1033.450 (M + Na)^+^. To this viscous product, in methanol (8 ml), was added a small piece of sodium and the reaction was left to run for 40 min at room temperature under N^2^ atmosphere. The completion of reaction was monitored by TLC after which the reaction mixture was neutralized to pH = 7 with amberlite resin. Resin was filtered off followed by the evaporation of solvent under reduced pressure, and purification was performed by flash column chromatography on a silica gel (20% ethyl acetate:hexane) to afford product as colorless viscous liquid **21**. Yield (58 mg, 60% over two steps); *R_f_* = 0.38 (30% ethyl acetate:hexane). ^1^H NMR (600 MHz, CDCl_3_): δ 7.35–7.12 (m, 35H, Ar) 5.98 (d, *J =* 2.8 Hz, 1H, H-5), 5.36 (d, *J =* 3.7 Hz, 1H, H-1′), 4.80 (dd, *J =* 11.3, 2.8 Hz, 2H, OCH_2_Ph), 4.75 (dd, *J =* 11.5, 9.2 Hz, 2H, OCH_2_Ph), 4.71–4.64 (m, 3H, OCH_2_Ph), 4.60–4.52 (m, 2H, OCH_2_Ph), 4.43 (m, 8H, H-3, OCH_2_Ph), 4.33–4.28 (m, 1H, H-7a), 4.18 (dd, *J =* 6.9, 4.0 Hz, 1H, H-2), 3.95 (m, 3H, H-3′, H-5′, H-7b), 3.70 (m, 2H, H-1, H-4′), 3.60 (dd, *J =* 10.7, 3.0 Hz, 1H, H-6a′), 3.57 (dd, *J =* 9.9, 3.7 Hz, 1H, H-2′), and 3.45 (dd, *J =* 10.6, 1.9 Hz, 1H, H-6b′). 13C NMR (150 MHz, CDCl3): δ 138.70 (Ar), 138.46 (Ar), 138.35 (Ar), 138.23 (Ar), 138.03 (Ar), 137.94 (Ar), 137.32 (Ar), 136.09 (Ar), 128.44 (Ar), 128.40 (Ar), 128.35 (Ar), 128.32 (Ar), 128.28 (Ar), 128.02 (Ar), 127.91 (Ar), 127.86 (Ar), 127.75 (Ar), 127.73 (Ar), 127.70 (Ar), 127.69 (Ar), 127.57 (Ar), 127.52 (Ar), 127.02 (Ar), 97.75 (C1′), 82.00 (C3′), 79.61 (C2, C2′), 76.81 (C4), 75.37 (OCH_2_Ph), 74.89 (OCH_2_Ph), 74.00 (OCH_2_Ph), 73.83 (OCH_2_Ph), 73.49 (OCH_2_Ph), 71.96 (OCH_2_Ph), 71.77 (C5′), 71.19 (C7), and 64.79 (C6′). Mass spectrum (HRMS), *m/z* = 991.418 (M + 23)^+^; C_62_H_64_O_10_ requires 991.439 (M + 23)^+^.

#### (1,3,4/2)-1,2-Di*-O-*(4-bromobenzyl)-4-C-[(4-bromobenzyloxy)methyl]-3*-O-*(2′,3′,4′,6′-tetra*-O-*(4-bromobenzyl)-α-D-glucopyranosyl)cyclohex-4-ene-1,2,3,6-tetrol (21′)

To a solution of **20′** (0.30 mg, 0.19 mmol) in ethyl acetate (6 ml) was added bis(benzonitrile) palladium (II) chloride (6 mg, 0.019 mmol, and 10 mol %), and the reaction was left for stirring under refluxing conditions for 12 h. After confirming the completion of the reaction, the mixture was filtered to remove the deactivated palladium catalyst. The solvent was evaporated under reduced pressure and the crude was dissolved in methanol. Sodium methoxide was added till the pH reached 9. The reaction mixture was stirred at room temperature for 45 min. The completion of reaction was monitored by TLC after which the reaction mixture was neutralized to pH = 7 with amberlite resin. Resin was filtered off followed by the evaporation of the solvent under reduced pressure and purification was performed by flash column chromatography on a silica gel (20% ethyl acetate:hexane) to afford product as colorless viscous liquid **20′**: yield 80% (0.15 g); silica gel TLC *R*_*f*_ = 0.43 (30% ethyl acetate:hexane). ^1^H NMR (600 MHz, CDCl_3_) δ 7.48–7.34 (m, 15H, Ar), 7.24–7.12 (m, 8H, Ar), 7.09–6.88 (m, 5H, Ar), 5.99 (d, *J =* 4.1 Hz, 1H, H-5), 5.27 (d, *J =* 3.4 Hz, 1H, H-1′), 4.94–4.87 (m, 1H, OCH_2_Ph), 4.84 (d, *J =* 11.4 Hz, 1H, OCH_2_Ph), 4.73 (dd, *J =* 9.5, 5.2 Hz, 1H, OCH_2_Ph), 4.69 (dd, *J =* 11.9, 4.5 Hz, 1H, OCH_2_Ph), 4.66 (t, *J =* 4.1 Hz, 1H, OCH_2_Ph), 4.64–4.61 (m, 2H, OCH_2_Ph), 4.59 (s, 1H, OCH_2_Ph), 4.57 (d, *J =* 5.6 Hz, 1H, OCH_2_Ph), 4.56–4.53 (m, 1H, OCH_2_Ph), 4.52 (d, *J =* 12.4 Hz, 1H, OCH_2_Ph), 4.48 (d, *J =* 5.8 Hz, 1H, OCH_2_Ph), 4.43 (t, *J =* 5.6 Hz, 1H, OCH_2_Ph), 4.40 (d, *J =* 5.2 Hz, 1H, OCH_2_Ph), 4.38 (t, *J =* 6.1 Hz, 1H, OCH_2_Ph), 4.36 (d, *J =* 4.6 Hz, 1H, H-7a), 4.09 (dd, *J =* 7.6, 4.9 Hz, 1H, H-2), 4.01 (t, *J =* 9.9 Hz, 1H), 3.93 (dd, *J =* 11.0, 7.2 Hz, 1H, H-3′), 3.87–3.75 (m, 1H, H-5′), 3.70–3.61 (m, 1H, H-7b), 3.56 (ddd, *J =* 14.6, 13.4, 8.6 Hz, 1H, H-1), 3.47 (dd, *J =* 9.7, 3.3 Hz, 1H, H-4′), and 3.32 (d, *J =* 10.4 Hz, 2H, H-6a, H-6b). 13C NMR (151 MHz, CDCl_3_) δ 137.34 (Ar), 137.23 (Ar), 136.98 (Ar), 136.70 (Ar), 136.63 (Ar), 131.69 (Ar), 131.60 (Ar), 131.56 (Ar), 131.53 (Ar), 131.51 (Ar), 131.48 (Ar), 129.71 (Ar), 129.64 (Ar), 129.60 (Ar), 129.58 (Ar), 129.56 (Ar), 129.36 (Ar), 129.27 (Ar), 129.26 (Ar), 129.23 (Ar), 129.17 (Ar), 129.12 (Ar), 128.97 (Ar), 128.24 (Ar), 121.74 (Ar), 121.58 (Ar), 121.49 (Ar), 97.25 (C1′), 84.29, 81.76, 81.57 (C2), 81.48 (C4′), 79.63 (C2′), 77.99 (OCH_2_Ph), 77.25 (OCH_2_Ph), 76.82 (OCH_2_Ph), 74.75 (OCH_2_Ph), 74.41 (OCH_2_Ph), 74.07 (OCH_2_Ph), 73.71 (OCH_2_Ph), 73.02 (OCH_2_Ph), 72.73 (OCH_2_Ph), 72.41 (OCH_2_Ph), 71.20 (C5′), 68.54 (C7), and 68.31 (C6′). Mass spectrum (HRMS), *m/z* = 1544.6942 (M + Na)^+^; C_62_H_57_Br_7_O_10_ requires 1544.7113 (M + Na)^+^.

#### 3,4-Di(benzyloxy)-6-((benzyloxy)methyl)-5*-O-*(2′,3′,4′,6′-tetra*-O-*benzyl-α-D-glucopyranosyl)bicyclo[4.1.0]heptane-2-ol (22)

Under N_2_ atmosphere, diethyl zinc (0.431 ml, 0.51 mmol, 15 eq.) 15% by wt. in toluene solution was added to a cooled dry toluene (3 ml). The mixture was stirred at −15°C for 10 min and then dimethyl iodide (26 μl, 0.68 mmol, 20 eq.) was added dropwise to the reaction mixture. After 10 min, trifluoroacetic acid (4.3 µ l, 0.0578 mmol, 1.7 eq.), was added dropwise to the cooled solution following which the cooling bath was removed and the reaction mixture was stirred at room temperature for 5 min. To the resultant mixture, a solution of compound **21** (35 mg, 0.034 mmol) in dry toluene (3 ml) was added and the reaction mixture was stirred at room temperature for 15 h. The reaction was quenched by the addition of aq. HCl (10%) and then diluted with ethyl acetate (10 ml). After separation, the organic layer was washed with NaHCO_3_ solution (20 ml) and brine (20 ml), dried over anhydrous Na_2_SO_4_. The solvent was evaporated under reduced pressure and purification was performed by flash column chromatography on a silica gel (15% ethyl acetate:hexane) to afford product as colorless viscous liquid **22**: yield (0.22 mg, 62%); *R*_*f*_ = 0.38 (30% ethyl acetate:hexane); ^1^H NMR (600 MHz, CDCl_3_) δ 7.35–7.29 (m, 19H, Ar), 7.28–7.21 (m, 10H, Ar), 7.16 (m, 6H, Ar), 5.13 (d, *J =* 3.6 Hz, 1H, H-1′), 4.79 (ddd, *J =* 35.7, 18.5, 10.8 Hz, 4H, OCH_2_Ph), 4.61 (ddd, *J =* 21.5, 19.1, 9.7 Hz, 4H, OCH_2_Ph), 4.52–4.39 (m, 7H, OCH_2_Ph), 4.29 (d, *J =* 11.3 Hz, 1H, OCH_2_Ph), 4.05 (d, *J =* 10.3 Hz, 1H, H-7a), 4.00–3.95 (m, 1H, H-5′), 3.93 (t, *J =* 9.4 Hz, 1H, H-3′), 3.78 (dd, *J =* 6.8, 4.6 Hz, 1H), 3.74 (dd, *J =* 10.5, 3.2 Hz, 1H, H-6a′), 3.68 (t, *J =* 12 Hz, 1H, H-4′), 3.62 (dd, *J =* 6.8, 4.5 Hz, 1H), 3.54 (ddd, *J =* 9.8, 7.5, 5.1 Hz, 2H, H-2′, H-6b′), 2.68 (d, *J =* 10.3 Hz, 1H, H-7b), 2.47 (s, 1H), 1.57 (s, 2H), 1.40–1.34 (m, 2H), 0.93–0.88 (m, 1H), and 0.44 (q, *J =* 7.7 Hz, 1H). 13C NMR (151 MHz, CDCl3) δ 138.81 (Ar), 138.65 (Ar), 138.58 (Ar), 138.29 (Ar), 138.19 (Ar), 137.93 (Ar), 128.38 (Ar), 128.36 (Ar), 128.33 (Ar), 128.30 (Ar), 128.02 (Ar), 128.00 (Ar), 127.85 (Ar), 127.78 (Ar), 127.72 (Ar), 127.63 (Ar), 127.47 (Ar), 127.43 (Ar), 127.39, (Ar) 127.32 (Ar), 98.77 (C1′), 81.88 (C2), 80.06 (C2′), 78.92 (C3), 78.01 (C1), 77.83 (OCH_2_Ph), 76.82 (OCH_2_Ph), 76.62 (OCH_2_Ph), 76.10 (OCH_2_Ph), 75.38 (OCH_2_Ph), 75.14 (OCH_2_Ph), 73.53 (OCH_2_Ph), 73.16 (OCH_2_Ph), 72.81(C4′), 72.37 (OCH_2_Ph), 72.26 (OCH_2_Ph), 70.96 (C5′), 68.48 (C7), 64.40 (C6′), 29.73, 27.16, 24.36, and 9.43. Mass spectrum (HRMS), *m/z* = 1005.492 (M + 23)^+^; C_63_H_66_O_10_ requires 1005.455 (M + 23)^+^.

#### (1,3,4/2)-1,2-Di*-O-*(4-bromobenzyl)-4-C-[(4-bromobenzyloxy)methyl]-3*-O-*(2′,3′,4′,6′-tetra*-O-*(4-bromobenzyl)-α-D-glucopyranosyl)cyclohex-4-ene-1-(3,5-difluorophenoxy)-2,3,6-triol (23)

Sodium hydride (60% in hexanes) (0.38 mmol) was added to dry DMF in a flask at 0°C. To this mixture, a solution of compound **21′** (0.13 g, 0.085 mmol) in 2 ml of DMF was added dropwise. The resulting mixture was stirred for 30 min at the same temperature. Potassium benzoate (12.0 mg, 0.17 mmol) was added and stirred for 30 min. During this time, excess 1,3,5-trifluorobenzene (1.53 mmol) was added slowly. After 1 h the reaction was quenched by addition of NH_4_Cl. Following addition of brine (8 ml) organic layer was extracted with diethyl ether (10 ml), dried over anhydrous Na_2_SO_4_. The solvent was evaporated under reduced pressure and purification was performed by flash column chromatography on a silica gel to afford product as colorless viscous liquid **23**: yield 60% (0.09 g); silica gel TLC *R*_*f*_ = 0.68 (30% ethyl acetate:hexane). ^1^H NMR (600 MHz, CDCl_3_) δ 8.03 (d, *J =* 7.1 Hz, 1H, Ar), 7.46–7.33 (m, 21H, Ar), 7.16–6.90 (m, 9H, Ar), 6.46 (dd, *J =* 6.7, 5.1 Hz, 1H, H-1), 6.06 (d, *J =* 4.0 Hz, 1H, H-5), 5.38 (d, *J =* 3.6 Hz, 1H, H-1′), 4.88 (t, *J =* 3.7 Hz, 1H, H-2), 4.74 (t, *J =* 12.4 Hz, ^1^H, OCH_2_Ph), 4.66 (dd, *J =* 11.4, 3.5 Hz, 2H, OCH_2_Ph), 4.59–4.47 (m, 3H, OCH_2_Ph), 4.43 (dd, *J =* 12.2, 4.4 Hz, 2H, OCH_2_Ph), 4.42–4.30 (m, 3H, OCH_2_Ph), 4.31–4.20 (m, 2H, OCH_2_Ph), 3.95 (d, *J =* 12.7 Hz, 1H, OCH_2_Ph), 3.85 (dd, *J =* 18.5, 9.3 Hz, 1H, H-5′), 3.74 (dd, *J =* 8.2, 3.5 Hz, 1H, H-3′), 3.57 (t, *J =* 9.5 Hz, 1H, H-3), 3.49 (dd, *J =* 9.8, 3.6 Hz, 1H, H-4′), 3.44 (dd, *J =* 10.5, 3.1 Hz, 1H, H-2′), and 3.31 (d, *J =* 10.4 Hz, 2H, H-6a, H-6b). ^13^C NMR (151 MHz, CDCl_3_) δ 139.21 (Ar), 137.18 (Ar), 136.98 (Ar), 136.82 (Ar), 136.68 (Ar), 136.64 (Ar), 136.52 (Ar), 131.58 (Ar), 131.57 (Ar), 131.56 (Ar), 131.54 (Ar), 131.52 (Ar), 131.44 (Ar), 130.39 (Ar), 129.62 (Ar), 129.33 (Ar), 129.30 (Ar), 129.20 (Ar), 129.16 (Ar), 129.00 (Ar), 128.32 (Ar), 127.78 (Ar), 123.21 (Ar), 121.81 (Ar), 121.74 (Ar), 121.70 (Ar), 121.60 (Ar), 121.52 (Ar), 121.45 (Ar), 99.66 (C1), 99.47 (C1), 96.78 (C1′), 81.77 (C2), 79.60 (C3′), 77.67 (C4′), 77.24 (C2′), 77.03 (OCH_2_Ph), 76.82 (OCH_2_Ph), 74.51 (OCH_2_Ph), 74.12 (OCH_2_Ph), 72.91 (OCH_2_Ph), 72.74 (OCH_2_Ph), 72.15, 71.59 (OCH_2_Ph), 71.35 (OCH_2_Ph), 71.06 (C5′), 70.53 (C7), and 68.09 (C6′). Mass spectrum (HRMS), *m/z* = 1654.3391 (M + Na)^+^; C_68_H_59_Br_7_F_2_O_10_ requires 1654.3372 (M + Na)^+^.

#### 3,4-Di(benzyloxy)-6-((benzyloxy)methyl)-5*-O-*(2′,3′,4′,6′-tetra*-O-*benzyl-α-D-glucopyranosyl)bicyclo[4.1.0]heptane-2-(3,5-difluorophenoxy) (24)

After a suspension of sodium hydride (6.11 mg, 0.152 mmol, 4.5 eq.) in mineral oil (60%) was washed with hexane (2 X 5 ml), it was transferred in dry *N,N-*dimethylformamide (6 ml) into a flask. To this mixture a solution of compound **22** (30 mg, 0.030 mmol) in 2 ml of *N,N-*dimethylformamide was added dropwise. The resulting mixture was stirred for 30 min. Potassium benzoate (9.6 mg, 0.06 mmol, 2 eq.) was then added and stirring was continued for 30 min. During which time 1,3,5-trifluorobenzene was added slowly. After 2 h the reaction was quenched by addition of NH_4_Cl. Following addition of brine (8 ml) organic layer was extracted with diethyl ether (10 ml), dried over anhydrous Na_2_SO_4_. The solvent was evaporated under reduced pressure and purification was performed by flash column chromatography on a silica gel (7% ethyl acetate:hexane) to afford product as colorless viscous liquid **24**: yield 37 (0.17 mg, 52%); *R*_*f*_ = 0.8 (30% ethyl acetate:hexane). 1H NMR (600 MHz, CDCl_3_) δ 7.37–7.29 (m, 17H, Ar), 7.28–7.21 (m, 18H, Ar), 7.14–7.12 (m, 3H, Ar), 6.49 (dd, *J =* 9.0, 2.1 Hz, 1H), 6.43 (tt, *J =* 8.9, 2.1 Hz, 1H), 5.24 (d, *J =* 3.6 Hz, 1H, H-1), 4.94 (d, *J =* 10.9 Hz, 1H), 4.88–4.78 (m, 5H), 4.72 (d, *J =* 12.0 Hz, 1H), 4.67–4.62 (m, 1H), 4.62–4.38 (m, 8H), 4.07–3.99 (m, 2H, H-3, H-7a′), 3.98–3.93 (m, 1H, H-5), 3.83 (dd, *J =* 9.3, 6.4 Hz, 1H), 3.73–3.61 (m, 3H, H-4, H-6a), 3.54 (dd, *J =* 9.8, 3.6 Hz, 1H, H-2), 3.49 (dd, *J =* 10.4, 1.9 Hz, 1H, H-6b), 2.61 (d, *J =* 10.1 Hz, 1H, H-7b′), 1.40–1.34 (m, 2H), 1.09 (t, *J =* 5.7 Hz, 2H), 0.93–0.88 (m, 3H), and 0.48 (dd, *J =* 9.3, 5.4 Hz, 1H); ^13^C NMR (151 MHz, CDCl3) δ 138.95 (Ar), 138.77 (Ar), 138.43 (Ar), 138.26 (Ar), 137.84 (Ar), 128.41 (Ar), 128.38 (Ar), 128.29 (Ar), 128.24 (Ar), 128.20 (Ar), 128.07 (Ar), 128.01 (Ar), 127.88 (Ar), 127.79 (Ar), 127.75 (Ar), 127.72 (Ar), 127.64 (Ar), 127.59 (Ar), 127.55 (Ar), 127.50 (Ar), 127.45 (Ar), 127.39 (Ar), 127.23 (Ar), 99.56 (C1), 98.73 (C1′), 81.79 (C2), 80.52 (C3), 79.87 (C3′), 77.88 (C4), 77.24 (C4′), 77.03 (OCH_2_Ph), 76.82 (OCH_2_Ph), 75.60 (OCH_2_Ph), 75.54 (OCH_2_Ph), 75.22 (OCH_2_Ph), 73.95 (OCH_2_Ph), 73.54 (OCH_2_Ph), 72.98 (OCH_2_Ph), 72.74 (OCH_2_Ph), 72.58, 71.80 (C5′), 70.88 (C7), 68.44 (C6′), 29.72, 28.35, 22.75, 14.15 (CH), and 10.42 (CH2). Mass spectrum (HRMS), *m/z* = 1095.4851 (M + 23)^+^; C_69_H_68_F_2_O_10_ requires 1095.4814 (M + 23)^+^.

#### 2-(3,5-difluorophenoxy)-6-(hydroxymethyl)-5*-O-*(2′,3′,4′,6′-tetra-ol-*α-*D*-glucopyranosyl)* bicyclo[4.1.0]heptane-3,4-diol (11)

Dissolve compound **24** (10 mg, 0.009 mmol) in 3 ml of methanol add 5% Palladium on carbon (8 mg). The mixture was stirred for 4 h under hydrogen (1 atm.). The catalyst was filtered away through a plug of Celite^®^ 545 and washed with methanol (5ml). The combined filtrate and washings were concentrated to dryness afford **11** as a viscous syrup (3 mg, quantitative yield). ^1^H NMR (600 MHz, MeOD) δ 6.66 (dd, *J =* 9.2, 2.2 Hz, 2H, Ar), 6.52 (tt, *J =* 9.2, 2.3 Hz, 1H, Ar), 5.22 (d, *J =* 3.8 Hz, 1H, H-1′), 4.21–4.11 (m, 2H, H-7b), 3.84–3.65 (m, 2H, H-5′, H-3′), 3.56–3.52 (m, 1H, H-2′), 3.51–3.47 (m, 1H, H-6a′), 3.06 (q, *J =* 7.3 Hz, 1H, H-7a), 1.34–1.30 (m, 1H), 1.28–1.22 (m, 1H), 1.04–0.99 (m, 1H, CH_2_), and 0.49 (dd, 1H, *J =* 9.4, 5.4 Hz, CH_2_), ^13^C NMR (150 MHz, MeOD): δ 161.90 (Ar), 161.66 (Ar), 161.44 (Ar), 101.48, 99.17 (C1′), 98.97 (C1′), 73.80 (C2,C3′), 73.71 (C4′), 72.94 (C5′), 72.85, 71.14 (C7), 70.21 (C6′), 30.36, 21.26, 13.03 (CH_2_), and 8.77 (CH_2_). Mass spectrum (HRMS), *m/z* = 487.1401 (M + 23)^+^; C_20_H_26_F_2_O_10_ requires 487.1392 (M + 23)^+^.

#### (1D)-(1,3,4/2)-1,2-Di*-O-*(4-(*N*-methyl-*N*-phenyl))-4-C-[(4-(*N*-methyl-*N*-phenyl))methyl]-3*-O-*(2′,3′,4′,6′-tetra*-O-*(4-(*N*-methyl-*N*-phenyl))-α-D-glucopyranosyl)cyclohex-4-ene-1-(3,5-difluorophenoxy)-2,3,6-triol (25)

To a solution of compound **23** (0.09 g, 0.05 mmol) in toluene (4 ml), palladium (II) acetate (0.01 g, 0.05 mmol), XPhos (0.05 g, 0.10 mmol) and potassium phosphate tribasic (0.12 g, 0.57 mmol) were added at room temperature. *N*-Methylaniline was added dropwise to the mixture, the reaction was heated to 85°C and stirred at the same temperature for 5 days. After the completion of the reaction, the mixture was cooled to room temperature. The organic phase was washed with brine (2X 10 ml) and dried over anhydrous Na_2_SO_4_. The solvent was evaporated, and the product was isolated *via* flash column chromatography on a silica gel (20% ethyl acetate:hexane) to afford product as yellow viscous liquid **25**: yield 40% (0.04 g); silica gel TLC *R*_*f*_ = 0.70 (30% ethyl acetate:hexane). ^1^H NMR (600 MHz, CDCl_3_) δ 7.36–7.11 (m, 27H, Ar), 7.06–6.80 (m, 39H, Ar), 6.51–6.35 (m, 1H, H-1), 6.01 (d, *J =* 3.0 Hz, 1H, H-5), 5.46 (d, *J =* 3.4 Hz, 1H, H-1′), 4.84 (dd, *J =* 12.3, 7.4 Hz, 3H, H-2, OCH_2_Ph), 4.76 (dd, *J =* 10.3, 2.3 Hz, 3H, OCH_2_Ph), 4.72 (d, *J =* 11.2 Hz, 2H, OCH_2_Ph), 4.64 (dd, *J =* 15.7, 8.3 Hz, 3H, OCH_2_Ph), 4.58 (dd, *J =* 14.1, 12.0 Hz, 3H, OCH_2_Ph), 4.47–4.40 (m, 4H, OCH_2_Ph), 4.40–4.34 (m, 5H OCH_2_Ph, H-4), 4.33 (dd, *J =* 7.9, 4.6 Hz, 2H, H-5, H-3), 4.26 (d, *J =* 12.7 Hz, 1H, H-5′), 4.02–3.91 (m, 3H, H-6a′, H-6b′, H-7b), 3.81 (dd, *J =* 7.6, 3.7 Hz, 1H, H-2), 3.75–3.57 (m, 2H, H-2′, H-4′), and 3.30–3.17 (m, 21H, CH_3_). ^13^C NMR (151 MHz, CDCl_3_) δ 148.87 (Ar), 148.81 (Ar), 148.77 (Ar), 148.54 (Ar), 148.48 (Ar), 129.65 (Ar), 129.43 (Ar), 129.32 (Ar), 129.22 (Ar), 129.17 (Ar), 128.64 (Ar), 128.36 (Ar), 122.41 (Ar), 121.73 (Ar), 121.66 (Ar), 121.54 (Ar), 121.46 (Ar), 121.29 (Ar), 121.17 (Ar), 121.08 (Ar), 121.02 (Ar), 120.81 (Ar), 120.78 (Ar), 120.73 (Ar), 120.49 (Ar), 120.24 (Ar), 119.93 (Ar), 119.80 (Ar), 119.43 (Ar), 99.52 (C1′), 82.01 (C1), 77.25 (C3′), 77.04 (C3), 76.83 (C2), 75.40 (OCH_2_Ph), 74.77 (OCH_2_Ph), 73.59 (OCH_2_Ph), 73.27 (OCH_2_Ph), 72.79 (OCH_2_Ph), 72.21 (OCH_2_Ph), 71.74 (C7), 71.34 (C6′), 40.28, 40.25, 40.21, and 40.19 (CH_3_). Mass spectrum (HRMS), *m/z* = 1840.2834 (M + Na)^+^; C_62_H_57_Br_7_O_10_ requires 1839.9321 (M + Na)^+^.

#### 1-α-D-Glucopyranoside 4-(hydroxymethyl)-6-(4-(3,5 difluorophenoxy)) cyclohex-4-ene-2,3-triol (12)

Compound **25** (0.03 g, 0.018 mmol) was dissolved in anhydrous dichloromethane (2.00 ml). To this, 7 ml of 5%TFA/DCM solution was dropwise added at room temperature. Stir till the reaction becomes blue-green in color. Evaporate dichloromethane and dissolve compound in minimal water. The compound **12** was purified with reverse phase column chromatography using C18. The product was eluted with 1:1 Methanol/Water. Yield quantitative (2.00 mg); silica gel TLC *R*_*f*_ = 0.30 (10% methanol: dichloromethane). ^1^H NMR (600 MHz, MeOD) δ 6.66 (dd, *J =* 9.2, 2.1 Hz, 1H, Ar) 6.49 (s, 1H, Ar), 6.05 (dd, *J =* 5.0, 1.3 Hz, 1H, Ar), 5.43 (d, *J =* 18.5 Hz, 1H, H-7), 5.28 (d, *J =* 3.9 Hz, 1H, H-1′), 4.96–4.90 (m, 1H, H-1), 4.33–4.15 (m, 2H, H-3, H-4), 3.87 (dd, *J =* 11.7, 2.2 Hz, 1H, H-4′), 3.76 (dd, *J =* 9.6, 4.0 Hz, 1H, H-2), 3.65 (ddd, *J =* 20.8, 14.8, 7.0 Hz, 1H, H-6′), 3.46 (dd, *J =* 9.7, 3.9 Hz, 1H, H-2′), and 3.27 (d, *J =* 9.7 Hz, 1H, H-6′). ^13^C NMR (151 MHz, MeOD) δ 143.70 (Ar), 132.87 (Ar), 130.81 (Ar), 129.03 (Ar), 128.06 (Ar), 119.18 (C6), 100.13, 99.36 (C1′), 80.80 (C2, C2′), 73.78 (C3′), 73.39 (C4′), 72.60 (C1, C4), 71.91 (C3), 70.06 (C5′), 61.65 (C7), and 61.56 (C6’). Mass spectrum (HRMS), *m/z* = 473.3106 (M + Na)^+^; C_19_H_24_F_2_O_10_ requires 473.3913 (M + Na)^+^.

#### (2R,3R,4S,5R,6S)-3,4,5-tris(benzyloxy)-2-((benzyloxy)methyl)-6-(((1R,5R,6S)-5,6-bis(benzyloxy)-2-((benzyloxy)methyl)-4-bromocyclohex-2-en-1-yl)oxy)tetrahydro-2H-pyran (26)

Compound **18** (0.25 g, 0.26 mmol) was dissolved in dichloromethane (1.5 ml) at 0°C. To this solution was added anhydrous phosphorous tribromide (175.58 µl, 0.65 mmol). The reaction was left to stir at room temperature for 3 h. After completion of reaction, which was monitored by TLC, brine was added. The combined filtrates and washings were extracted with water (20 ml X 3) and organic layer was dried over anhydrous Na_2_SO_4_, evaporated under reduced pressure, and subjected to flash column chromatography on a silica gel with 1:7 ethyl acetate–hexane to give mixture of a and b products as yellowish brown viscous liquids **26a:26b** (1:1) total yield 55% (0.15 g); silica gel TLC *R*_*f*_ = 0.78 (30% ethyl acetate:hexane). **(26a)** ^1^H NMR (600 MHz, CDCl_3_) δ 7.35–7.24 (m, 23H, Ar), 7.20 –7.11 (m, 12H, Ar), 6.04 (s, 1H, H-5), 5.73 (d, *J =* 3.8 Hz, 1H, H-1′), 4.97 (d, *J =* 12.0 Hz, 1H, OCH_2_Ph), 4.88 (d, *J =* 10.9 Hz, 1H, OCH_2_Ph), 4.86–4.80 (m, 3H, OCH_2_Ph), 4.80–4.73 (m, 3H, OCH_2_Ph, H-1), 4.69 (s, 1H, OCH_2_Ph), 4.58 (d, *J =* 12.2 Hz, 1H, OCH_2_Ph), 4.49 (dd, *J =* 22.6, 11.2 Hz, 4H, OCH_2_Ph), 4.37 (d, *J =* 12.2 Hz, 1H, OCH_2_Ph), 4.32 (d, *J =* 12.9 Hz, 1H, H-6a′), 4.00–3.89 (m, 4H, H-6b′, H-3, H-3′, H-5′), 3.87 (dd, *J =* 9.8, 7.2 Hz, 1H, H-2), 3.72–3.65 (m, 1H, H-4′), 3.57 (td, *J =* 10.2, 3.3 Hz, 2H, H-6a, H-2), and 3.43 (dd, *J =* 10.6, 1.5 Hz, 1H, H-6b). ^13^C NMR (151 MHz, CDCl_3_) δ 138.65 (Ar), 138.42 (Ar), 138.39 (Ar), 137.92 (Ar), 137.88 (Ar), 137.64 (Ar), 136.02 (Ar), 128.38 (Ar), 128.36 (Ar), 128.34 (Ar), 128.31 (Ar), 128.26 (Ar), 128.08 (Ar), 127.96 (Ar), 127.85 (Ar), 127.75 (Ar), 127.73 (Ar), 127.67 (Ar), 127.64 (Ar), 127.60 (Ar), 127.55 (Ar), 127.21 (Ar), 126.23 (Ar), 97.40 (C1′), 85.00 (C3′), 84.54 (C2), 82.28 (C2′), 79.16 (C3), 77.82 (OCH_2_Ph), 77.25 (OCH_2_Ph), 77.04 (OCH_2_Ph), 76.83 (OCH_2_Ph), 75.89 (OCH_2_Ph), 75.52 (OCH_2_Ph), 74.97 (OCH_2_Ph), 74.11 (OCH_2_Ph), 73.80 (OCH_2_Ph), 73.60 (C4), 73.53 (C4′), 72.04 (OCH_2_Ph), 71.13 (C5′), 69.60 (C7), 68.15 (C6′), and 50.75 (C1). Mass spectrum (HRMS), *m/z* = 1054.3691 (M + Na)^+^; C_62_H_63_O_9_Br requires 1055.0672. **(26b)** ^1^H NMR (600 MHz, CDCl_3_) δ 7.39–7.20 (m, 33H, Ar), 7.18–7.12 (m, 2H, Ar), 6.11 (d, *J =* 5.5 Hz, 1H, H-5), 5.69 (d, *J =* 3.7 Hz, 1H, H-1′), 5.06 (d, *J =* 11.5 Hz, 1H, OCH_2_Ph), 4.92 (d, *J =* 10.9 Hz, ^1^H, OCH_2_Ph), 4.83 (d, *J =* 10.7 Hz, 3H, OCH_2_Ph, H-1), 4.71 (dd, *J =* 9.9, 5.8 Hz, 2H, OCH_2_Ph), 4.66 (dd, *J =* 25.9, 14.4 Hz, 2H, OCH_2_Ph), 4.60–4.53 (m, 2H, OCH_2_Ph), 4.45 (dt, *J =* 20.9, 11.3 Hz, 3H, OCH_2_Ph), 4.39–4.31 (m, 2H, OCH_2_Ph), 4.27 (d, *J =* 12.8 Hz, 1H, H-6b), 4.02–3.94 (m, 2H, H-6a, H, H-3), 3.89 (d, *J =* 9.9 Hz, 1H, H-5), 3.68 (t, *J =* 9.5 Hz, 1H, H-4) 3.61–3.51 (m, 2H, H-2, H-6a), and 3.43–3.37 (m, 1H, H-6b). ^13^C NMR (151 MHz, CDCl_3_) δ 138.74 (Ar), 138.55 (Ar), 138.48 (Ar), 138.09 (Ar), 137.99 (Ar), 137.89 (Ar), 137.52 (Ar), 128.45 (Ar), 128.36 (Ar), 128.34 (Ar), 128.30 (Ar), 128.27 (Ar), 128.07 (Ar), 128.01 (Ar), 127.89 (Ar), 127.86 (Ar), 127.82 (Ar), 127.73 (Ar), 127.66 (Ar), 127.63 (Ar), 127.60 (Ar), 127.56 (Ar), 127.53 (Ar), 127.19 (Ar), 126.85 (Ar), 125.15 (Ar), 96.58 (C1′), 82.05 (C3), 80.98 (C3′), 79.56 (OCH_2_Ph), 77.77 (C2′), 77.50 (C2), 77.26 (C4), 77.05 (OCH_2_Ph), 76.83 (OCH_2_Ph), 75.52 (OCH_2_Ph), 75.46 (C4′), 74.94 (OCH_2_Ph), 73.55 (OCH_2_Ph), 73.51 (OCH_2_Ph), 73.46 (OCH_2_Ph), 71.62 (C4), 71.14 (C5′), 70.09 (C7), 68.22 (C6’), and 48.57 (C1). Mass spectrum (ESI-MS), *m/z* = 1054.6 (M + Na)^+^; C_62_H_63_O_9_Br requires 1055.06.

#### (1S,4R,5S,6S)-5,6-bis(benzyloxy)-3-((benzyloxy)methyl)-*N*-cyclohexyl-4-(((2S,3R,4S,5R,6R)-3,4,5-tris(benzyloxy)-6-((benzyloxy)methyl)tetrahydro-2H-pyran-2-yl)oxy)cyclohex-2-en-1-amine (27)

Compounds **26a** and **26b** (0.15 g, 0.15 mmol) were dissolved in dichloromethane (1.0 ml) at room temperature. To this solution was added anhydrous cyclohexylamine (22.3 µl, 0.23 mmol). The reaction was left to stir at reflux for 5 days. After completion of reaction, which was monitored by TLC, the reaction mixture was cooled to room temperature and brine was added. The combined filtrates and washings were extracted with water (20 ml X 3) and organic layer was dried over anhydrous Na_2_SO_4_, evaporated under reduced pressure, and subjected to flash column chromatography on a silica gel with 1:1 ethyl acetate–hexane to give a yellowish brown viscous liquid **27**: yield 60% (0.09 g); silica gel TLC *R*_*f*_ = 0.3 (50% ethyl acetate:hexane). ^1^H NMR (600 MHz, CDCl_3_) δ 7.38–7.32 (m, 7H, Ar), 7.31 –7.16 (m, 28H, Ar), 5.92 (s, 1H), 5.22 (s, 1H, H-1′), 4.81 (d, *J =* 11.0 Hz, 1H, OCH_2_Ph), 4.76 (dd, *J =* 11.4, 3.0 Hz, 2H, OCH_2_Ph), 4.71–4.65 (m, 4H, OCH_2_Ph), 4.65–4.61 (m, 1H, OCH_2_Ph), 4.58 (dd, *J =* 11.9, 1.4 Hz, 3H, OCH_2_Ph), 4.46 (t, *J =* 9.0 Hz, 2H, OCH_2_Ph), 4.41 (d, *J =* 11.8 Hz, 1H, OCH_2_Ph), 4.34 (d, *J =* 13.7 Hz, 2H), 4.28 (d, *J =* 11.9 Hz, 1H), 4.22 (dd, *J =* 5.2, 2.4 Hz, 1H), 3.99–3.89 (m, 2H, H-2, H-3), 3.74–3.69 (m, 1H, H-5′), 3.69–3.57 (m, 3H, H-4′, H-3, H-2′), 3.46 (dd, *J =* 10.7, 1.8 Hz, 2H, H-6b, H-6a), 2.29 (s, 1H, C-Hex), 1.79 (d, *J =* 11.2 Hz, 2H, C-Hex), 1.65–1.58 (m, 2H, C-Hex) 1.25–1.13 (m, 3H, C-Hex), and 0.92–0.86 (m, 2H, C-Hex). ^13^C NMR (151 MHz, CDCl_3_) δ 138.79 (Ar), 138.59 (Ar), 138.52 (Ar), 138.08 (Ar), 138.02 (Ar), 128.51 (Ar), 128.40 (Ar), 128.34 (Ar), 128.33 (Ar), 128.27 (Ar), 128.24 (Ar), 128.21 (Ar), 128.18 (Ar), 128.14 (Ar), 127.99 (Ar), 127.81 (Ar), 127.73 (Ar), 127.64 (Ar), 127.62 (Ar), 127.48 (Ar), 127.40 (Ar), 127.30 (Ar), 98.01 (C1′), 81.98 (C2), 79.89 (C4), 77.89 (C2′), 77.25 (C3′), 77.04 (C4), 76.83 (OCH_2_Ph), 75.32 (OCH_2_Ph), 74.78 (OCH_2_Ph), 73.96 (OCH_2_Ph), 73.47 (OCH_2_Ph), 72.05 (OCH_2_Ph), 71.62 (OCH_2_Ph), 71.41 (OCH_2_Ph), 71.17 (C5′), 70.93 (C7), 68.22 (C6’), 52.99 (C1), 49.08 (C-Hex), 31.95 (C-Hex), 29.72 (C-Hex), 29.38 (C-Hex), 26.11 (C-Hex), 24.98 (C-Hex), 24.94 (C-Hex), and 22.72 (C-Hex). Mass spectrum (HRMS), *m/z* = 1072.2873 (M + Na)^+^; C_68_H_75_NO_9_ requires 1072.5262.

#### (2S,3R,4S,5S,6R)-2-(((1R,4S,5S,6R)-4-(cyclohexylamino)-5,6-dihydroxy-2-(hydroxymethyl)cyclohex-2-en-1-yl)oxy)-6-(hydroxymethyl)tetrahydro-2H-pyran-3,4,5-triol (13)

Ammonia was condensed into a solution of **27** (94.0 mg, 0.09 mmol) in tetrahydrofuran (3.0 ml) using a dry ice cooled cold finger apparatus. The solution was treated with sodium in small pieces, until a blue color in the solution persisted. After stirring for 10 min at −78°C , the mixture was treated with NH_4_Cl (70 mg), stirred at room temperature overnight and evaporated. The residue was extracted with methanol, filtered, and evaporated. The residue (25.0 mg) was redissolved in water and purified using reverse phase C18 column with a gradient of water:methanol to give **13** as a white solid: yield quantitative (32.0 mg). ^1^H NMR (600 MHz, MeOD) δ 5.85 (s, 1H, H-1), 5.32 (d, *J =* 3.0 Hz, 1H, H-1′), 4.21 (d, *J =* 4.0 Hz, 1H, H-3), 4.14 (s, 2H, H-4′, H-4), 4.10–4.05 (m, 1H, H-2), 3.99–3.95 (m, 1H, H-6a), 3.93 (s, 1H), 3.79 (d, *J =* 10.4 Hz, 1H, H-6b), 3.69 (dd, *J =* 16.2, 6.8 Hz, 1H, H-2′), 3.59– 3.54 (m, 1H), 3.54–3.50 (m, 1H, H-5′), 3.34 (d, *J =* 8.7 Hz, 1H, H-6a′), 3.11 (d, *J =* 7.3 Hz, 1H, H-6b;), 3.05 (s, 1H), 1.97 (d, *J =* 28.6 Hz, 3H, C-Hex), 1.77 (d, *J =* 34.4 Hz, 2H, C-Hex), 1.58 (d, *J =* 12.4 Hz, 1H, C-Hex), and 1.29–1.17 (m, 4H, C-Hex). ^13^C NMR (151 MHz, MeOD) δ 138.24 (C5), 119.43 (C6) 97.43 (C1′), 74.43 (C2), 72.81 (C3′), 72.76 (C2′), 71.12 (C3,C4′), 69.68 (C5′), 69.30 (C6′), 66.54 (C7), 61.85 (C1), 60.45, 55.24 (CN-Hex), 51.53 (C-Hex), 48.83 (C-Hex), 29.84 (C-Hex), 24.72 (C-Hex), and 24.18 (C-Hex). Mass spectrum (HRMS), *m/z* = 420.4788 (M + H)^+^; C_19_H_33_NO_9_ requires 420.4791.

Computational Methodology: The *Sco* GlgEI crystal structure was prepared using the Protein Preparation Wizard incorporated in Maestro (v.11.8) and Prime modules (Schrödinger, L.L.C. (2017)) to assign bond orders and charges and to remove water molecules and all heteroatoms. Restrained minimization was carried out to converge heavy atoms to RMSD of 0.3 Å using the OPLS3e force field. The ligands were drawn using ChemDraw (v21), imported into the workspace and prepared using the Ligprep module incorporated in Maestro (v11.8) *via* the OPLS3e force field (Kumar et al., 2020). Epik was used to generate possible states at target pH 7.0 ± 2.0 for accurate tautomer and ionization. The prepared ligands were docked into the active sites of the receptor (x = 25.27, y = −32.26, z = 277.11 *via* Glide docking in Maestro (v11.8) (Oyeneyin et al., 2021).

Protein purification: The protein purification was carried out according to the previously published method (Veleti et al., 2014b). In brief, the *Sco* GlgEI-V279S construct was used to transform T7 express *E. coli* cells. At 37°C, the largescale cultures were grown in LB media with a 264 mM concentration of carbenicillin. When O.D_600 nm_ reached 0.6, the temperature was lowered to 16°C, and 1 mM Isopropyl β-D-1-thiogalactopyranoside was added to cultures. After 16 h of induction, cells were harvested and resuspended in the lysis buffer (20 mM Tris pH 7.5, 500 mM NaCl, 10% glycerol, 0.5 mM imidazole, and 0.3 mM tris(2-carboxyethyl) phosphine (TECP)), and the resulting cell suspension was incubated on ice with lysozyme and DNase I. After 30 mins, the cell suspension was sonicated and centrifuged for 45 min. The clarified lysate was applied to a 5 ml metal affinity cobalt column, which was pre-equilibrated with lysis buffer. To remove unbound protein, 25 column volumes of lysis buffer were passed through the column and unbound protein was washed away. Finally, *Sco* GlgEI-V279S was eluted isocratically with elution buffer (20 mM Tris pH 7.5, 500 mM NaCl, 150 mM imidazole, and 0.3 mM TCEP) and dialyzed against 20 mM Tris pH 7.5, 150 mM NaCl, and 0.3 mM TCEP to get rid of excess salt and imidazole.

Inhibition studies: The inhibition studies on compound **13** were performed separately using a real-time assay and the EnzChek Phosphate Assay Kit. According to the literature, when the phosphate concentration of the reaction mixture is above 25 mM, GlgE catalyzes the reverse reaction of the production of M1P from glucans (Syson et al., 2011). In the real-time assay, the *Sco* GlgEI-V279S reverse reaction was coupled with α-glucosidase and performed according to the previously published method. In the presence of phosphate, *Sco* GlgEI-V279S catalyzes the hydrolysis of 2-chloro-4-nitrophenyl-D-maltotrioside (substrate) into M1P and 2-chloro-4-nitrophenyl glucose^12,13^. The 2-chloro-4-nitrophenyl glucose is further hydrolyzed into 2-chloro-4-nitrophenyl and glucose by α-glucosidase. The resulting 2-chloro-4-nitrophenyl exhibits an increased absorbance at 410 nm wavelength, and production of 2-chloro-4-nitrophenyl was measured continuously during the reaction. The reactions were carried out in a 96-well format, with a 50 µl reaction volume at 25°C for 40 min, and the velocity was measured at 410 nm wavelength using a Synergy H4 plate reader (Bio Tek). The reaction mixture consisted of 1.5 mM 2-chloro-4-nitrophenyl-D-maltotrioside, 20 mU α-glucosidase, 100 mM sodium phosphate, and 20 mM Tris pH 7.5. Finally, the reaction was initiated by adding 250 nM *Sco* GlgI-V279S to the reaction mixture. In the reaction, the compound **13** concentrations varied from 0 to 3000 µM. The positive and negative controls were lacking compound **13** and *Sco* GlgEI-V279S, respectively. The percent enzymatic activity was calculated by using the equation (V/V_0_) × 100, where V and V_0_ denote the rates of the inhibited and uninhibited enzyme (positive control), respectively.

The percent inhibition of α-glucosidase was calculated at each compound **13** concentration. In the reaction, 2-chloro-4-nitrophenyl-α-D-glucopyranoside was used as the substrate, and the production of 2-chloro-4-nitrophenyl was monitored throughout the reaction. The reaction mixture comprises 1.5 mM 2-chloro-4-nitrophenyl-α-D-glucopyranoside, 20 mM Tris pH 7.5, and each concentration of compound **13**. The reaction was initiated by adding 20 mU α-glucosidase. The compound **13** concentrations varied from 0 to 3000 μM. The percent enzymatic activity was calculated using the equation mentioned above. Finally, the total inhibition observed for *Sco* GlgEI-V279S and α-glucosidase was deducted using α-glucosidase inhibition at each concentration, and the actual GlgE inhibition was calculated. The results indicated that a 3000 μM concentration of compound **13** inhibits the activity of GlgE by 50%.

The EnzChek phosphate detection assay was performed according to the previously published method. The reaction mixture consisted of 20 mM Tris pH 7.5, 150 mM NaCl, 500 nM MESG, 0.25 U PNP, 0.5 mM glycogen (as maltose acceptor), and 250 µM M1P (Veleti et al., 2014a; Veleti et al., 2014b). The reaction was initiated by adding the 50 nM *Sco* GlgEI-V279S to a final concentration of 50 nM. All the reactions were carried out in 96 well formats in a triplicate manner, and a Synergy H4 plate reader (Bio Tek) was used to measure the absorbance at 360 nm. The 100 and 1000 µM concentrations of compound **13** were tested against *Sco* GlgEI-V279S, and neither of those concentrations exhibited inhibition of the *Sco* GlgE1-V279S activity.

Crystallization experiments: The *Sco* GlgEI-V279S/**13** mixture consisted of 8 mg/ml *Sco* GlgEI-V279S and 10 mM concentration of compound **13**. The crystallization experiments were performed using the hanging drop vapor diffusion method. Each drop consisted of 2 μl of *Sco* GlgEI V279S/compound mixture and 2 μl of the well solution of 0.2 mM sodium citrate pH 7 and 10% w/v PEG 3,350 (Lindenberger et al., 2015). The crystallization drops were equilibrated against 100 µl of well solution. To prepare for crystal cryoprotection, 4 μl of 50% w/v PEG 2000 was added to the drops containing the *Sco* GlgEI-V279S/ligand cocrystals and cryoprotected crystals were harvested and flash-cooled by immersing in liquid nitrogen.

X-ray diffraction experiments: The LS-CAT beamline at Advanced Photon Source of Argonne National Labs, IL, was used to perform X-ray diffraction experiments. The collected data were indexed, integrated, and scaled by using HKL 2000 (Otwinowski and Minor, 1997). The molecular replacement used the previously published *Sco* GlgEI-V279S in complex with a maltose-C-phosphonate structure (PDB ID:4U31), using Phaser (Mccoy et al., 2007) in PHENIX (Adams et al., 2010). The refinements were carried out using PHENIX. The visualization and manual refinements were performed using COOT (Emsley and Cowtan, 2004). Finally, MolProbity (Williams et al., 2018) in PHENIX was used for structure validation.

## Data Availability

The original contributions presented in the study are included in the article/Supplementary Material; further inquiries can be directed to the corresponding authors.
