# Supplementary material for: Synthesis of C7/C8-cyclitols and C7N-aminocyclitols from maltose and X-ray crystal structure of Streptomyces coelicolor GlgEI V279S in a complex with an amylostatin GXG–like derivative
Source: Front Chem. 2022 Sep 9;10:950433. doi: 10.3389/fchem.2022.950433 (PMC9501709; doi:10.3389/fchem.2022.950433)
Supplement: Supplementary file 1 [file DataSheet1.pdf]

Radhika Thanvi[‡1], Thilina D Jayasinghe[‡2], Sunayana Kapil[1], Babatunde Samuel Obadawo[1], Donald R Ronning[2*], and Steven J Sucheck[1*]

[1]Department of Chemistry and Biochemistry, The University of Toledo, Toledo, Ohio, USA

[2]Department of Pharmaceutical Sciences, University of Nebraska Medical Center, Omaha, NE, USA

[‡]These authors contributed equally to these work

*Correspondence: don.ronning@unmc.edu; steve.sucheck@utoledo.edu

Table of Contents:

Figure S1: 2D Ligand-receptor interaction diagram of GlgEI V279S with: a) **11**, b) **12**, c) **13**.	S6
Figure S2: The distance between Sco GlgEI V279S Glu 423 and compounds: a) **11**, b) **12**, c) **13**, in the Glide-docked models.	S7
Table S1: XP Docking score, XP Glide score and bond interactions of compounds **11**, **12** and **13** with the amino acid residues of GlgEI V279S	S8
Table S2: Crystallographic Data Table of the *Sco* GlgE1-V279S/13 structure	S9
^{1}H NMR (1,3,4/2)-1,2-Di-*O*-benzyl-4-C-[(benzyloxy)methyl]-4-*O*-acetyl-3-*O*-(2',3',4',6'-tetra-*O*-benzyl-α-D-glucopyranosyl)cyclohex-5-ene-1,2,3,4-tetrol (**20**)	S10
^{13}C NMR (1,3,4/2)-1,2-Di-*O*-benzyl-4-C-[(benzyloxy)methyl]-4-*O*-acetyl-3-*O*-(2',3',4',6'-tetra-*O*-benzyl-α-D-glucopyranosyl)cyclohex-5-ene-1,2,3,4-tetrol (**20**)	S11
^{1}H NMR (1,3,4/2)-1,2-Di-O-benzyl-4-C-[(benzyloxy)methyl]-3-O-(2',3',4',6'-tetra-O-benzyl-α-D-glucopyranosyl)cyclohex-4-ene-1,2,3,6-tetrol (**21**)	S12
^{13}C NMR (1,3,4/2)-1,2-Di-O-benzyl-4-C-[(benzyloxy)methyl]-3-O-(2',3',4',6'-tetra-O-benzyl-α-D-glucopyranosyl)cyclohex-4-ene-1,2,3,6-tetrol (**21**)	S13
^{1}H NMR 3,4-Di(benzyloxy)-6-((benzyloxy)methyl)-5-O-(2',3',4',6'-tetra-O-benzyl-α-D-glucopyranosyl)bicyclo[4.1.0]heptane-2-ol (**22**)	S14
^{13}C NMR 3,4-Di(benzyloxy)-6-((benzyloxy)methyl)-5-O-(2',3',4',6'-tetra-O-benzyl-α-D-glucopyranosyl)bicyclo[4.1.0]heptane-2-ol (**22**)	S15
^{1}H NMR 3,4-Di(benzyloxy)-6-((benzyloxy)methyl)-5-O-(2',3',4',6'-tetra-O-benzyl-α-D-glucopyranosyl)bicyclo[4.1.0]heptane-2-(3,5-difluorophenoxy) (**24**)	S16
^{13}C NMR 3,4-Di(benzyloxy)-6-((benzyloxy)methyl)-5-O-(2',3',4',6'-tetra-O-benzyl-α-D-glucopyranosyl)bicyclo[4.1.0]heptane-2-(3,5-difluorophenoxy) (**24**)	S17
^{1}H NMR 2-(3,5-difluorophenoxy)-6-(hydroxymethyl)-5-O-(2',3',4',6'-tetra-ol-*α-D-glucopyranosyl)* bicyclo[4.1.0]heptane-3,4-diol (**11**)	S18
^{13}C NMR 2-(3,5-difluorophenoxy)-6-(hydroxymethyl)-5-O-(2',3',4',6'-tetra-ol-*α-D-glucopyranosyl)* bicyclo[4.1.0]heptane-3,4-diol (**11**)	S19
^{1}H NMR 4-methylthiophenyl 2,3,6-Tri-O-(4-bromobenzyl)-4-O-(2',3',4',6'-tetra-O-(4-bromobenzyl)-α-D-glucopyranosyl)-β-D-glucopyranoside (**15'**)	S20
^{13}C NMR 4-methylthiophenyl 2,3,6-Tri-O-(4-bromobenzyl)-4-O-(2',3',4',6'-tetra-O-(4-bromobenzyl)-α-D-glucopyranosyl)-β-D-glucopyranoside (**15'**)	S21

^{1}H NMR 2,3,6-Tri-O-(4-bromobenzyl)-4-O-(2’,3’,4’,6’-tetra-O-(4-bromobenzyl)-α-D-glucopyranosyl)-α/β-D-glucopyranoside (**16’**)	S22
^{13}C NMR 2,3,6-Tri-O-(4-bromobenzyl)-4-O-(2’,3’,4’,6’-tetra-O-(4-bromobenzyl)-α-D-glucopyranosyl)-α/β-D-glucopyranoside (**16’**)	S23
^{1}H NMR 3,4,7-Tri-O-(4-bromobenyl)-5-O-(2’,3’,4’,6’-tetra-O-(4-bromobenzyl)-α-D-glucopyranosyl)-D-gluchept-1-enitol (**17’**)	S24
^{13}C NMR 3,4,7-Tri-O-(4-bromobenyl)-5-O-(2’,3’,4’,6’-tetra-O-(4-bromobenzyl)-α-D-glucopyranosyl)-D-gluchept-1-enitol (**17’**)	S25
^{1}H NMR 3,4,7-Tri-O-(4-bromobenzyl)-5-O-(2’,3’,4’,6’-tetra-O-(4-bromobenzyl)-α-D-glucopyranosyl)-D-gluchept-1-enone (**18’**)	S26
^{13}C NMR 3,4,7-Tri-O-(4-bromobenzyl)-5-O-(2’,3’,4’,6’-tetra-O-(4-bromobenzyl)-α-D-glucopyranosyl)-D-gluchept-1-enone (**18’**)	S27
^{1}H NMR 3,4,9-Tri-O-(4-bromobenzyl)-5-O-(2’,3’,4’,6’-tetra-O-(4-bromobenzyl)-α-D-glucopyranosyl)-D-gluco-octa-1,7-dienitol (**19A’**)	S28
^{13}C NMR 3,4,9-Tri-O-(4-bromobenzyl)-5-O-(2’,3’,4’,6’-tetra-O-(4-bromobenzyl)-α-D-glucopyranosyl)-D-gluco-octa-1,7-dienitol (**19A’**)	S29
^{1}H NMR 3,4,9-Tri-O-(4-bromobenzyl)-5-O-(2’,3’,4’,6’-tetra-O-(4-bromobenzyl)-α-D-glucopyranosyl)-L-ido-octa-1,7-dienitol (**19B’**)	S30
^{1}H NMR (1,3,4/2)-1,2-Di-O-(4-bromobenzyl)-4-C-[(4-bromobenzyloxy)methyl]-3-O-(2’,3’,4’,6’-tetra-O-(4-bromobenzyl)-α-D-glucopyranosyl)cyclohex-5-ene-1,2,3,4-tetrol (**10’**)	S31
^{13}C NMR (1,3,4/2)-1,2-Di-O-(4-bromobenzyl)-4-C-[(4-bromobenzyloxy)methyl]-3-O-(2’,3’,4’,6’-tetra-O-(4-bromobenzyl)-α-D-glucopyranosyl)cyclohex-5-ene-1,2,3,4-tetrol (**10’**)	S32
^{1}H NMR (1,3,4/2)-1,2-Di-O-(4-bromobenzyl)-4-C-[(4-bromobenzyloxy)methyl]-4-O-acetyl-3-O-(2’,3’,4’,6’-tetra-O-(4-bromobenzyl)-α-D-glucopyranosyl)cyclohex-5-ene-1,2,3,4-tetrol (**20’**)	S33
^{13}C NMR (1,3,4/2)-1,2-Di-O-(4-bromobenzyl)-4-C-[(4-bromobenzyloxy)methyl]-4-O-acetyl-3-O-(2’,3’,4’,6’-tetra-O-(4-bromobenzyl)-α-D-glucopyranosyl)cyclohex-5-ene-1,2,3,4-tetrol (**20’**)	S34
^{1}H NMR (1,3,4/2)-1,2-Di-O-(4-bromobenzyl)-4-C-[(4-bromobenzyloxy)methyl]-3-O-(2’,3’,4’,6’-tetra-O-(4-bromobenzyl)-α-D-glucopyranosyl)cyclohex-4-ene-1,2,3,6-tetrol (**21’**)	S35

^{13}C NMR (1,3,4/2)-1,2-Di-O-(4-bromobenzyl)-4-C-[(4-bromobenzyloxy)methyl]-3-O-(2’,3’,4’,6’-tetra-O-(4-bromobenzyl)-α-D-glucopyranosyl)cyclohex-4-ene-1,2,3,6-tetrol (**21’**)	S36
^{1}H NMR 3,4-Di(4-bromobenzyloxy)-6-((4-bromobenzyloxy)methyl)-5-O-(2’,3’,4’,6’-tetra-O-4-bromobenzyl-α-D-glucopyranosyl)- 2-(3,5-difluorophenoxy) (**23**)	S37
^{13}C NMR 3,4-Di(4-bromobenzyloxy)-6-((4-bromobenzyloxy)methyl)-5-O-(2’,3’,4’,6’-tetra-O-4-bromobenzyl-α-D-glucopyranosyl)- 2-(3,5-difluorophenoxy) (**23**)	S38
^{1}H NMR 3,4-Di(4-(N-methyl, N-phenyl)benzyloxy)-6-((4-(N-methyl, N-phenyl)benzyloxy)methyl)-5-O-(2’,3’,4’,6’-tetra-O-4-(N-methyl, N-phenyl)benzyl-α-D-glucopyranosyl)- 2-(3,5-difluorophenoxy) (**25**)	S39
^{13}C NMR 3,4-Di(4-(N-methyl, N-phenyl)benzyloxy)-6-((4-(N-methyl, N-phenyl)benzyloxy)methyl)-5-O-(2’,3’,4’,6’-tetra-O-4-(N-methyl, N-phenyl)benzyl-α-D-glucopyranosyl)- 2-(3,5-difluorophenoxy) (**25**)	S40
^{1}H NMR 1-α-D-Glucopyranoside 4-(hydroxymethyl)-6- (4-(3,5 difluorophenoxy)) cyclohex-4-ene-2,3-triol (**12**)	S41
^{13}C NMR 1-α-D-Glucopyranoside 4-(hydroxymethyl)-6- (4-(3,5 difluorophenoxy)) cyclohex-4-ene-2,3-triol (**12**)	S42
^{1}H NMR (2R,3R,4S,5R,6S)-3,4,5-tris(benzyloxy)-2-((benzyloxy)methyl)-6-(((1R,5R,6S)-5,6-bis(benzyloxy)-2-((benzyloxy)methyl)-4-bromocyclohex-2-en-1-yl)oxy)tetrahydro-2H-pyran (**26a**)	S43
^{13}C NMR (2R,3R,4S,5R,6S)-3,4,5-tris(benzyloxy)-2-((benzyloxy)methyl)-6-(((1R,5R,6S)-5,6-bis(benzyloxy)-2-((benzyloxy)methyl)-4-bromocyclohex-2-en-1-yl)oxy)tetrahydro-2H-pyran (**26a**)	S44
^{1}H NMR (2R,3R,4S,5R,6S)-3,4,5-tris(benzyloxy)-2-((benzyloxy)methyl)-6-(((1R,5R,6S)-5,6-bis(benzyloxy)-2-((benzyloxy)methyl)-4-bromocyclohex-2-en-1-yl)oxy)tetrahydro-2H-pyran (**26b**)	S45
^{13}C NMR (2R,3R,4S,5R,6S)-3,4,5-tris(benzyloxy)-2-((benzyloxy)methyl)-6-(((1R,5R,6S)-5,6-bis(benzyloxy)-2-((benzyloxy)methyl)-4-bromocyclohex-2-en-1-yl)oxy)tetrahydro-2H-pyran (**26b**)	S46
^{1}H NMR (1S,4R,5S,6S)-5,6-bis(benzyloxy)-3-((benzyloxy)methyl)-N-cyclohexyl-4-(((2S,3R,4S,5R,6R)-3,4,5-tris(benzyloxy)-6-((benzyloxy)methyl)tetrahydro-2H-pyran-2-yl)oxy)cyclohex-2-en-1-amine (**27**)	S47

^{13}C NMR (1S,4R,5S,6S)-5,6-bis(benzyloxy)-3-((benzyloxy)methyl)-N-cyclohexyl-4-(((2S,3R,4S,5R,6R)-3,4,5-tris(benzyloxy)-6-((benzyloxy)methyl)tetrahydro-2H-pyran-2-yl)oxy)cyclohex-2-en-1-amine (**27**)	S48
^{1}H NMR (2S,3R,4S,5S,6R)-2-(((1R,4S,5S,6R)-4-(cyclohexylamino)-5,6-dihydroxy-2-(hydroxymethyl)cyclohex-2-en-1-yl)oxy)-6-(hydroxymethyl)tetrahydro-2H-pyran-3,4,5-triol (**13**)	S49
^{13}C NMR (2S,3R,4S,5S,6R)-2-(((1R,4S,5S,6R)-4-(cyclohexylamino)-5,6-dihydroxy-2-(hydroxymethyl)cyclohex-2-en-1-yl)oxy)-6-(hydroxymethyl)tetrahydro-2H-pyran-3,4,5-triol (**13**)	S50
References	S51

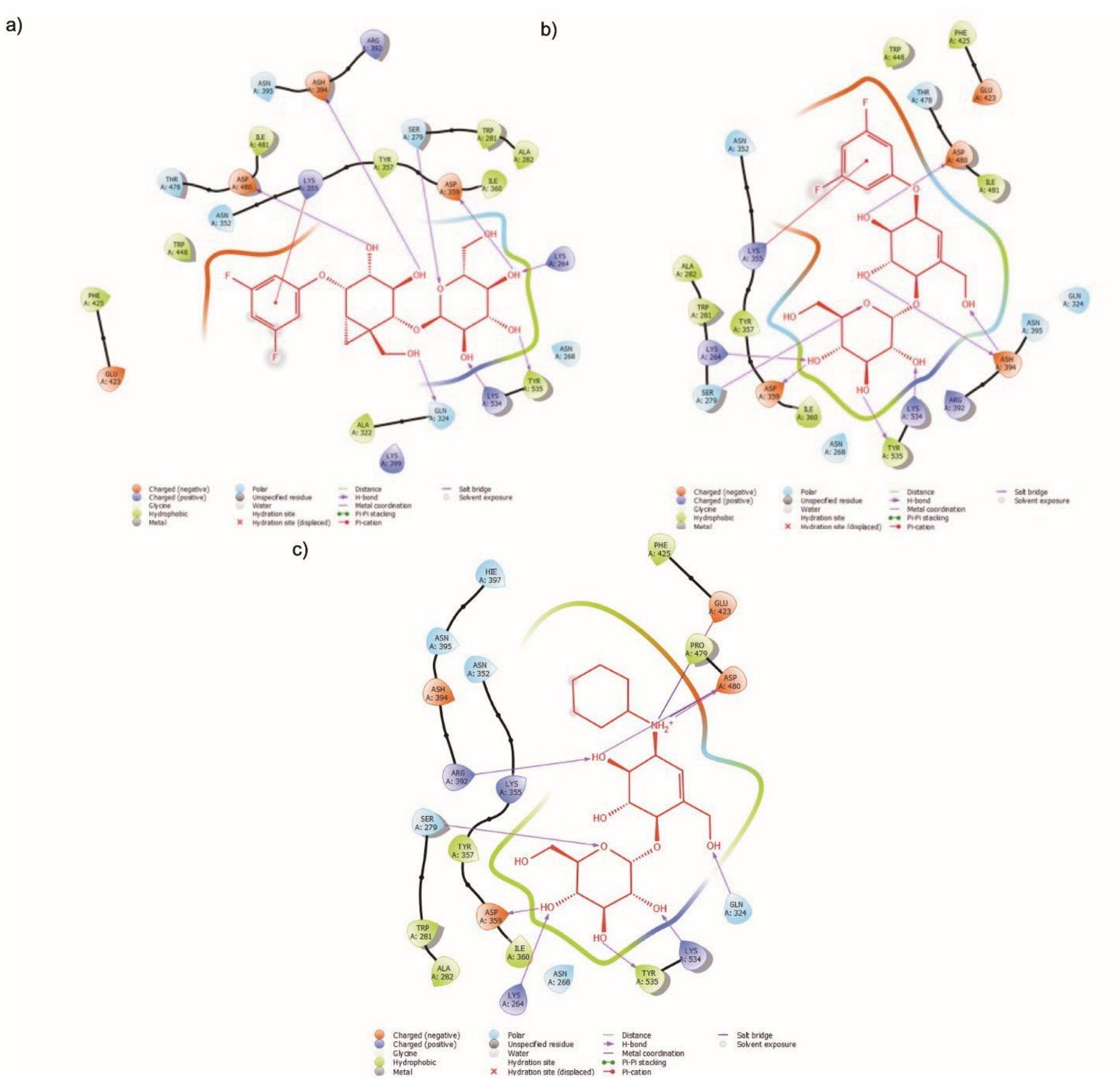

Figure S1: 2D Ligand-receptor interaction diagram of *Sco* GlgEI V279S with a) **11** b) **12** c) **13**

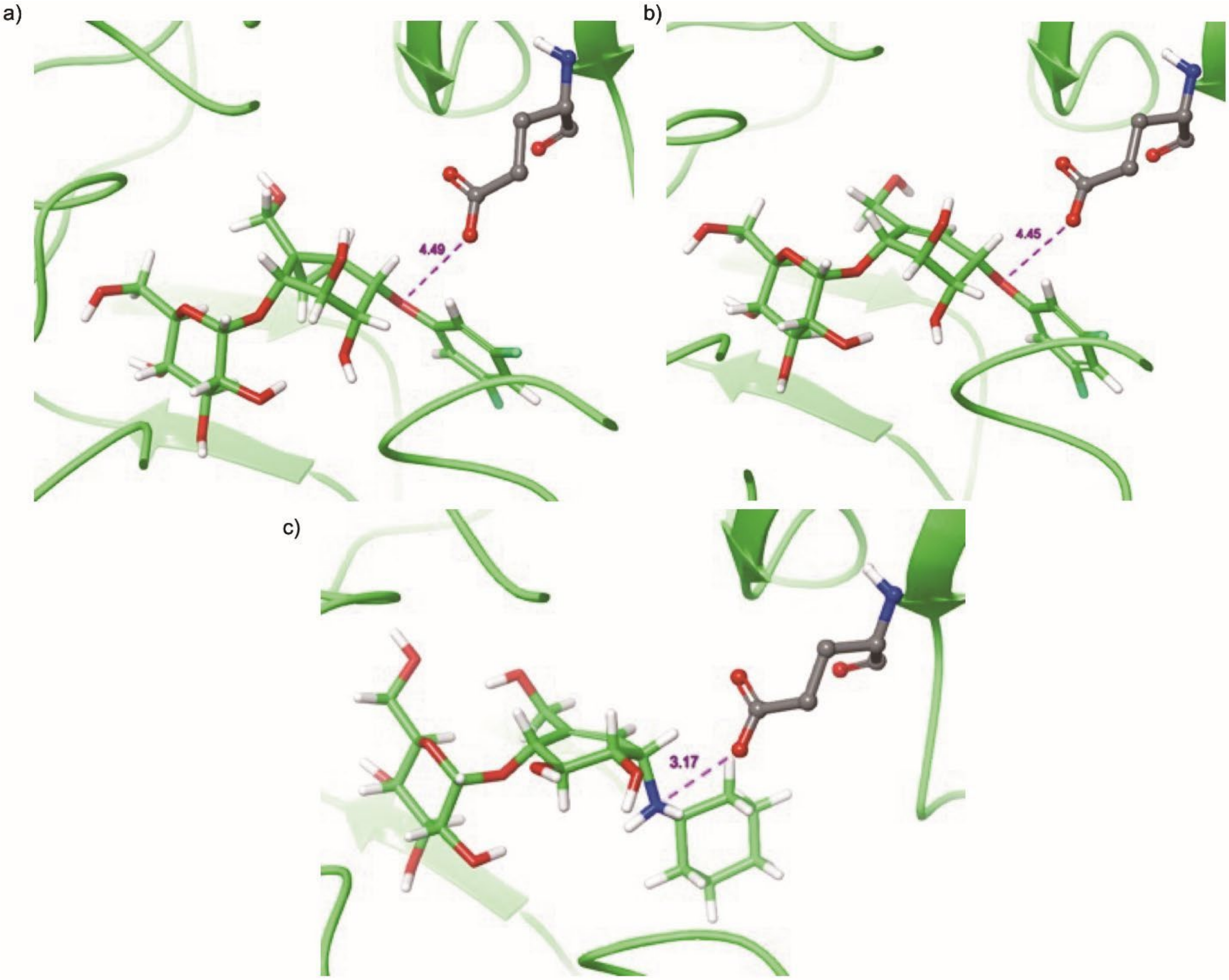

Figure S2: The distance between *Sco* GlgEI V279S Glu 423 and compounds: a) **11**, b) **12**, c) **13**, in the Glide-docked models.

Table S1: XP Docking score, XP Glide score and bond interactions of compounds **11**, **12** and **13** with the amino acid residues of GlgEI V279S

Molecules	XP Docking Score	XP Glide Score	Amino acid residues	Bond interaction
**11**	-11.908	-11.908	GLN 324<br>LYS 534<br>TYR 535<br>LYS 264<br>ASP 359<br>SER 279<br>ASH 394<br>ASP 480<br>LYS 355	Hydrogen bond<br>Hydrogen bond<br>Hydrogen bond<br>Hydrogen bond<br>Hydrogen bond<br>Hydrogen bond<br>Hydrogen bond<br>Hydrogen bond<br>Salt bridge
**12**	-11.375	-11.375	LYS 264<br>SER 279<br>ASP 359<br>TYR 535<br>LYS 534<br>ASH 394<br>ASP 480<br>LYS 355	Hydrogen bond<br>Hydrogen bond<br>Hydrogen bond<br>Hydrogen bond<br>Hydrogen bond<br>Hydrogen bond<br>Hydrogen bond<br>Salt bridge
**13**	-12.363	-12.377	SER 279<br>ASP 359<br>LYS 264<br>LYS 534<br>TYR 535<br>ARG 392<br>ASP 480<br>GLN 324<br>GLU 423	Hydrogen bond<br>Hydrogen bond<br>Hydrogen bond<br>Hydrogen bond<br>Hydrogen bond<br>Hydrogen bond<br>Hydrogen bond, Salt bridge<br>Hydrogen bond<br>Salt bridge

Compound **13** showed H-bond interaction from the hydroxyl groups to Ser 279, Asp 359, Lys 264, Lys 534, Tyr 535, Arg 392, Asp 480, Gln 324 amino acid residues. Despite these interactions, the cyclohexyl ring itself did not show any interaction to the protein in the Glide-Docked model. However, modification of the cyclohexylamine moiety is expected for additional interactions with the protein. In a previous work published by our group, Glu 423 was seen to interact with the nitrogen at the anomeric position (Si et al., 2021). In this docking study, Compound **11** (4.49 Å) and **12** (4.45 Å) did not record any Glu 423 interaction with the pseudoanomeric oxygen. Compound **13** pseudoanomeric nitrogen was found to interact with Glu 423 at a distance of 3.17 Å among other bond interaction at the anomeric position (Figure S2). Owing to the crucial role of Glu 423 in the catalytic steps and stabilization of the complex, interaction of Glu 423 at the anomeric position could also help in the search for GlgE inhibitor with better activity.

Table S2: Crystallographic Data Table of the *Sco* GlgE1-V279S/**13** structure

Data collection	
Wavelength (Å)	0.98
Space group	P 41 21 2
Unit cell dimensions	
a (Å)	112.438
b (Å)	112.438
c (Å)	310.411
α	90°
β	90°
ϒ	90°
Resolution (Å)	49.4 - 2.64
R_{pim}	0.112(0.856)
R_{merge}	0.316(2.483)
$CC_{1/2}$	0.997(0.791)
$I/\sigma I$	6.1(0.69)
Completeness (%)	96.28 (99.91)
Refinement	
Total no. of reflections	5272398
No. of unique reflections	51907 (5263)
R_{work}/R_{free}	0.1926/0.2578
No. of atoms	
Protein	10348
Ligand/ion	72
Water	253
B-factors (Å^2)	
Protein	57.79
Ligand/ion	70.03
Water	52.08
R.m.s deviations	
Bond length (Å)	0.009
Bond angels (°)	1.16
Ramachandran	
Favored (%)	97.45
Outliers (%)	0.00

^{1}H NMR (1,3,4/2)-1,2-Di-*O*-benzyl-4-C-[(benzyloxy)methyl]-4-*O*-acetyl-3-*O*-(2’,3’,4’,6’-tetra-*O*-benzyl-α-D-glucopyranosyl)cyclohex-5-ene-1,2,3,4-tetrol (**20**)

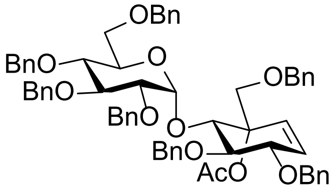

20

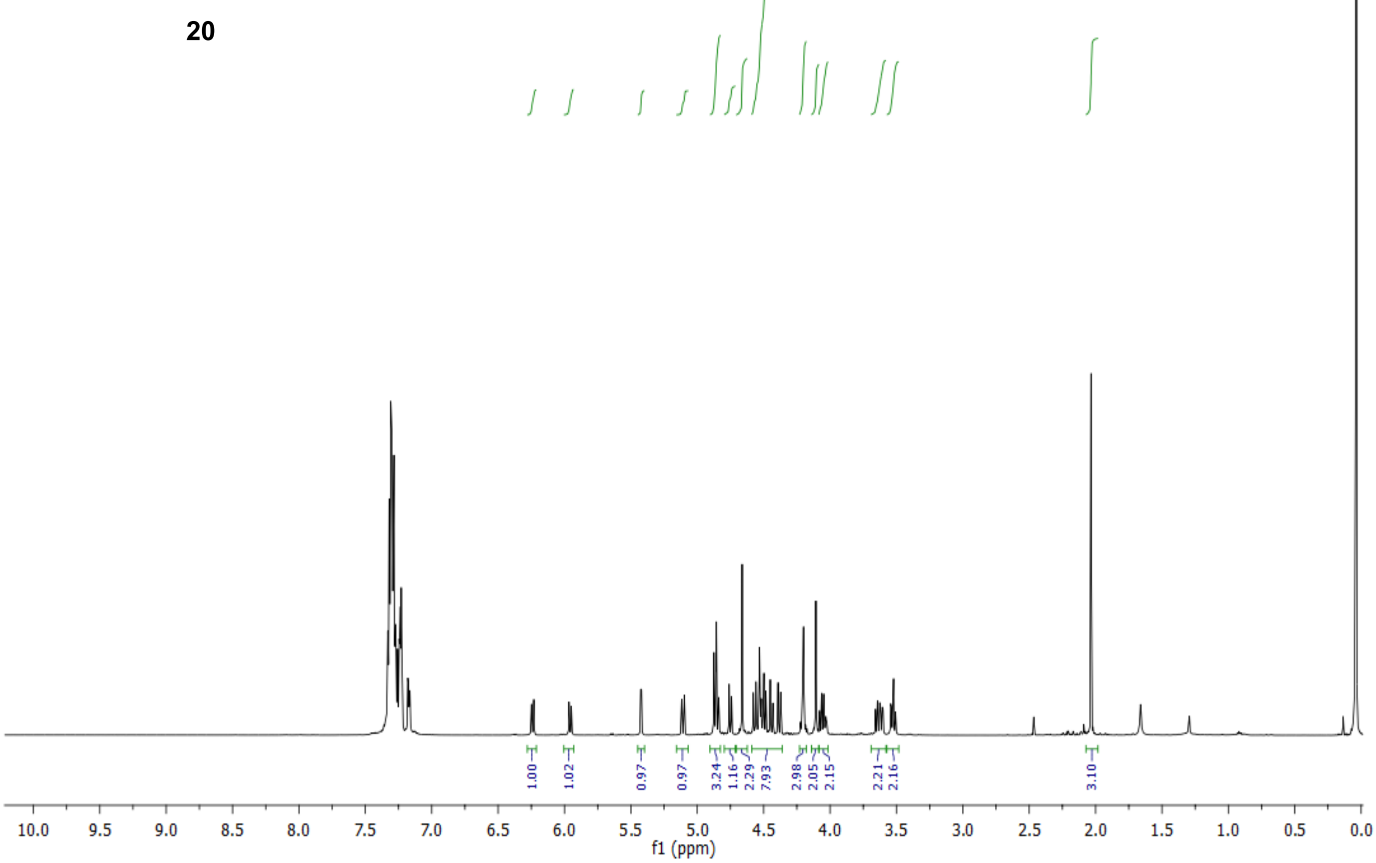

^{13}C NMR (1,3,4/2)-1,2-Di-*O*-benzyl-4-C-[(benzyloxy)methyl]-4-*O*-acetyl-3-O-(2’,3’,4’,6’-tetra-*O*-benzyl-α-D-glucopyranosyl)cyclohex-5-ene-1,2,3,4-tetrol (**20**)

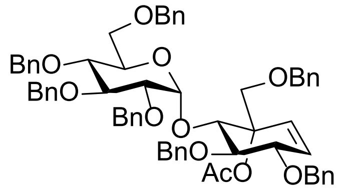

20

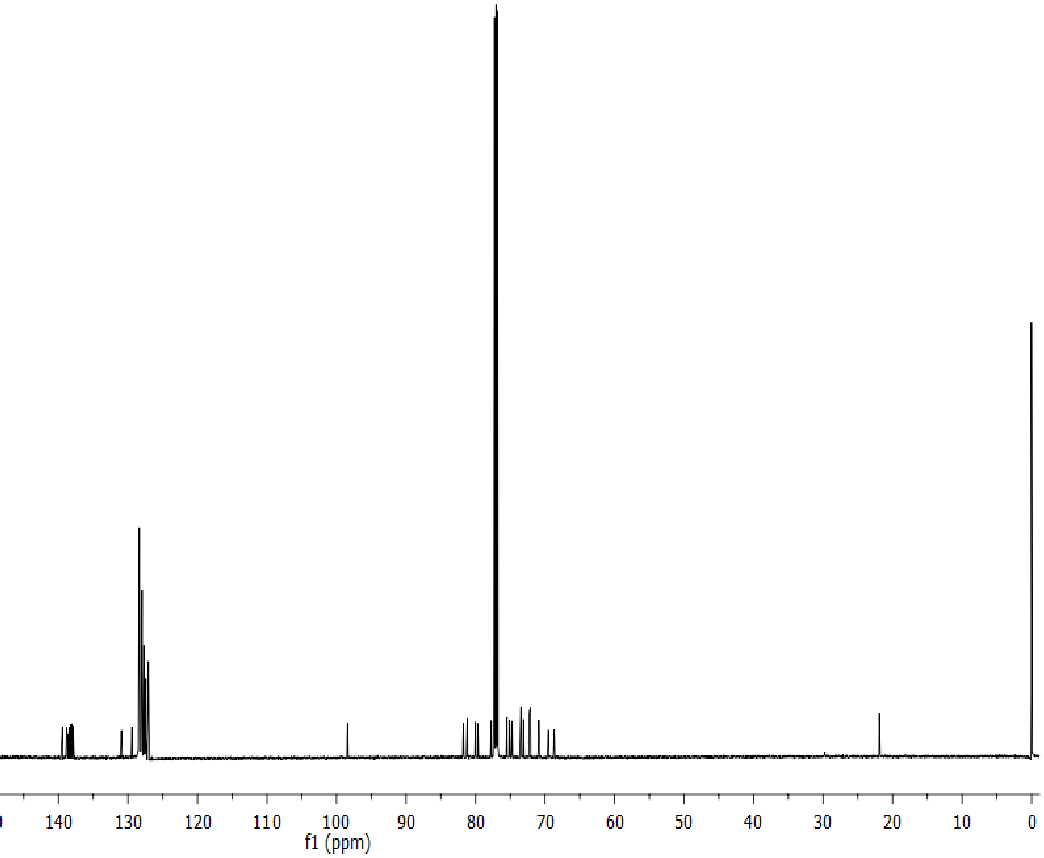

¹H NMR (1,3,4/2)-1,2-Di-*O*-benzyl-4-C-[(benzyloxy)methyl]-3-*O*-(2’,3’,4’,6’-tetra-*O*-benzyl-α-D-glucopyranosyl)cyclohex-4-ene-1,2,3,6-tetrol (**21**)

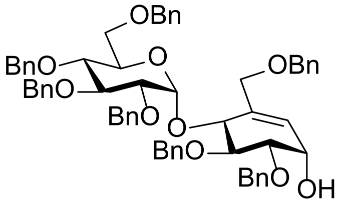

21

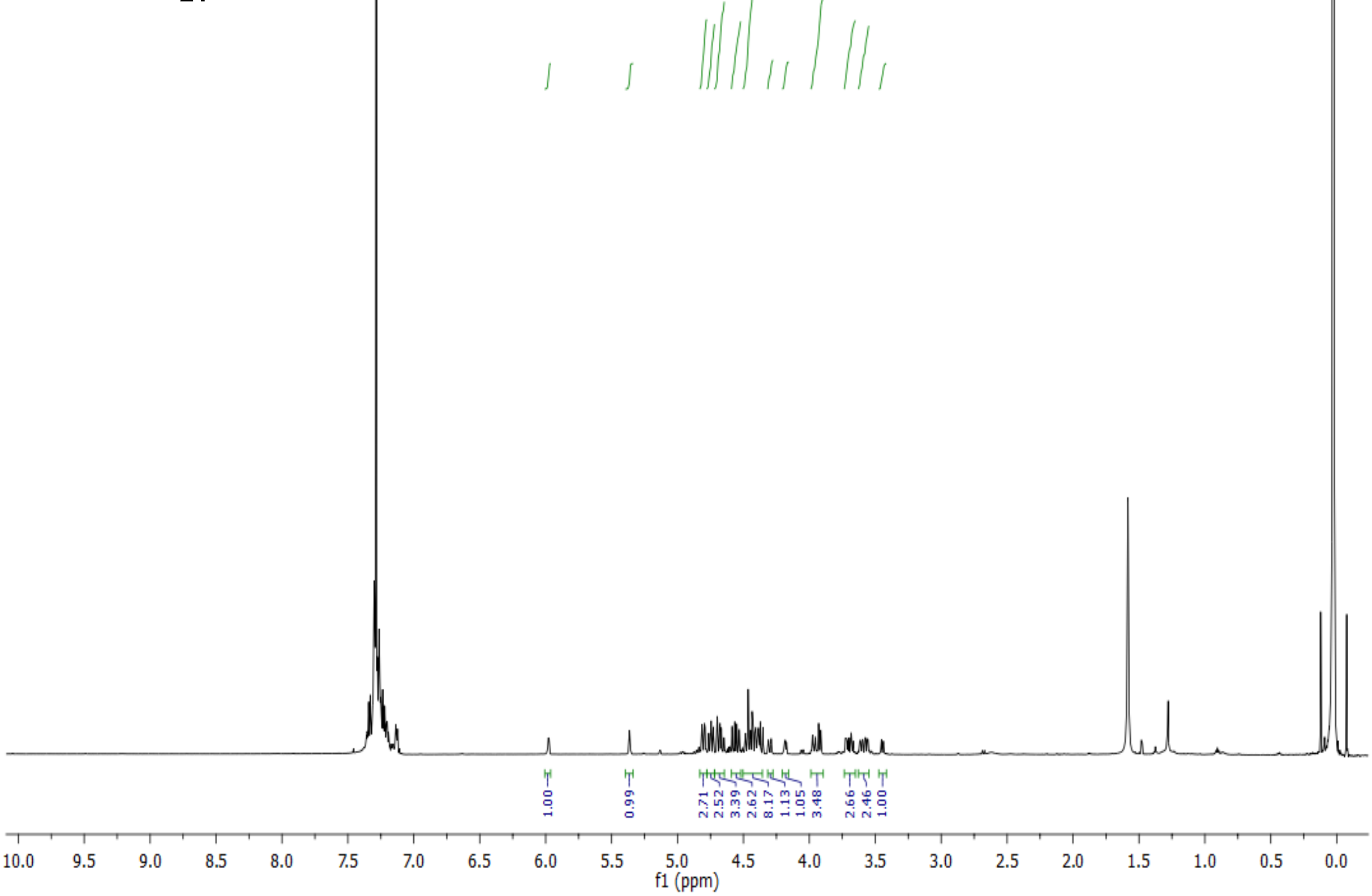

^{13}C NMR (1,3,4/2)-1,2-Di-O-benzyl-4-C-[(benzyloxy)methyl]-3-O-(2',3',4',6'-tetra-O-benzyl-α-D-glucopyranosyl)cyclohex-4-ene-1,2,3,6-tetrol (**21**)

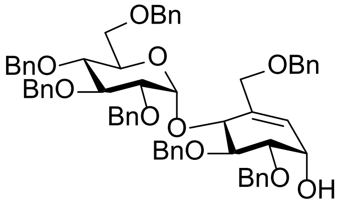

21

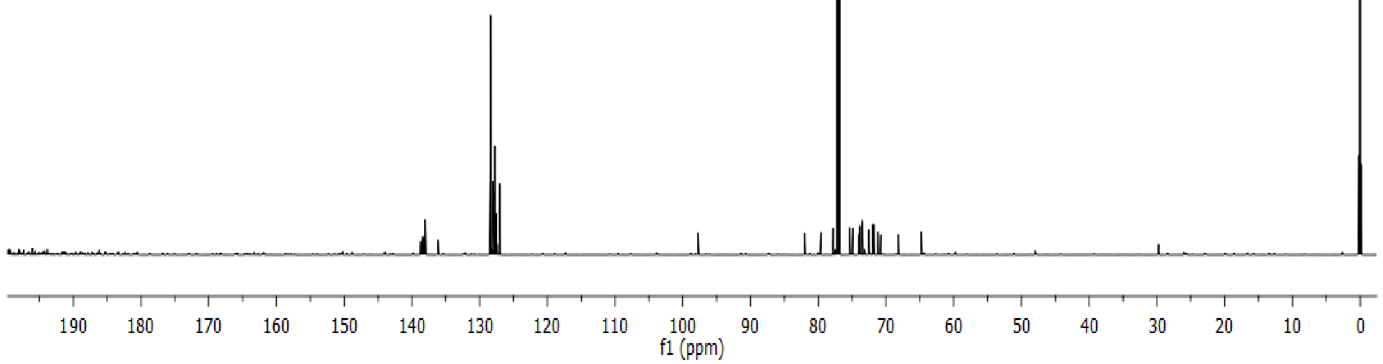

^1H NMR 3,4-Di(benzyloxy)-6-((benzyloxy)methyl)-5-O-(2',3',4',6'-tetra-O-benzyl-α-D-glucopyranosyl)bicyclo[4.1.0]heptane-2-ol (**22**)

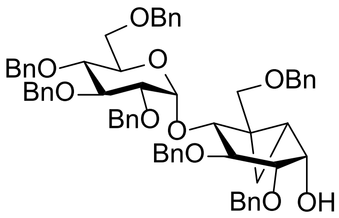

22

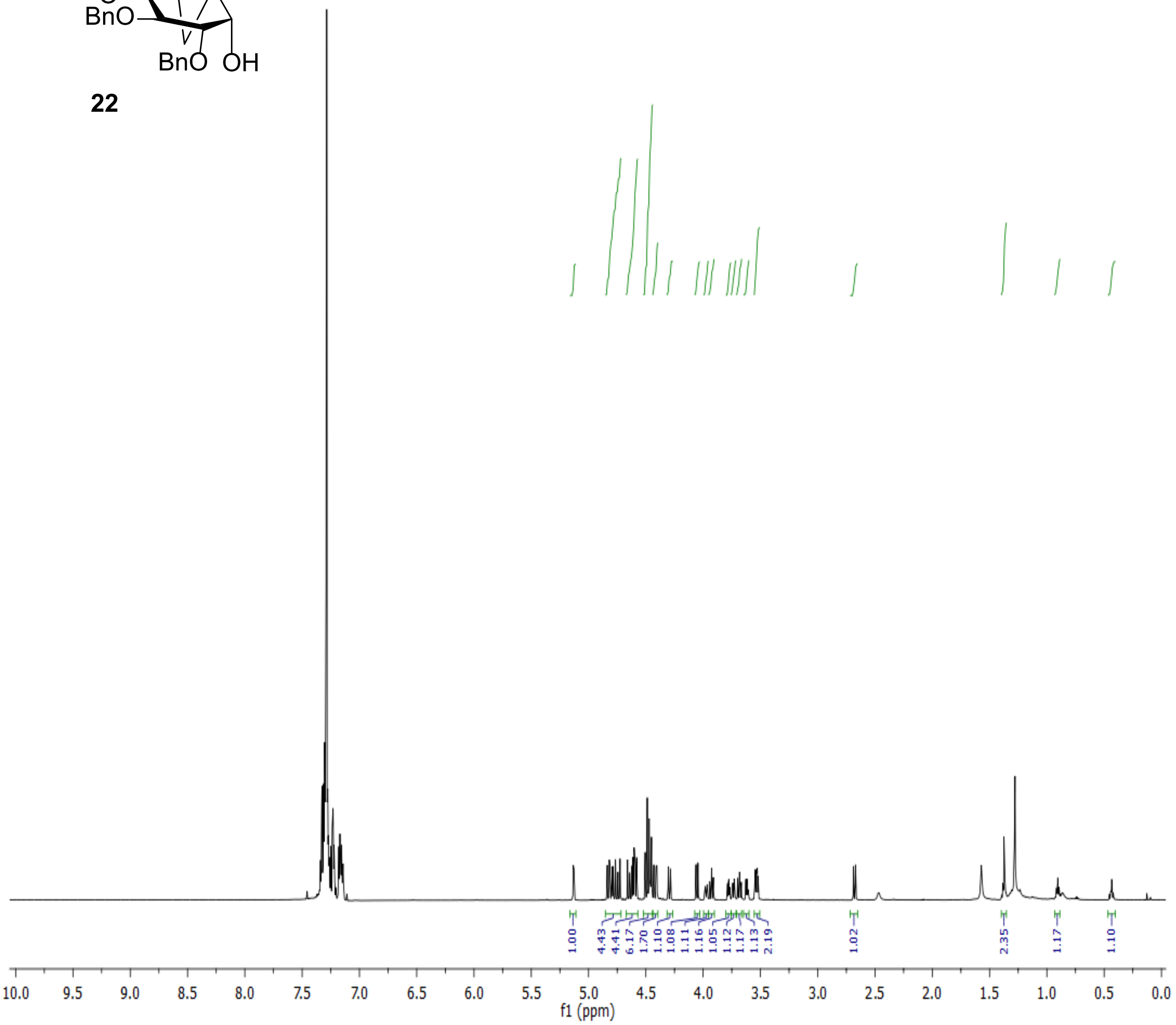

^{13}C NMR 3,4-Di(benzyloxy)-6-((benzyloxy)methyl)-5-O-(2’,3’,4’,6’-tetra-O-benzyl-α-D-glucopyranosyl)bicyclo[4.1.0]heptane-2-ol (**22**)

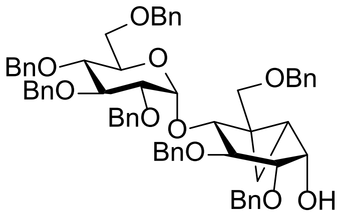

22

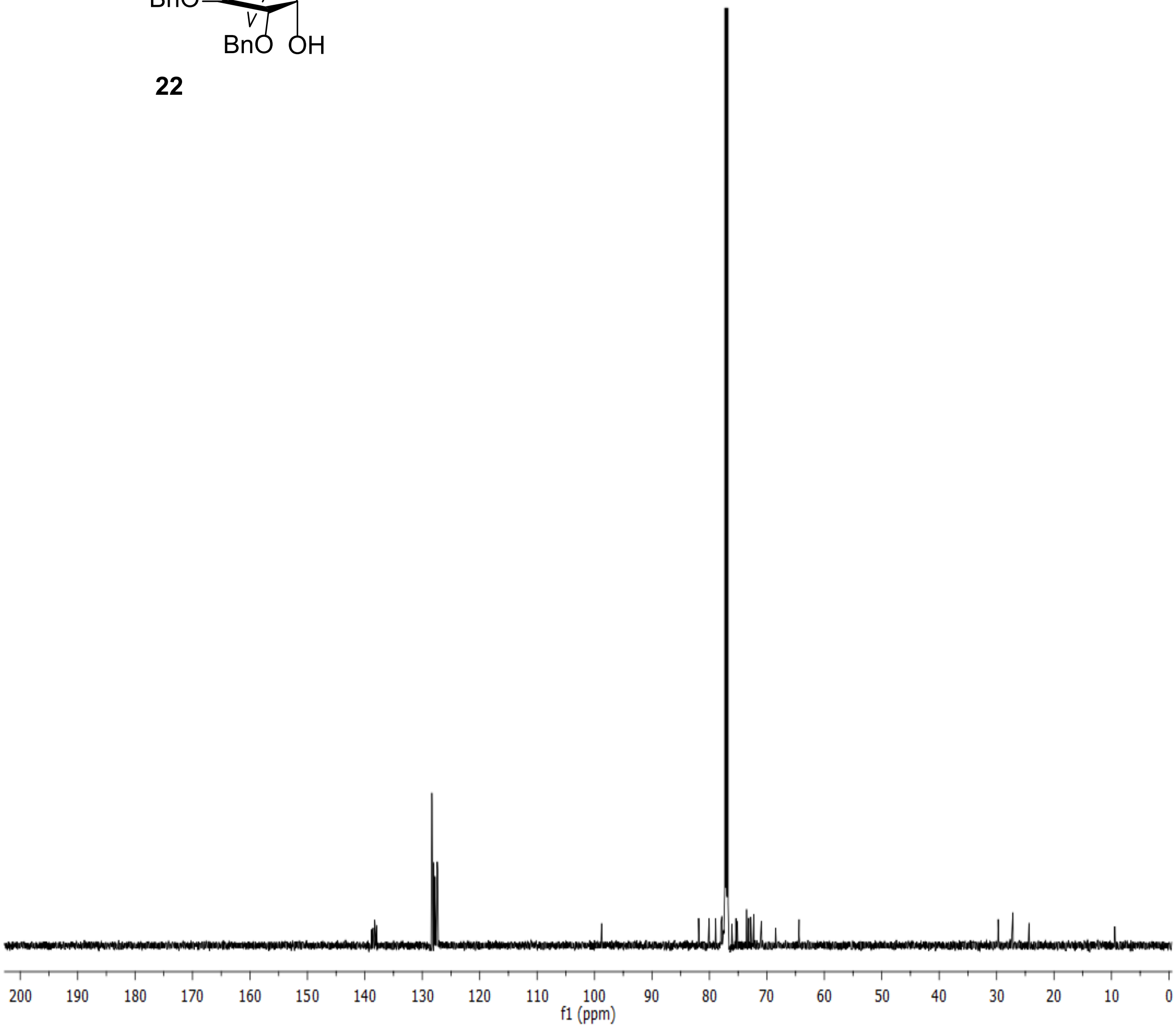

^{1}H NMR 3,4-Di(benzyloxy)-6-((benzyloxy)methyl)-5-O-(2’,3’,4’,6’-tetra-O-benzyl-α-D-glucopyranosyl)bicyclo[4.1.0]heptane-2-(3,5-difluorophenoxy) (**24**)

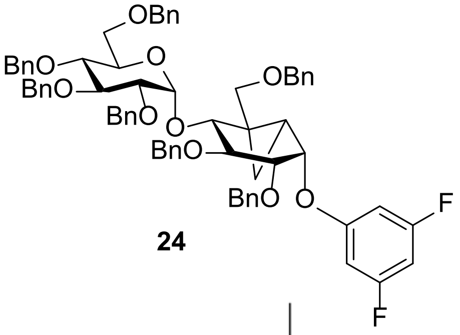

24

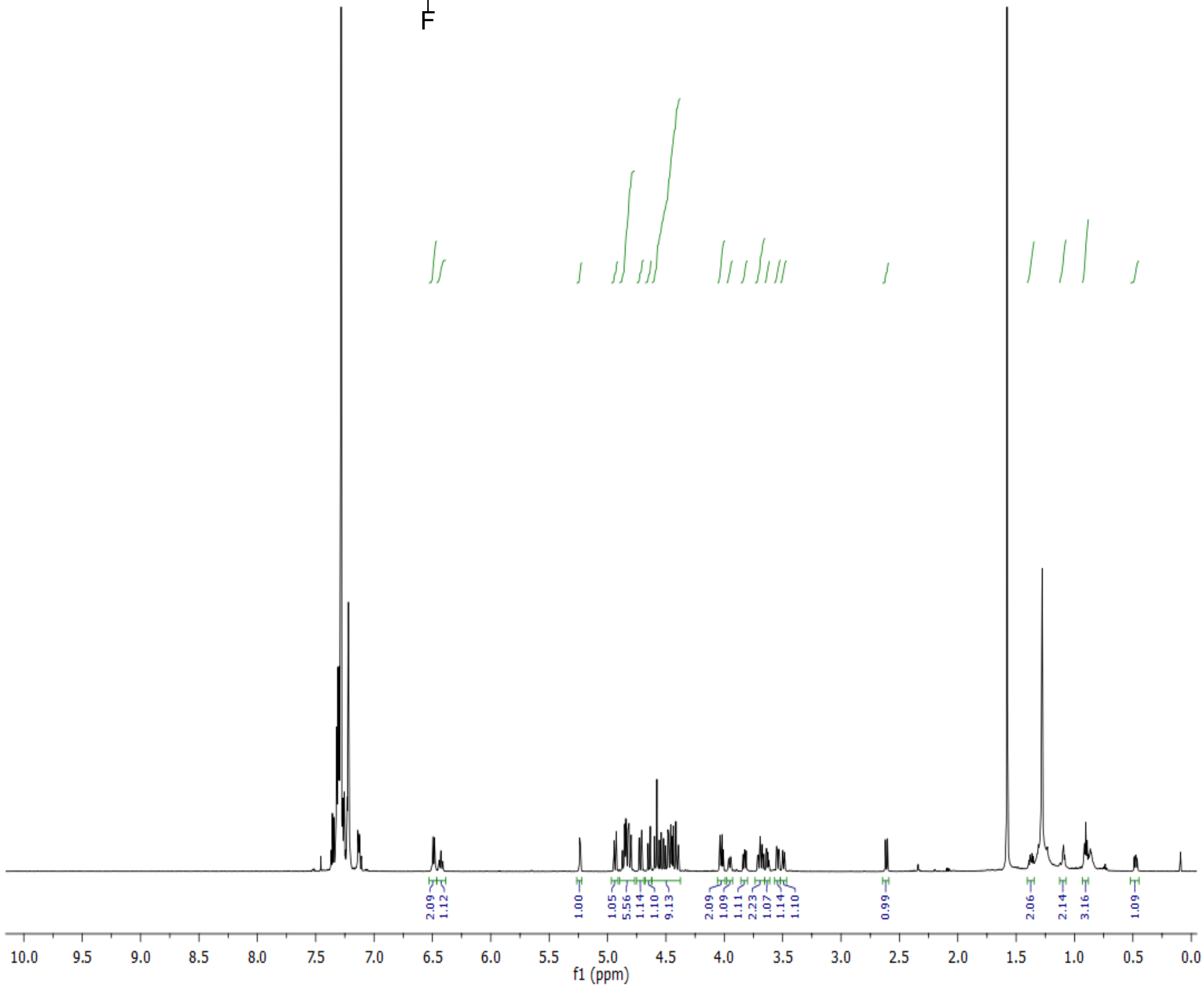

^{13}C NMR 3,4-Di(benzyloxy)-6-((benzyloxy)methyl)-5-O-(2',3',4',6'-tetra-O-benzyl-α-D-glucopyranosyl)bicyclo[4.1.0]heptane-2-(3,5-difluorophenoxy) (**24**)

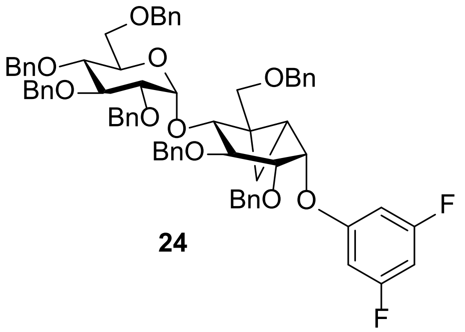

24

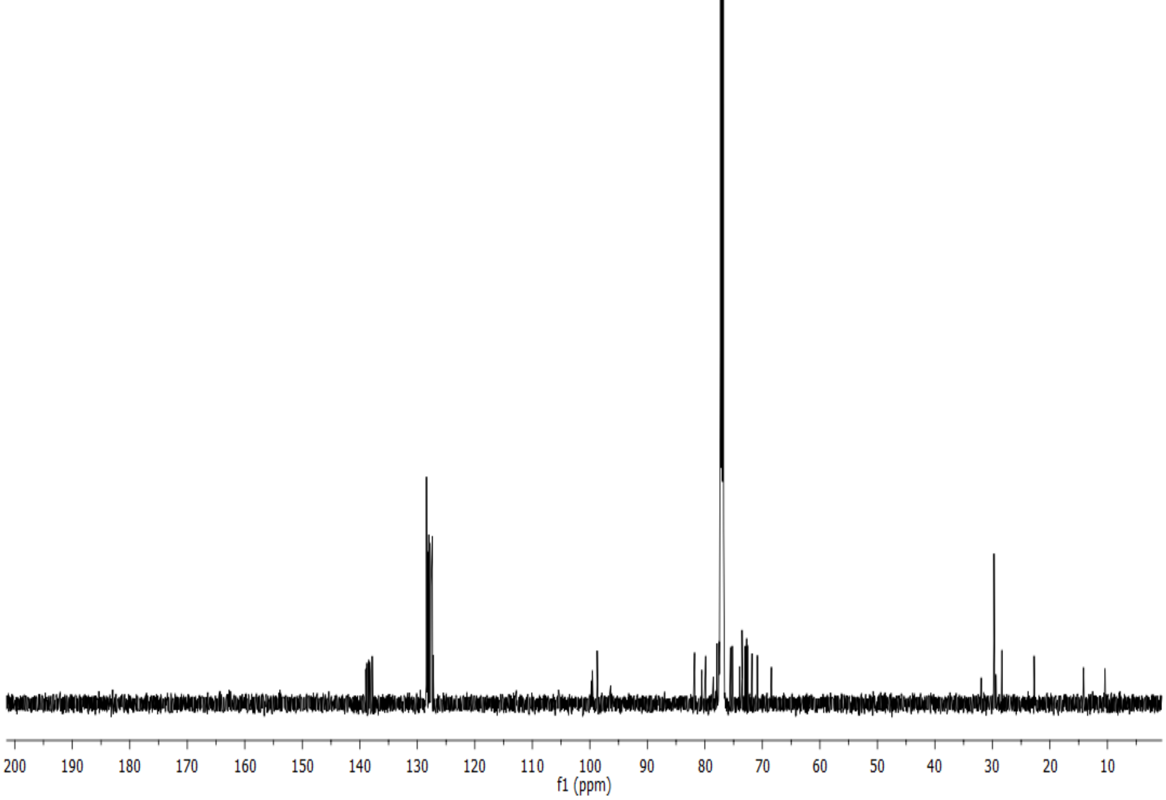

^{1}H NMR 2-(3,5-difluorophenoxy)-6-(hydroxymethyl)-5-O-(2',3',4',6'-tetra-ol-*α-D-glucopyranosyl)* bicyclo[4.1.0]heptane-3,4-diol (**11**)

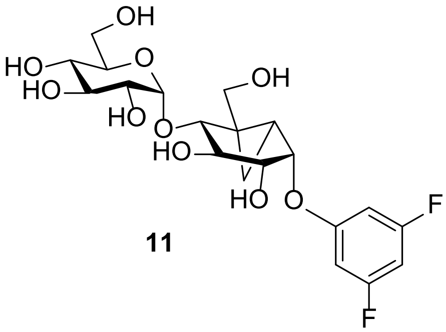

11

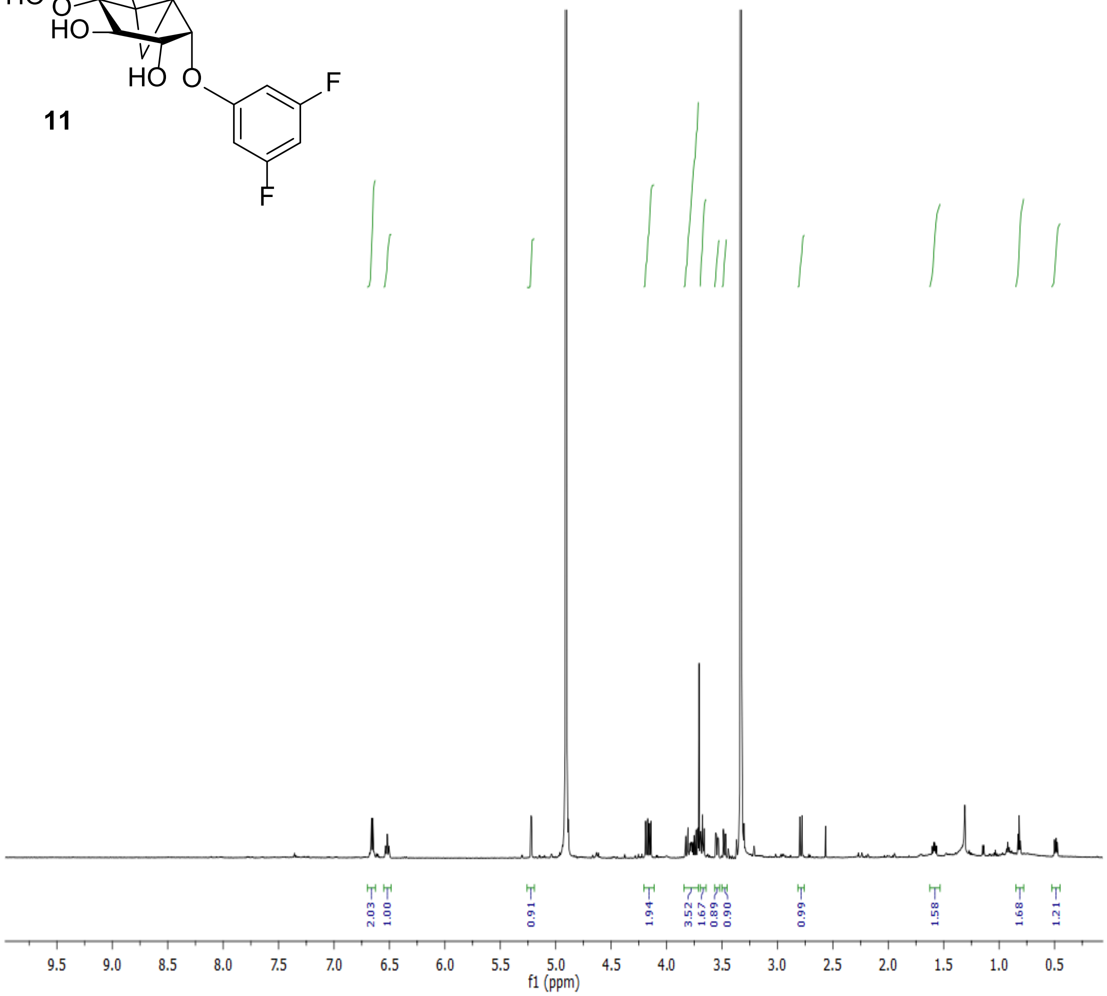

^{13}C NMR 2-(3,5-difluorophenoxy)-6-(hydroxymethyl)-5-O-(2’,3’,4’,6’-tetra-ol-*α-D-glucopyranosyl)* bicyclo[4.1.0]heptane-3,4-diol (**11**)

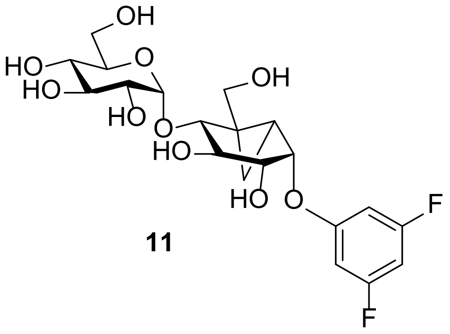

11

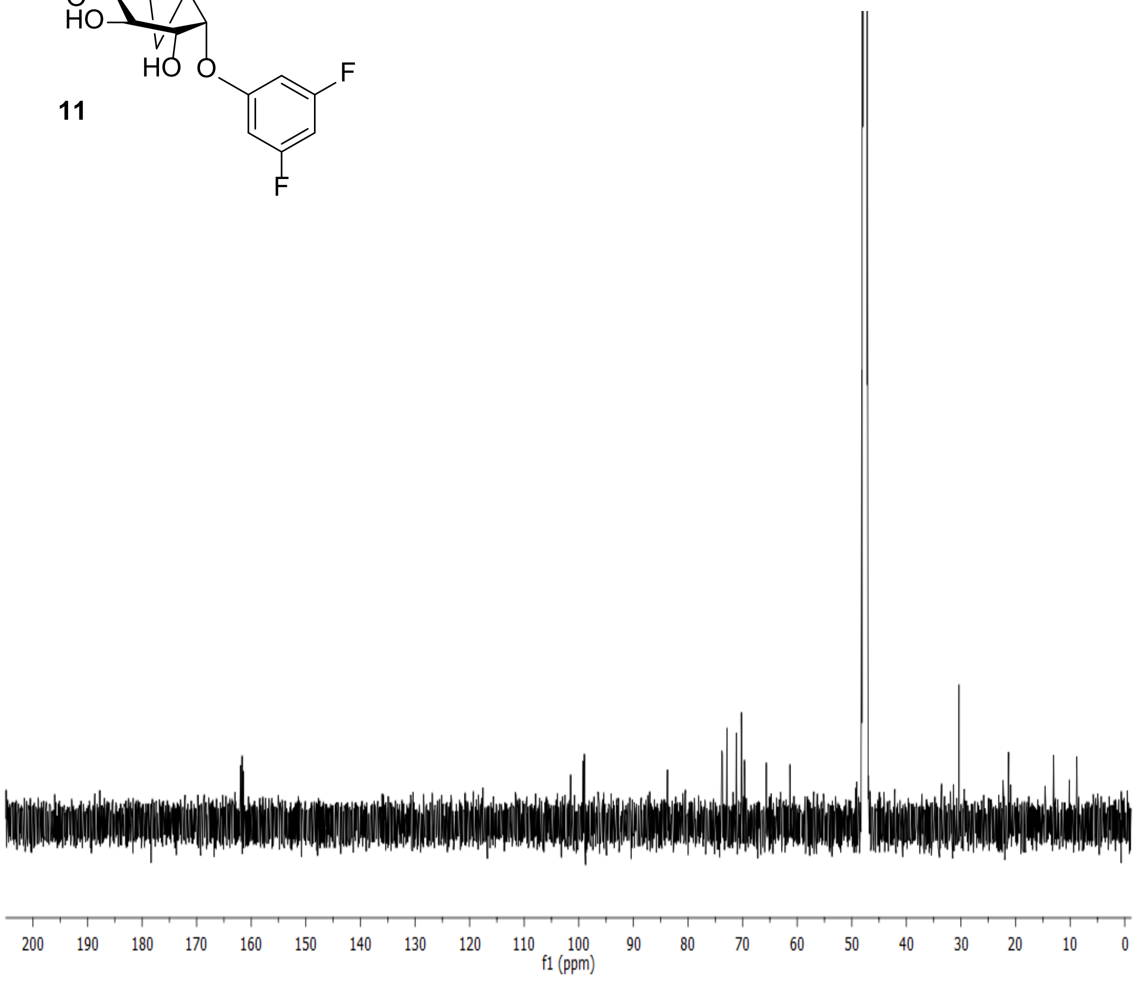

^{1}H NMR 4-methylthiophenyl 2,3,6-Tri-O-(4-bromobenzyl)-4-O-(2',3',4',6'-tetra-O-(4-bromobenzyl)-α-D-glucopyranosyl)-β-D-glucopyranoside (**15'**)

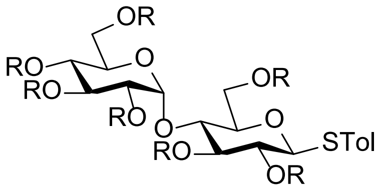

15' : R = PBB

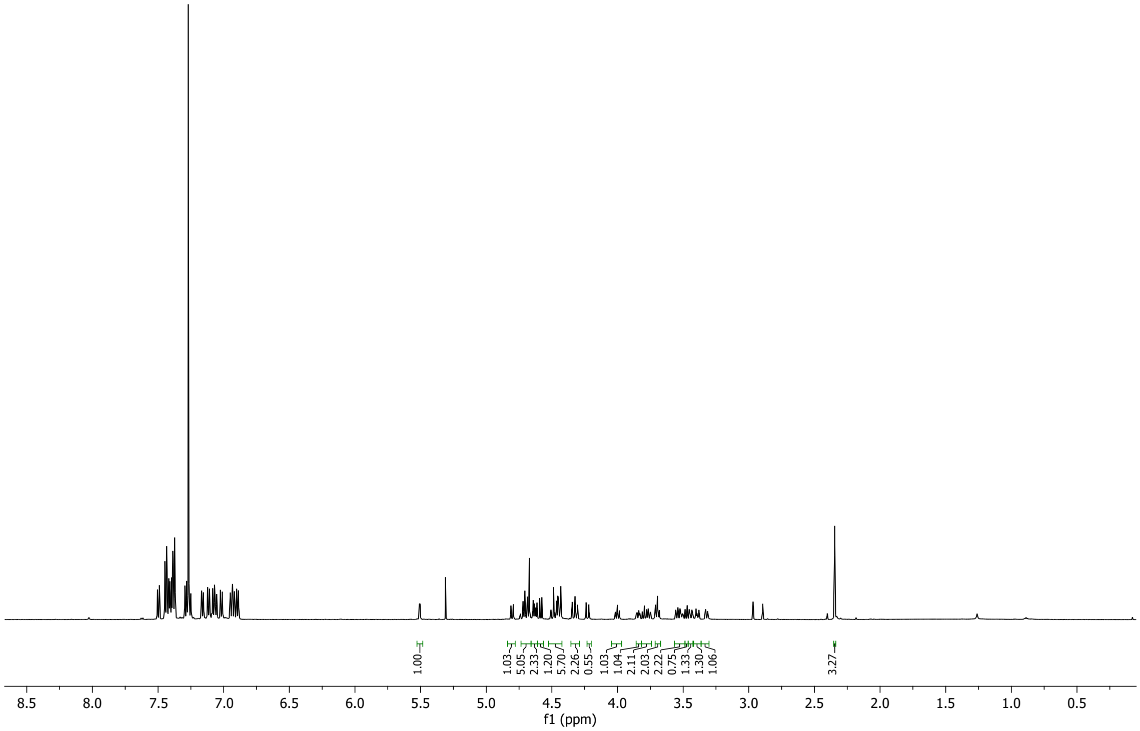

^{13}C NMR 4-methylthiophenyl 2,3,6-Tri-O-(4-bromobenzyl)-4-O-(2',3',4',6'-tetra-O-(4-bromobenzyl)-α-D-glucopyranosyl)-β-D-glucopyranoside (**15'**)

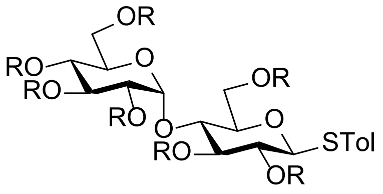

15' : R = PBB

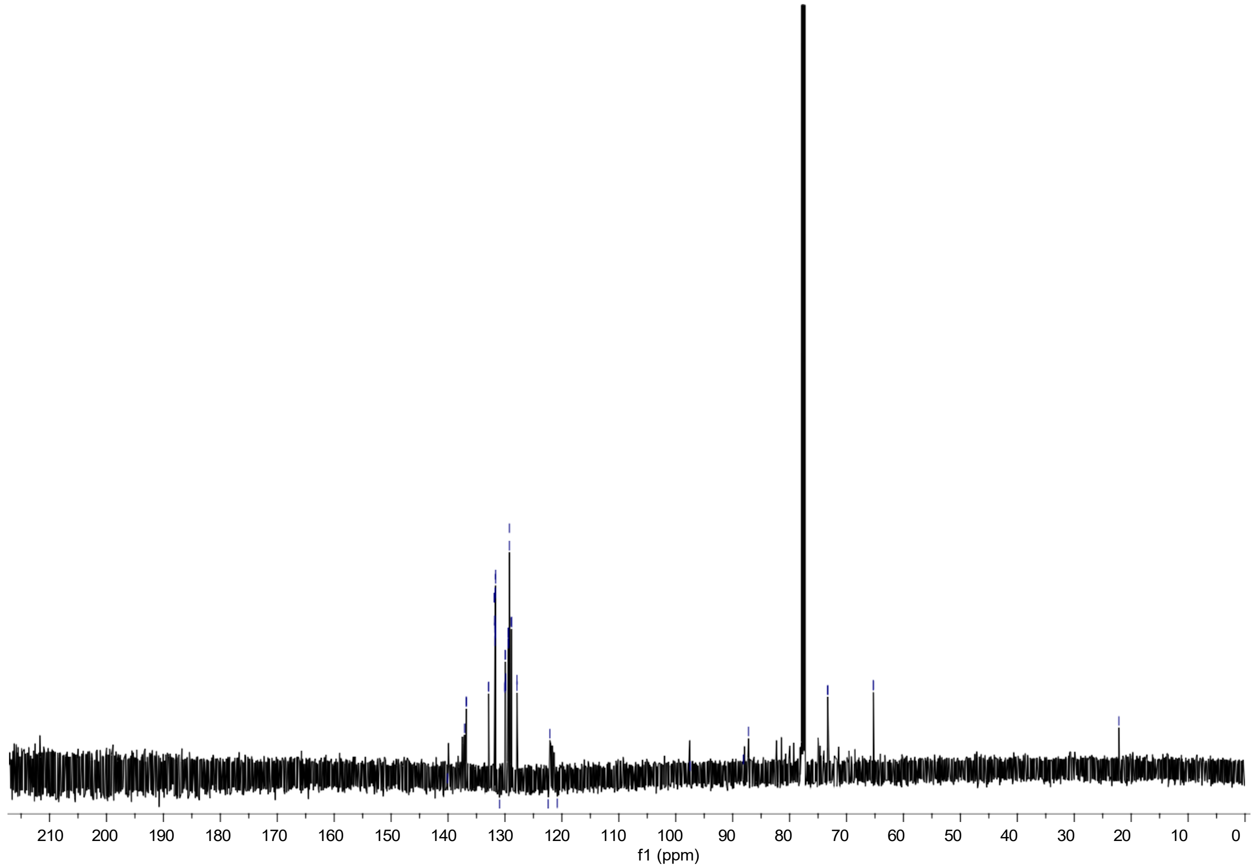

^1H NMR 2,3,6-Tri-O-(4-bromobenzyl)-4-O-(2',3',4',6'-tetra-O-(4-bromobenzyl)-α-D-glucopyranosyl)-α/β-D-glucopyranoside (**16'**)

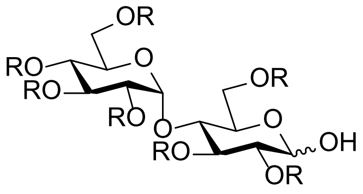

16' : R = PBB

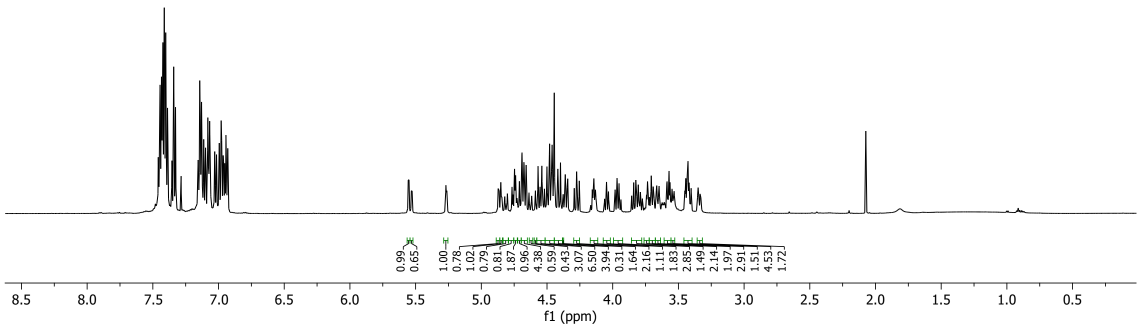

^{13}C NMR 2,3,6-Tri-O-(4-bromobenzyl)-4-O-(2',3',4',6'-tetra-O-(4-bromobenzyl)-α-D-glucopyranosyl)-α/β-D-glucopyranoside (**16'**)

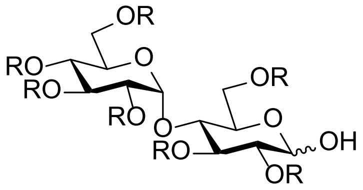

16' : R = PBB

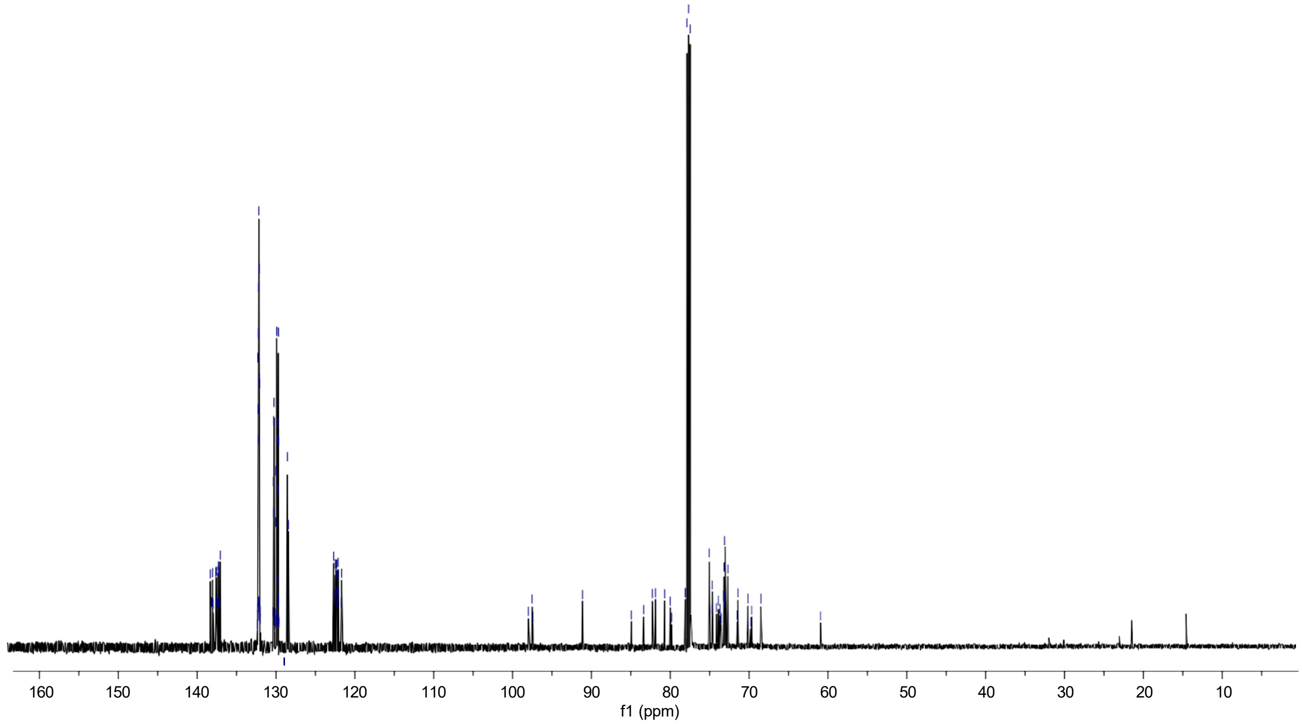

^{1}H NMR 3,4,7-Tri-*O*-(4-bromobenyl)-5-*O*-(2’,3’,4’,6’-tetra-*O*-(4-bromobenzyl)-α-D-glucopyranosyl)-D-gluchept-1-enitol (**17’**)

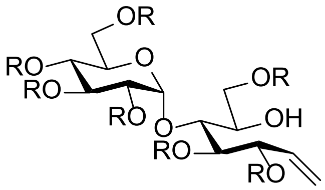

17' : R = PBB

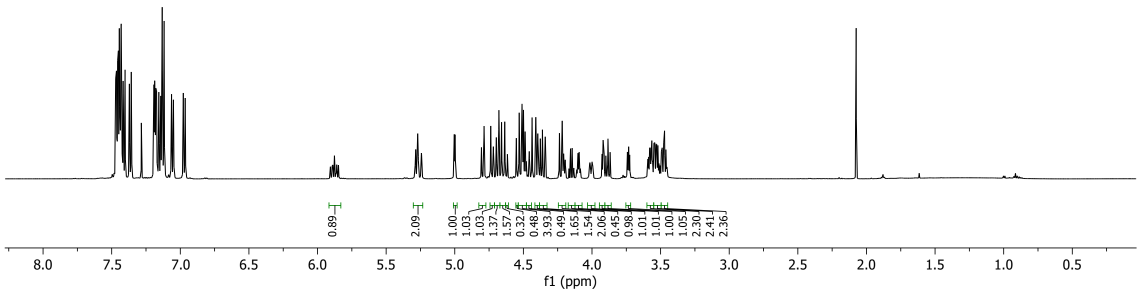

^{13}C NMR 3,4,7-Tri-*O*-(4-bromobenyl)-5-*O*-(2',3',4',6'-tetra-*O*-(4-bromobenzyl)-α-D-glucopyranosyl)-D-gluchept-1-enitol (**17'**)

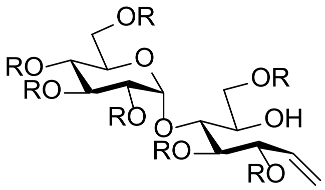

17' : R = PBB

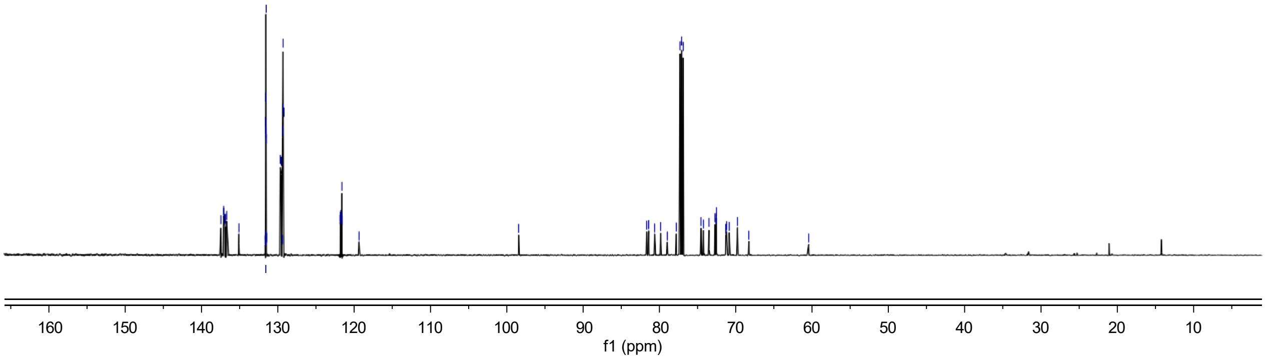

^{1}H NMR 3,4,7-Tri-O-(4-bromobenzyl)-5-O-(2',3',4',6'-tetra-O-(4-bromobenzyl)-α-D-glucopyranosyl)-D-gluchept-1-enone (**18'**)

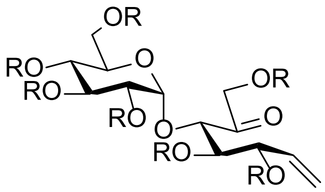

18' : R =PBB

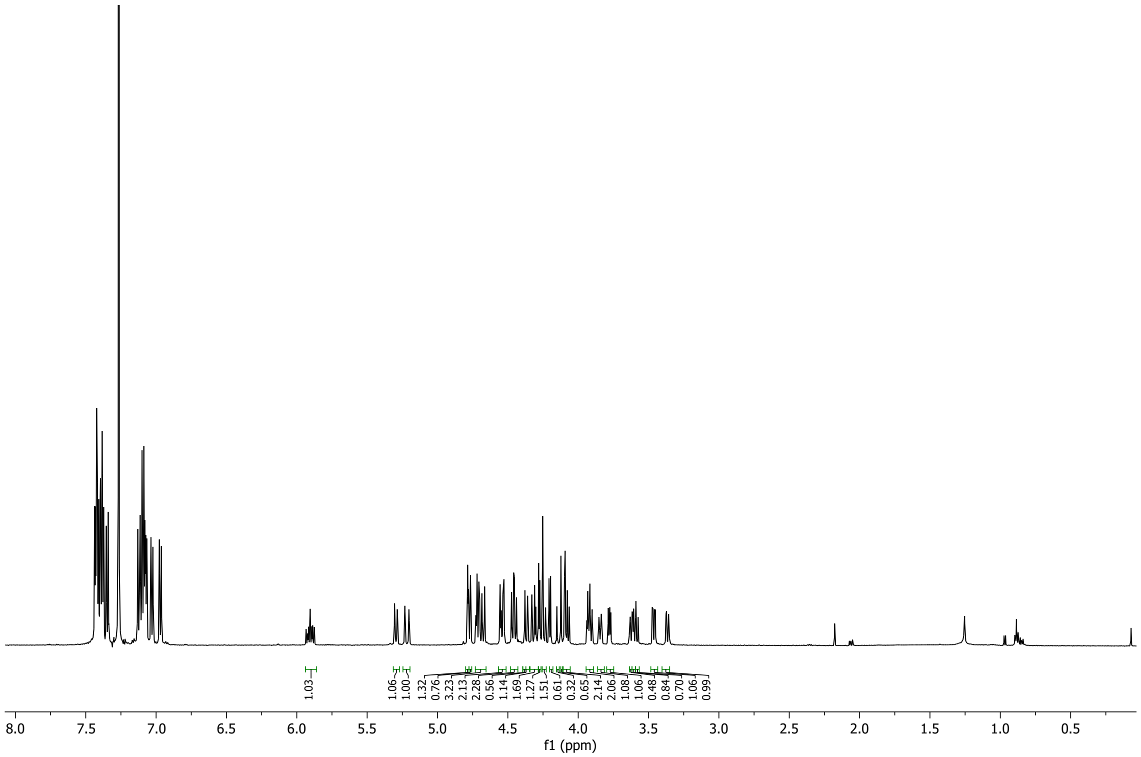

^{13}C NMR 3,4,7-Tri-O-(4-bromobenzyl)-5-O-(2',3',4',6'-tetra-O-(4-bromobenzyl)-α-D-glucopyranosyl)-D-gluchept-1-enone (**18'**)

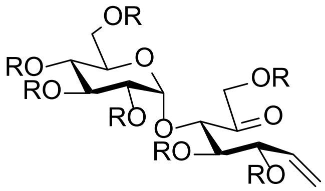

18' : R =PBB

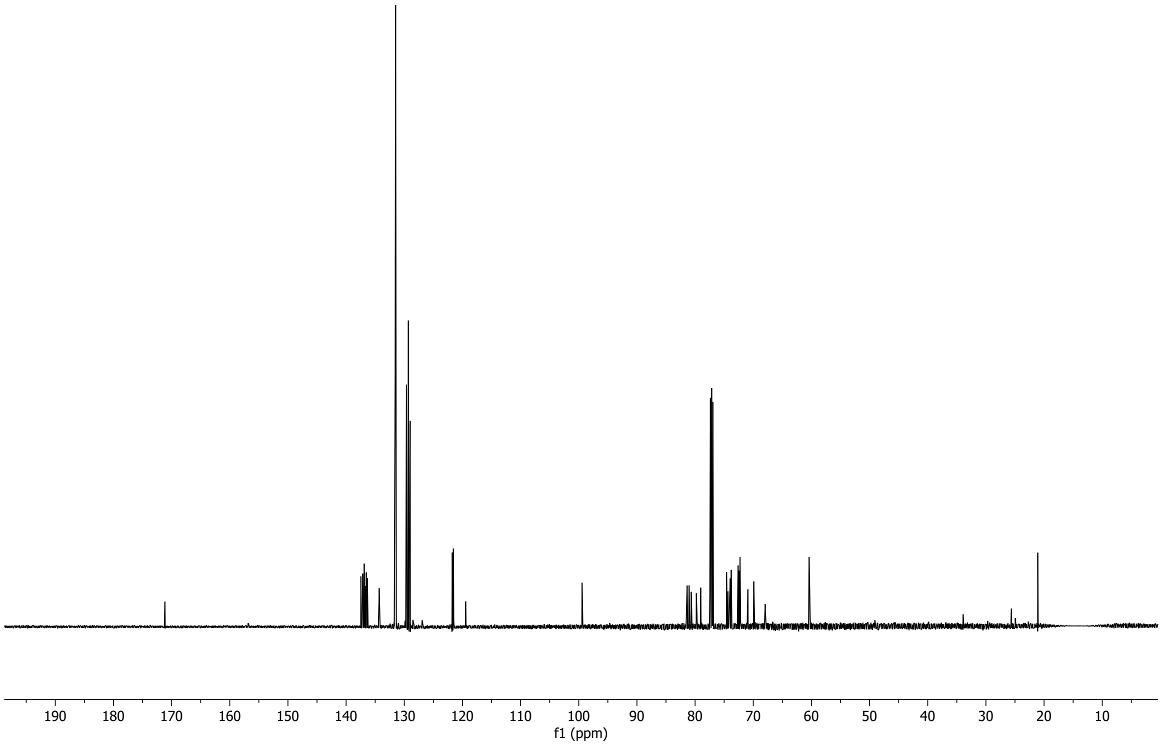

^{1}H NMR 3,4,9-Tri-O-(4-bromobenzyl)-5-O-(2’,3’,4’,6’-tetra-O-(4-bromobenzyl)-α-D-gucopyranosyl)-D-gluco-octa-1,7-dienitol (**19A’**)

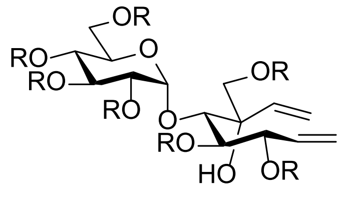

19A': R = PBB

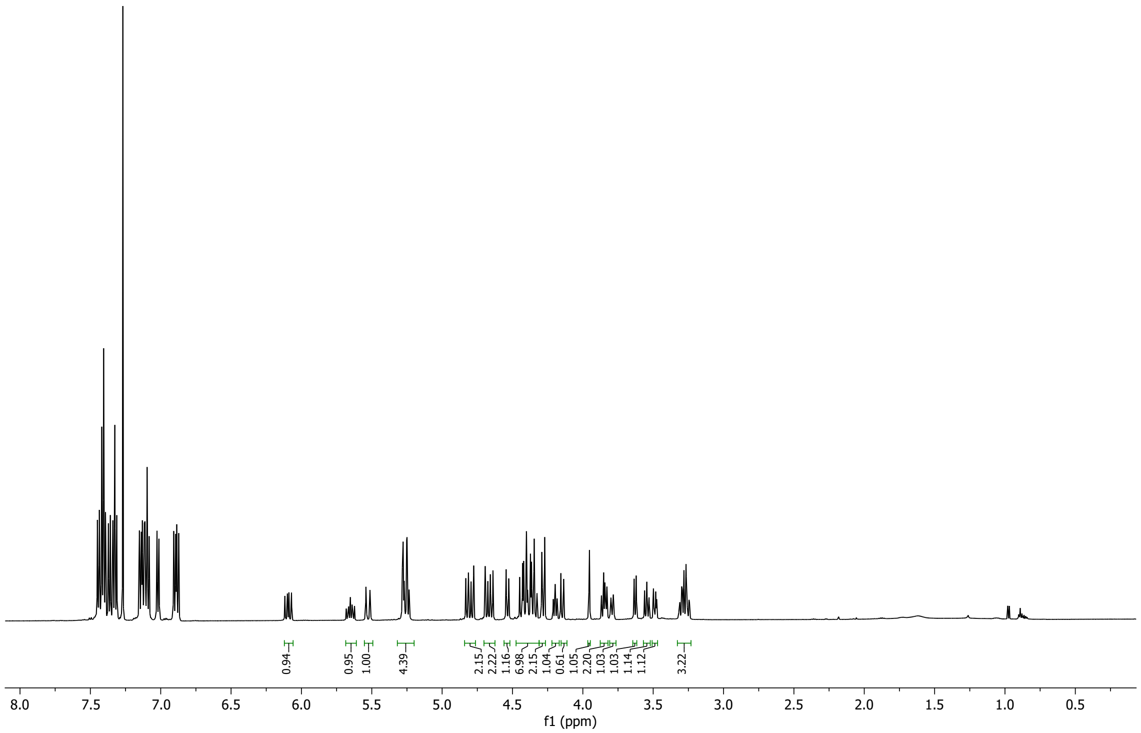

^{13}C NMR 3,4,9-Tri-O-(4-bromobenzyl)-5-O-(2',3',4',6'-tetra-O-(4-bromobenzyl)-α-D-glucopyranosyl)-D-gluco-octa-1,7-dienitol (**19A'**)

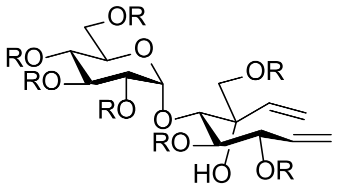

19A': R = PBB

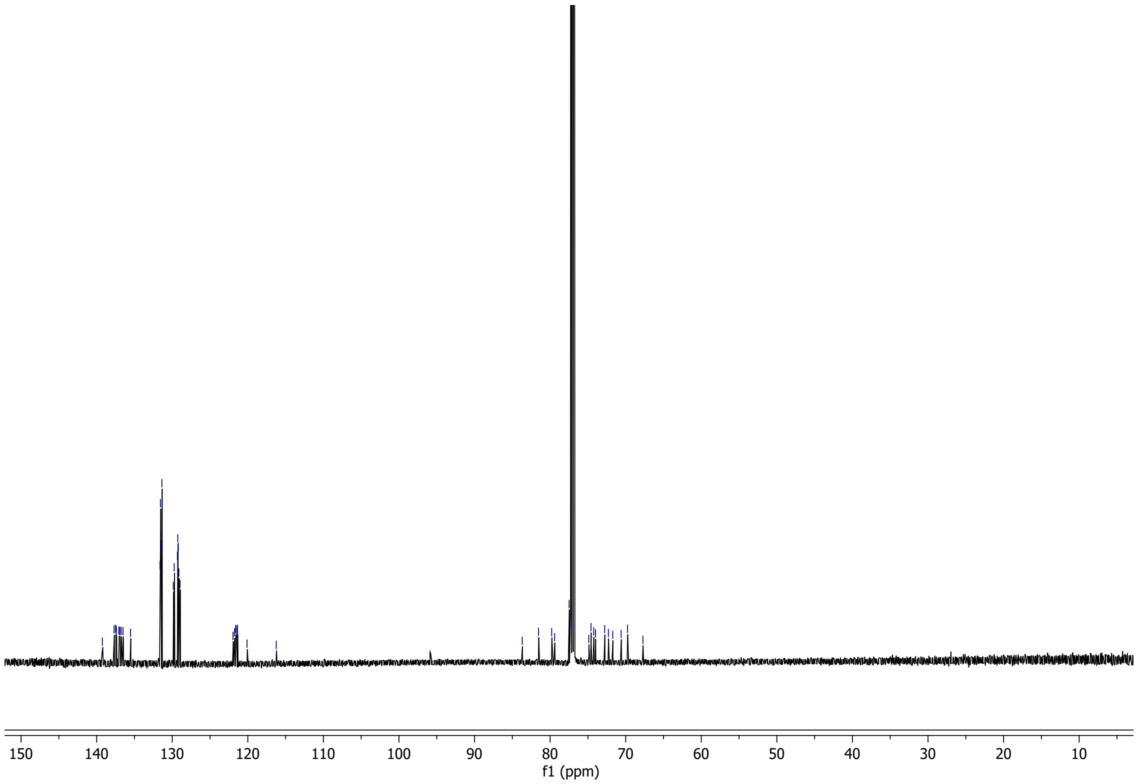

^{1}H NMR 3,4,9-Tri-*O*-(4-bromobenzyl)-5-*O*-(2',3',4',6'-tetra-*O*-(4-bromobenzyl)-α-D-glucopyranosyl)-L-ido-octa-1,7-dienitol (**19B'**)

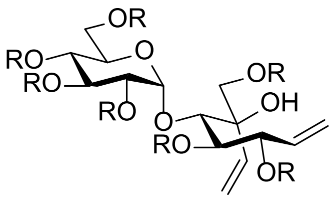

19B' : R = PBB

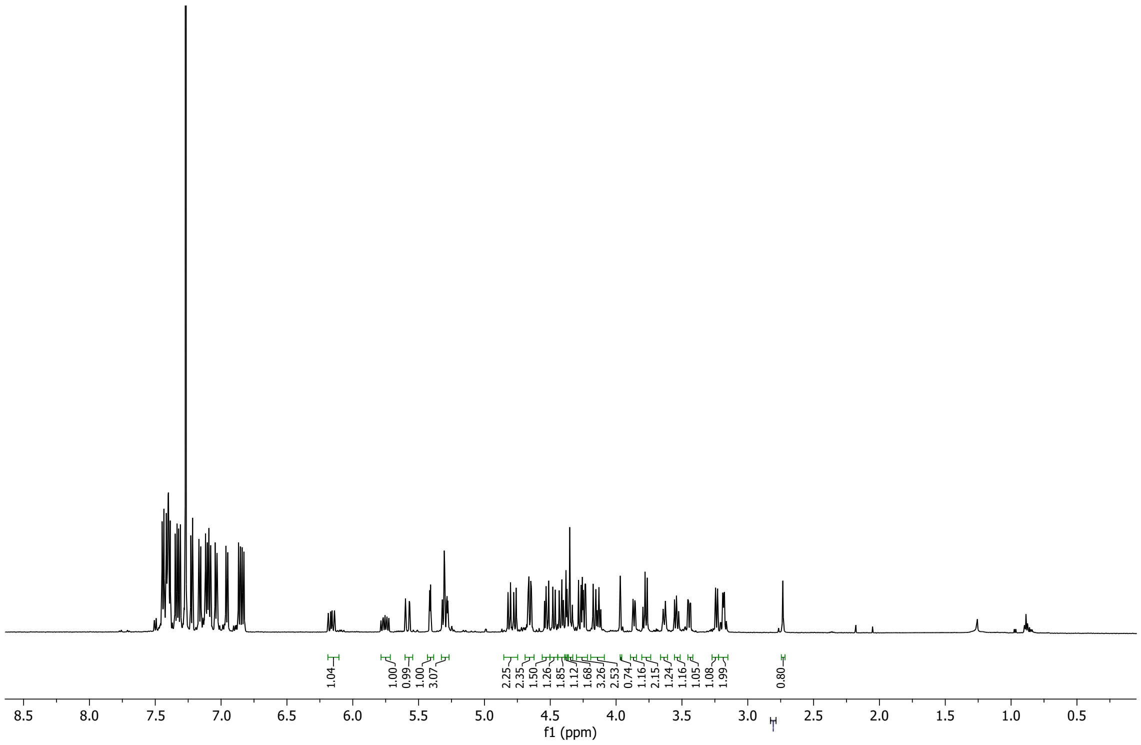

^1H NMR (1,3,4/2)-1,2-Di-O-(4-bromobenzyl)-4-C-[(4-bromobenzyloxy)methyl]-3-O-(2',3',4',6'-tetra-O-(4-bromobenzyl)-α-D-glucopyranosyl)cyclohex-5-ene-1,2,3,4-tetrol (**10'**)

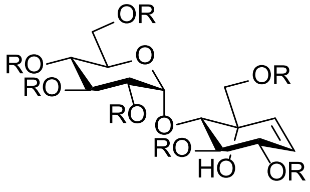

10' : R = PBB

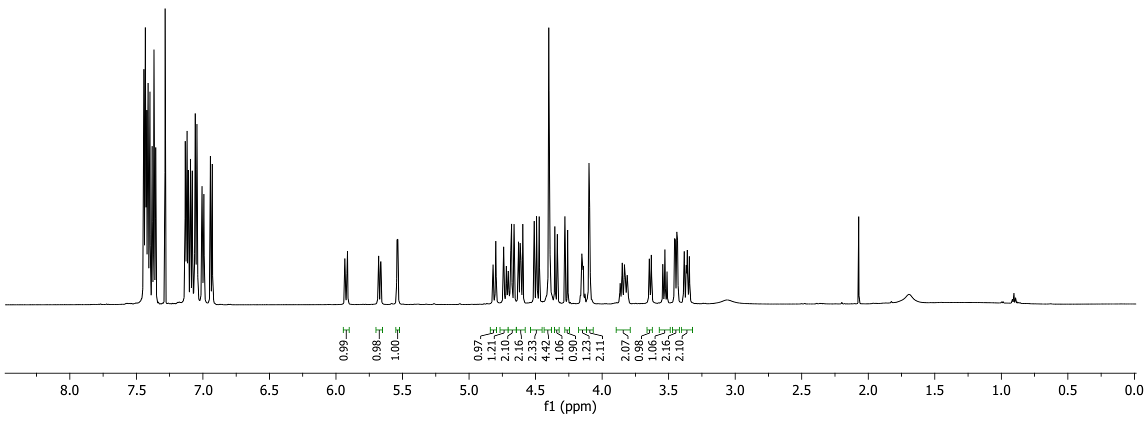

^{13}C NMR (1,3,4/2)-1,2-Di-O-(4-bromobenzyl)-4-C-[(4-bromobenzyloxy)methyl]-3-O-(2',3',4',6'-tetra-O-(4-bromobenzyl)-α-D-glucopyranosyl)cyclohex-5-ene-1,2,3,4-tetrol (**10'**)

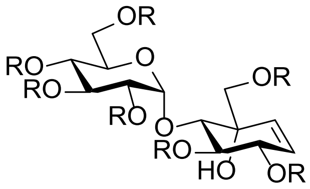

10' : R = PBB

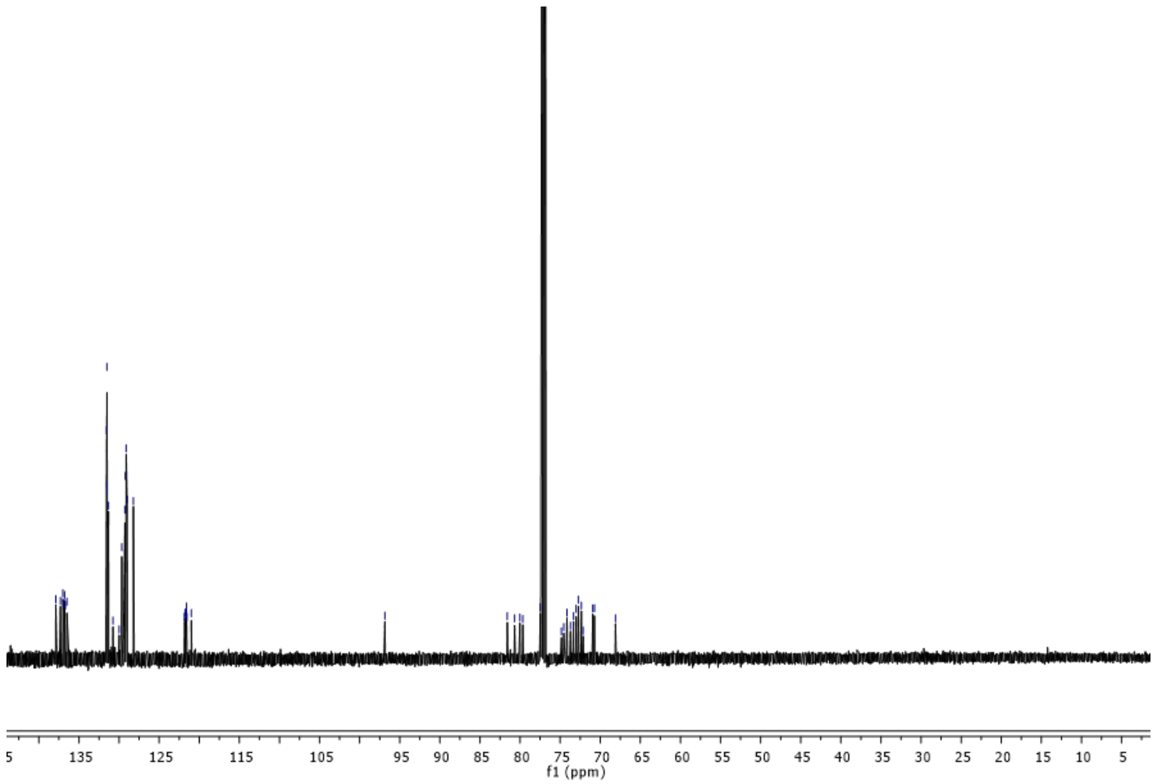

^{1}H NMR (1,3,4/2)-1,2-Di-O-(4-bromobenzyl)-4-C-[(4-bromobenzyloxy)methyl]-4-O-acetyl-3-O-(2',3',4',6'-tetra-O-(4-bromobenzyl)-α-D-glucopyranosyl)cyclohex-5-ene-1,2,3,4-tetrol (**20'**)

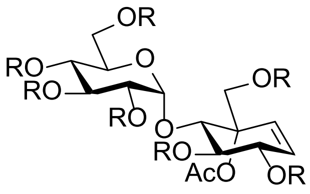

20' : R = PBB

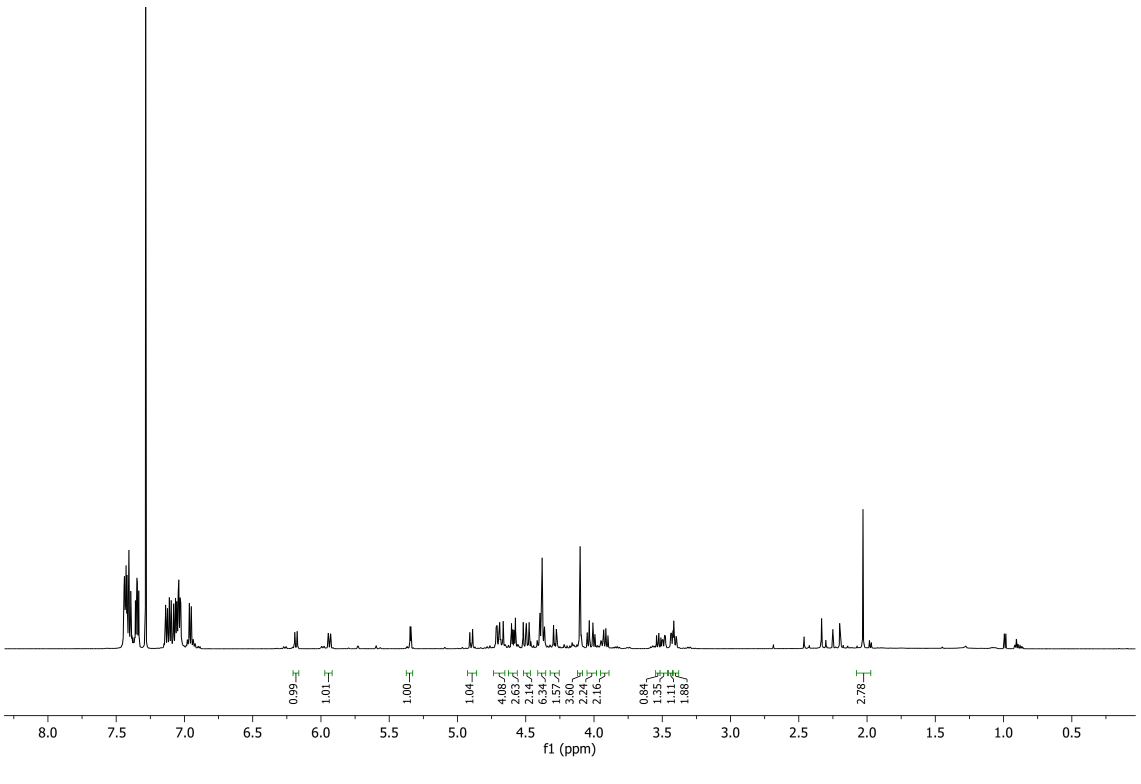

^{13}C NMR (1,3,4/2)-1,2-Di-O-(4-bromobenzyl)-4-C-[(4-bromobenzyloxy)methyl]-4-O-acetyl-3-O-(2',3',4',6'-tetra-O-(4-bromobenzyl)-α-D-glucopyranosyl)cyclohex-5-ene-1,2,3,4-tetrol (**20'**)

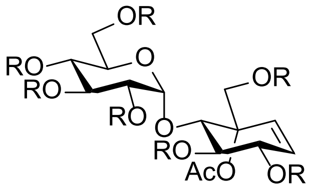

20' : R = PBB

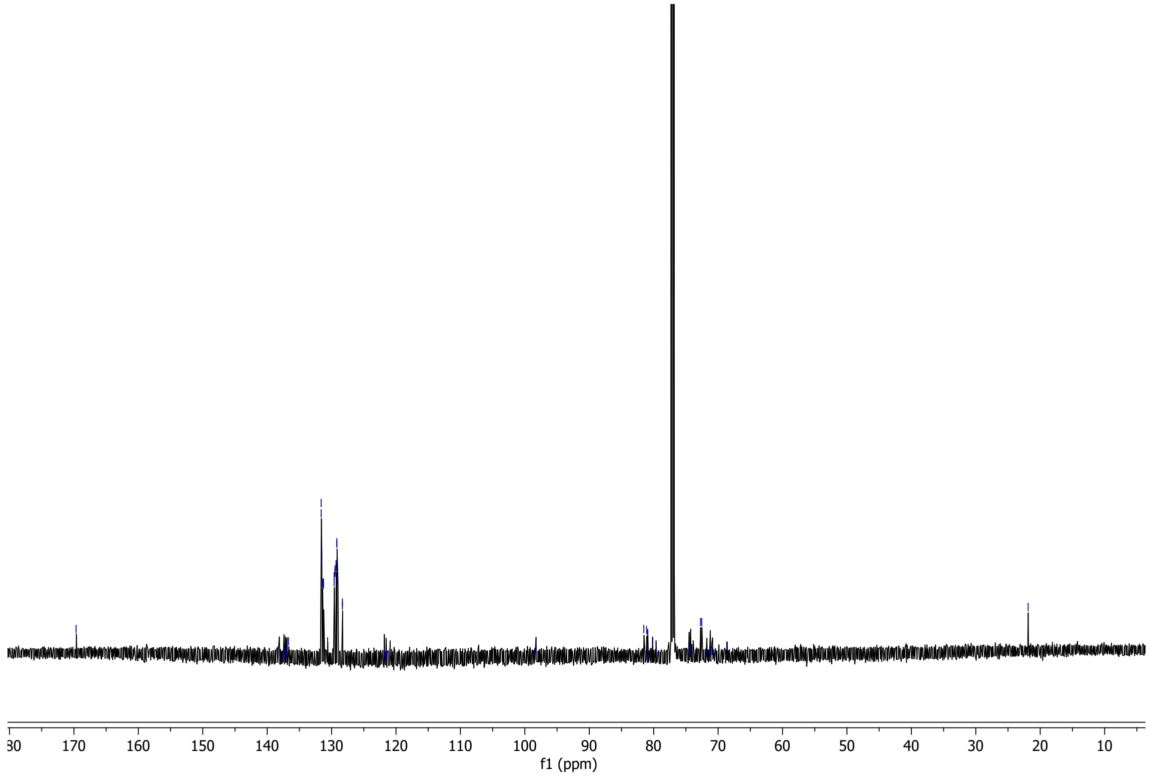

^{1}H NMR (1,3,4/2)-1,2-Di-O-(4-bromobenzyl)-4-C-[(4-bromobenzyloxy)methyl]-3-O-(2’,3’,4’,6’-tetra-O-(4-bromobenzyl)-α-D-glucopyranosyl)cyclohex-4-ene-1,2,3,6-tetrol (**21’**)

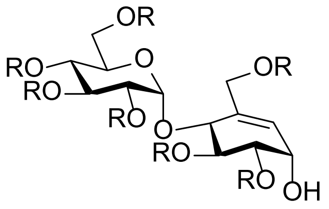

21' : R = PBB

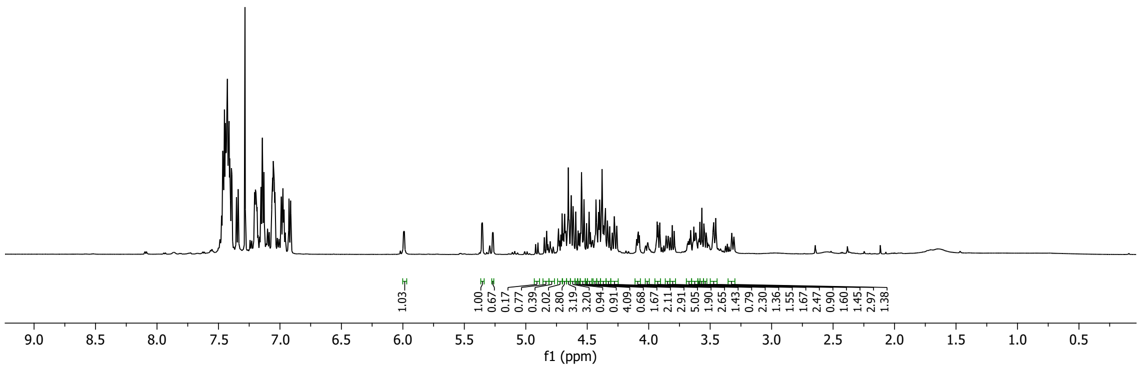

^{13}C NMR (1,3,4/2)-1,2-Di-O-(4-bromobenzyl)-4-C-[(4-bromobenzyloxy)methyl]-3-O-(2',3',4',6'-tetra-O-(4-bromobenzyl)-α-D-glucopyranosyl)cyclohex-4-ene-1,2,3,6-tetrol (**21'**)

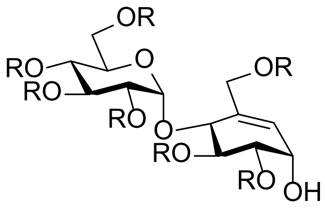

21' : R = PBB

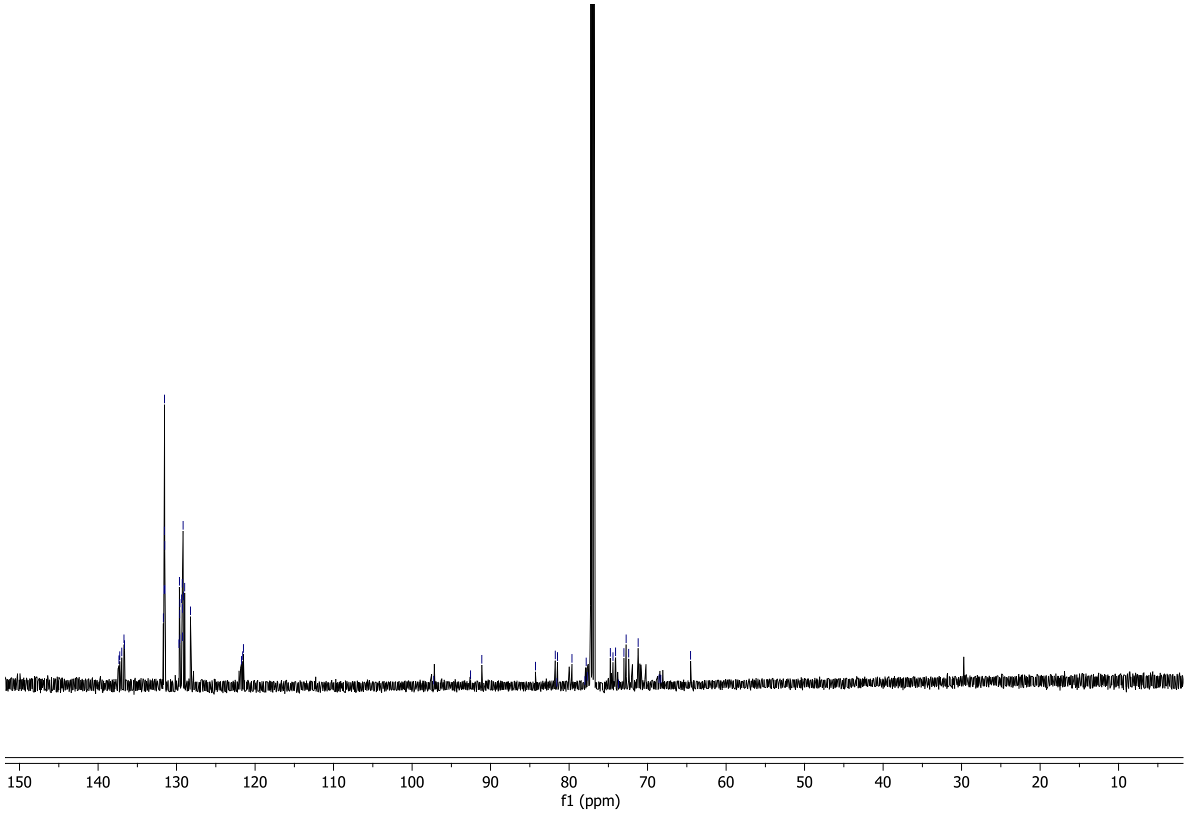

^{1}H NMR 3,4-Di(4-bromobenzyloxy)-6-((4-bromobenzyloxy)methyl)-5-O-(2’,3’,4’,6’-tetra-O-4-bromobenzyl-α-D-glucopyranosyl)- 2-(3,5-difluorophenoxy) (**23**)

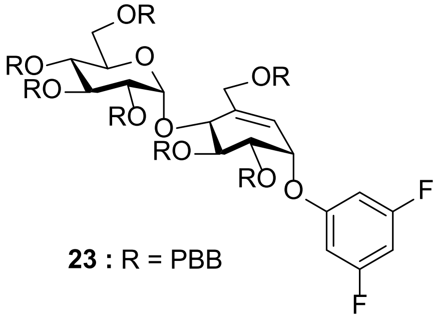

23 : R = PBB

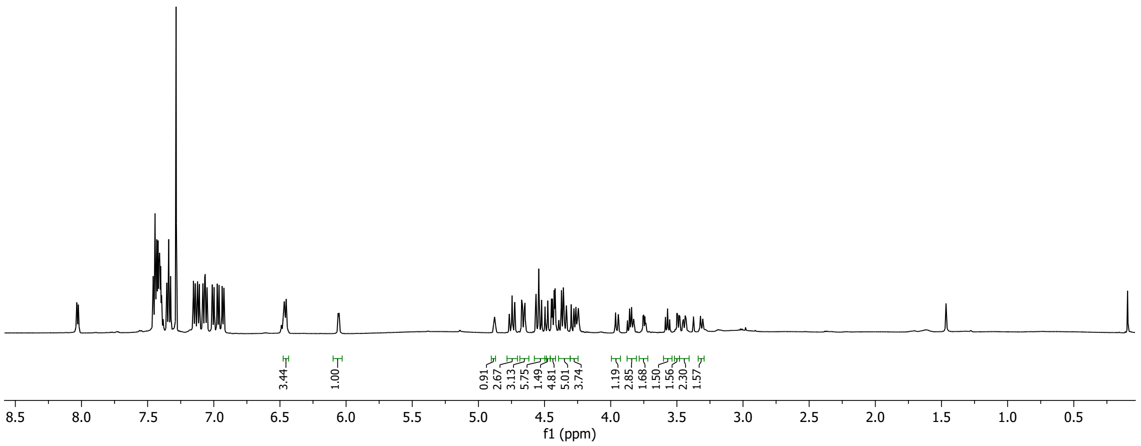

^{13}C NMR 3,4-Di(4-bromobenzyloxy)-6-((4-bromobenzyloxy)methyl)-5-O-(2',3',4',6'-tetra-O-4-bromobenzyl-α-D-glucopyranosyl)- 2-(3,5-difluorophenoxy) (**23**)

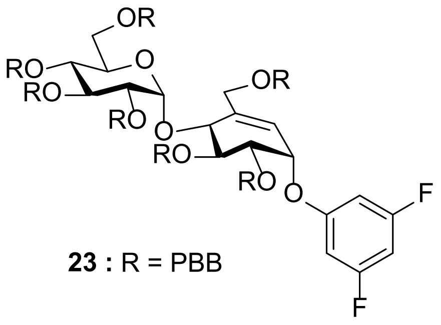

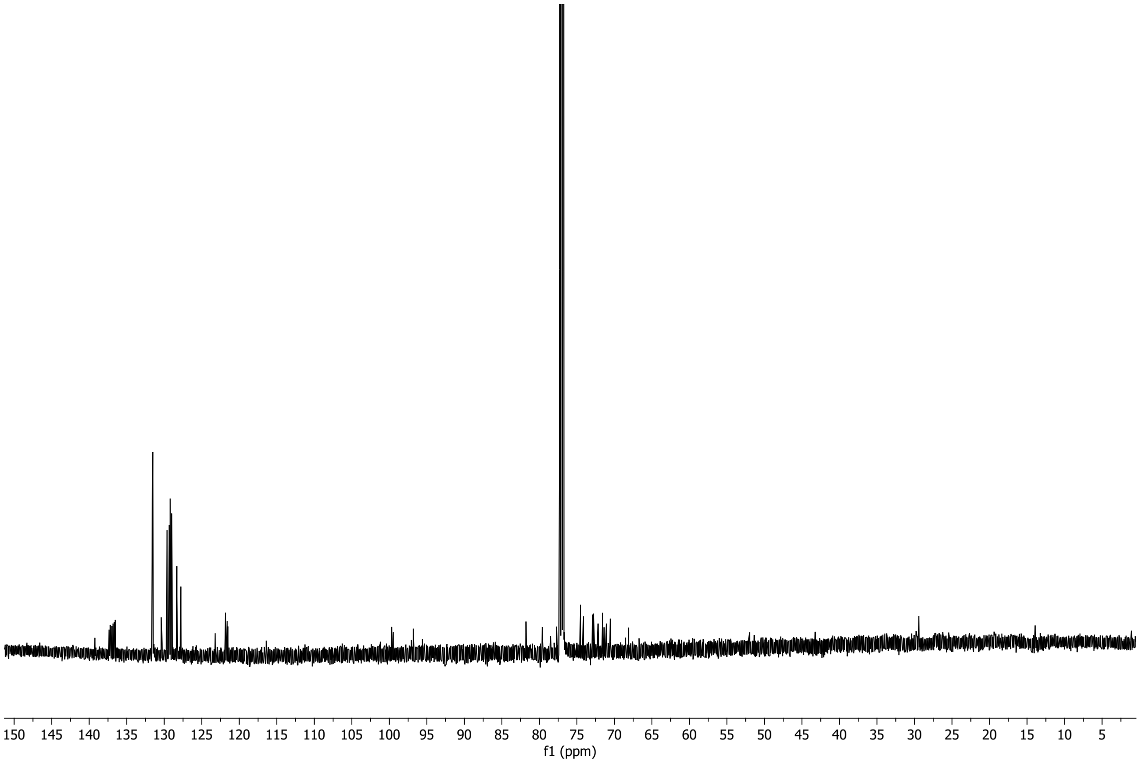

^{1}H NMR 3,4-Di(4-(N-methyl, N-phenyl)benzyloxy)-6-((4-(N-methyl, N-phenyl)benzyloxy)methyl)-5-O-(2’,3’,4’,6’-tetra-O-4-(N-methyl,N-phenyl)benzyl-α-D-glucopyranosyl)- 2-(3,5-difluorophenoxy) (**25**)

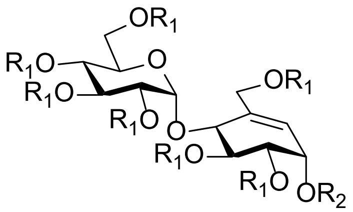

25 : R_1 = (4-*N*-*m*ethyl, *N*-*p*henylamino) benzyl, R_2 = 3,5-difluorophenyl

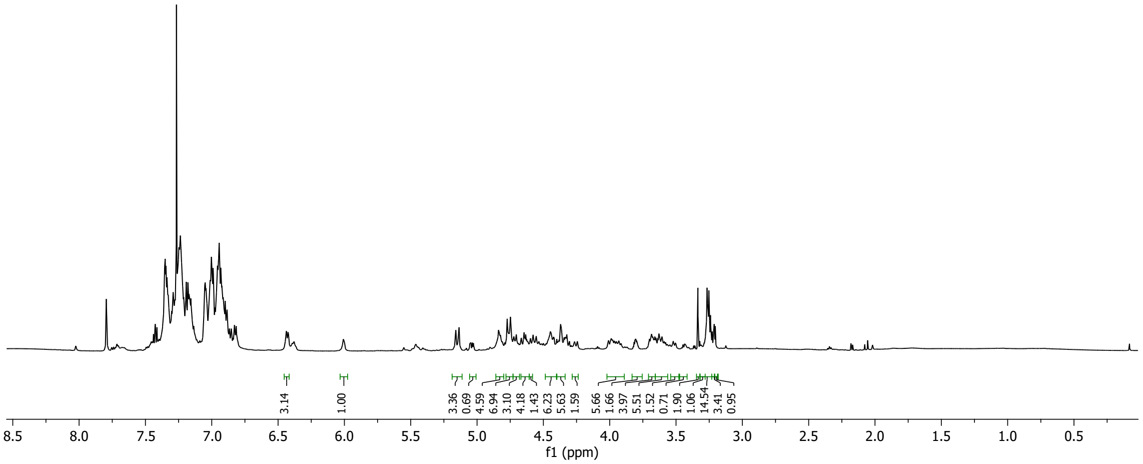

^{13}C NMR 3,4-Di(4-(N-methyl, N-phenyl)benzyloxy)-6-((4-(N-methyl, N-phenyl)benzyloxy)methyl)-5-O-(2’,3’,4’,6’-tetra-O-4-(N-methyl, N-phenyl)benzyl-α-D-glucopyranosyl)- 2-(3,5-difluorophenoxy) (**25**)

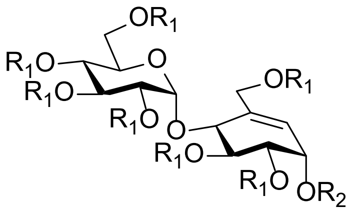

25 : R_1 = (4-*N*-*m*ethyl, *N*-*p*henylamino) benzyl, R_2 = 3,5-difluorophenyl

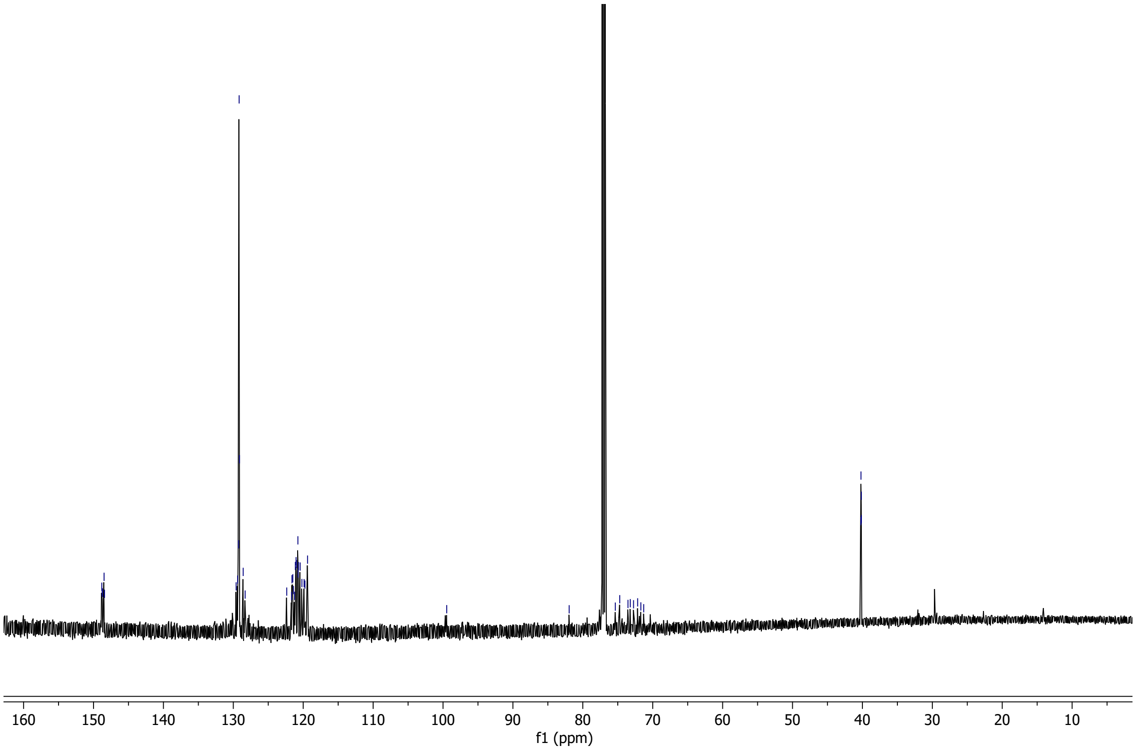

^{1}H NMR 1-α-D-Glucopyranoside 4-(hydroxymethyl)-6- (4-(3,5 difluorophenoxy))

cyclohex-4-ene-2,3-triol (**12**)

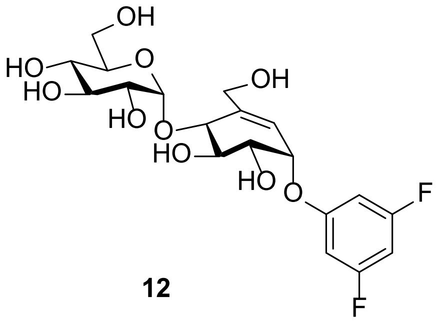

12

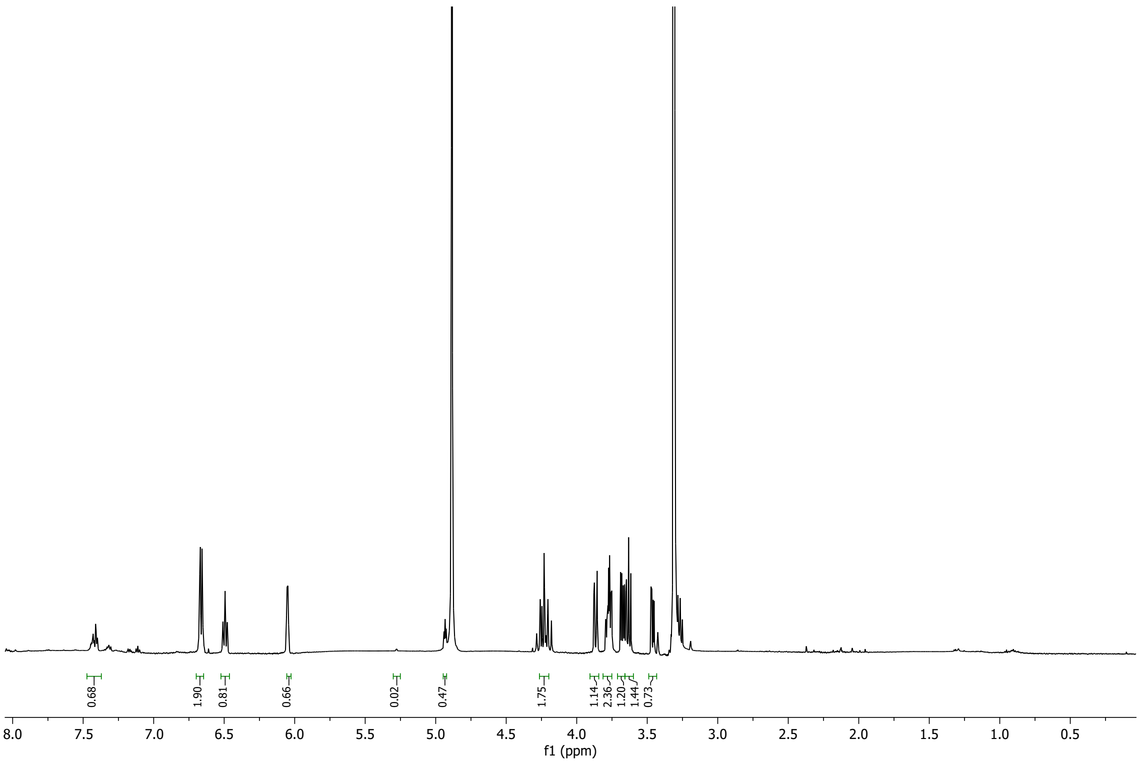

^{13}C NMR 1-α-D-Glucopyranoside 4-(hydroxymethyl)-6- (4-(3,5 difluorophenoxy))

cyclohex-4-ene-2,3-triol (**12**)

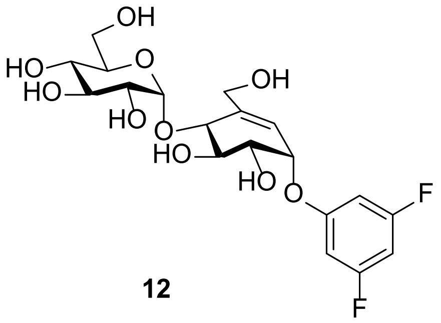

12

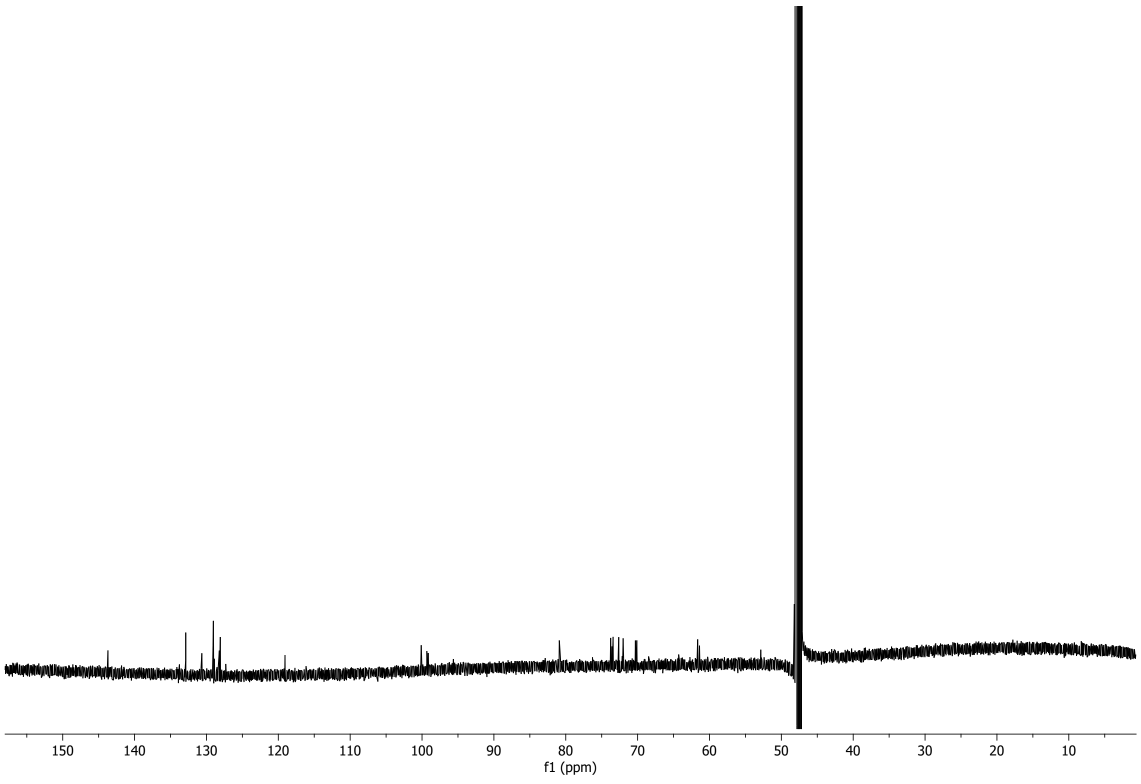

^{1}H NMR (2R,3R,4S,5R,6S)-3,4,5-tris(benzyloxy)-2-((benzyloxy)methyl)-6-(((1R,5R,6S)-5,6-bis(benzyloxy)-2-((benzyloxy)methyl)-4-bromocyclohex-2-en-1-yl)oxy)tetrahydro-2H-pyran (**26a**)

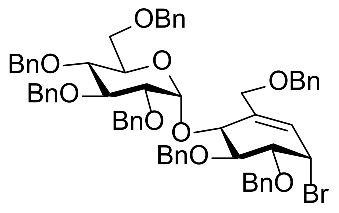

26a

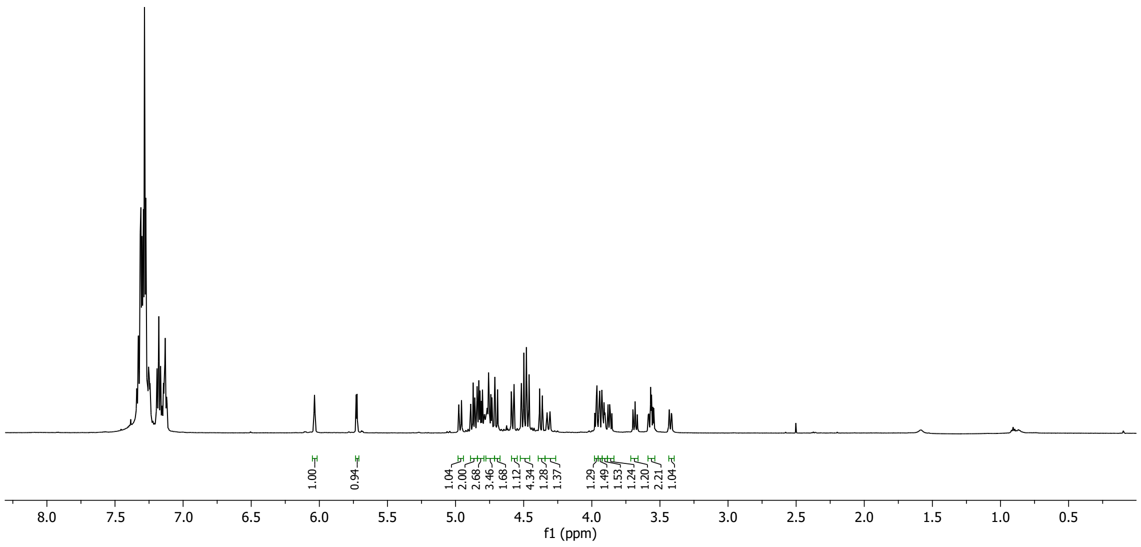

^{13}C NMR (2R,3R,4S,5R,6S)-3,4,5-tris(benzyloxy)-2-((benzyloxy)methyl)-6-(((1R,5R,6S)-5,6-bis(benzyloxy)-2-((benzyloxy)methyl)-4-bromocyclohex-2-en-1-yl)oxy)tetrahydro-2H-pyran (**26a**)

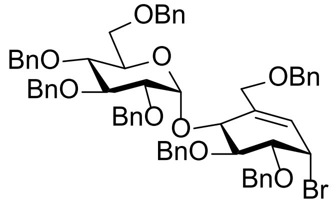

26a

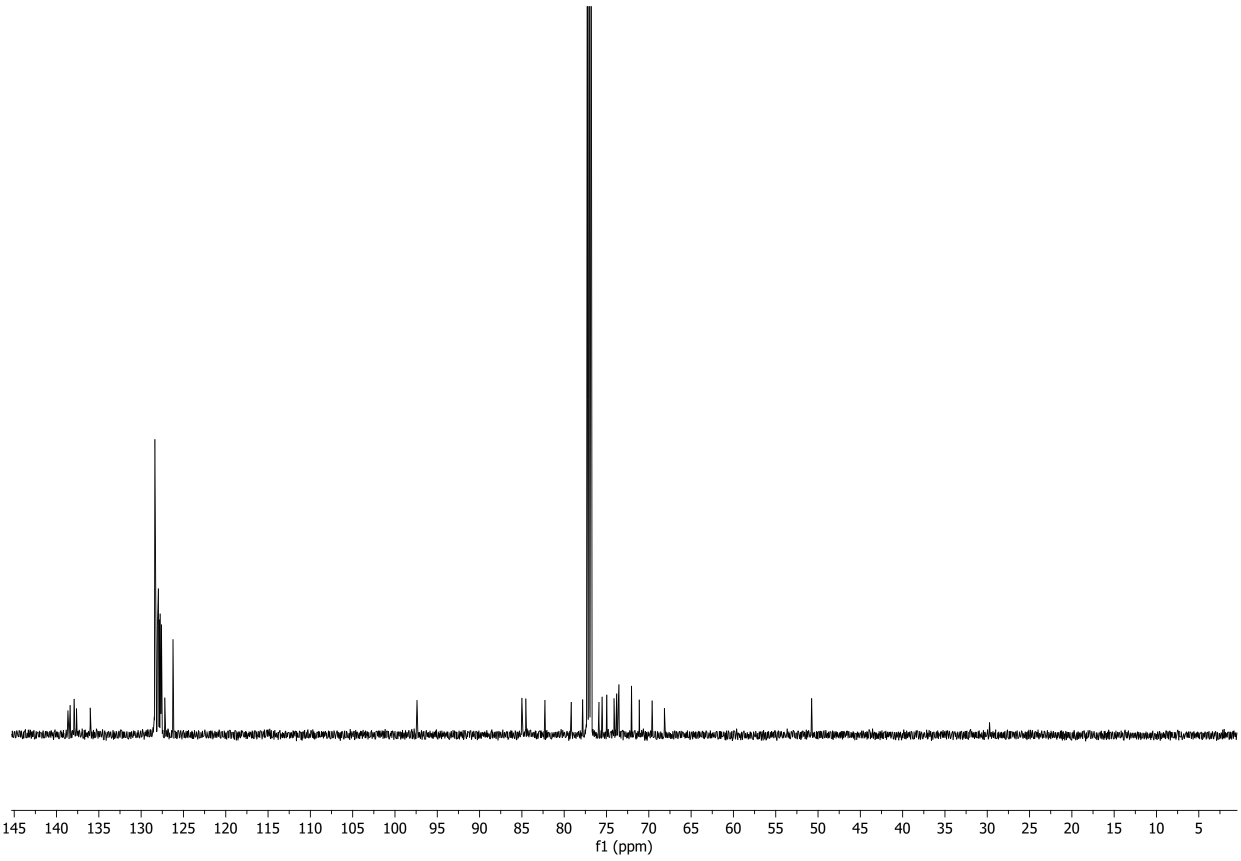

^1H NMR (2R,3R,4S,5R,6S)-3,4,5-tris(benzyloxy)-2-((benzyloxy)methyl)-6-(((1R,5R,6S)-5,6-bis(benzyloxy)-2-((benzyloxy)methyl)-4-bromocyclohex-2-en-1-yl)oxy)tetrahydro-2H-pyran (**26b**)

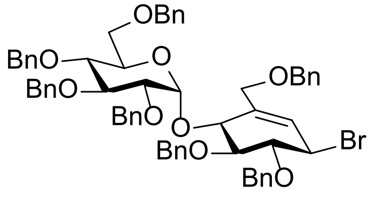

26b

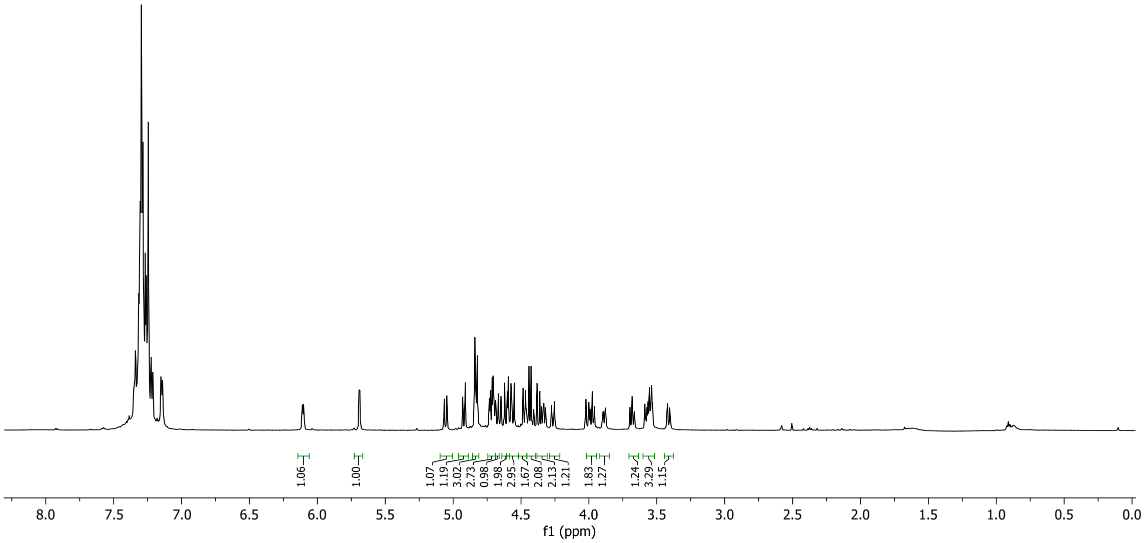

^{13}C NMR (2R,3R,4S,5R,6S)-3,4,5-tris(benzyloxy)-2-((benzyloxy)methyl)-6-(((1R,5R,6S)-5,6-bis(benzyloxy)-2-((benzyloxy)methyl)-4-bromocyclohex-2-en-1-yl)oxy)tetrahydro-2H-pyran (**26b**)

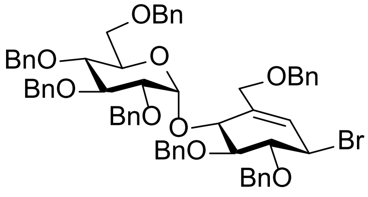

26b

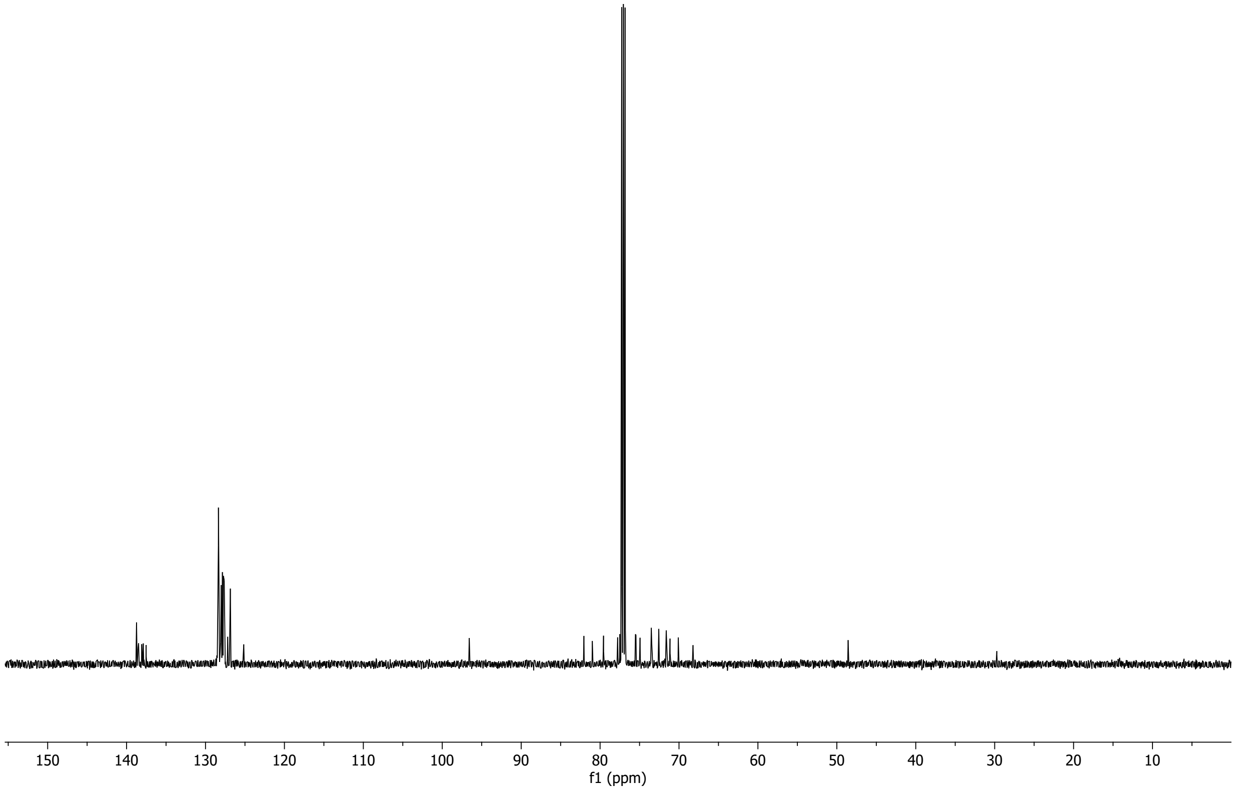

^{1}H NMR (1S,4R,5S,6S)-5,6-bis(benzyloxy)-3-((benzyloxy)methyl)-N-cyclohexyl-4-(((2S,3R,4S,5R,6R)-3,4,5-tris(benzyloxy)-6-((benzyloxy)methyl)tetrahydro-2H-pyran-2-yl)oxy)cyclohex-2-en-1-amine (**27**)

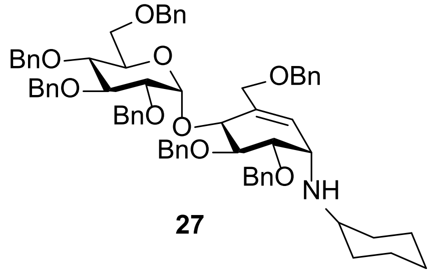

27

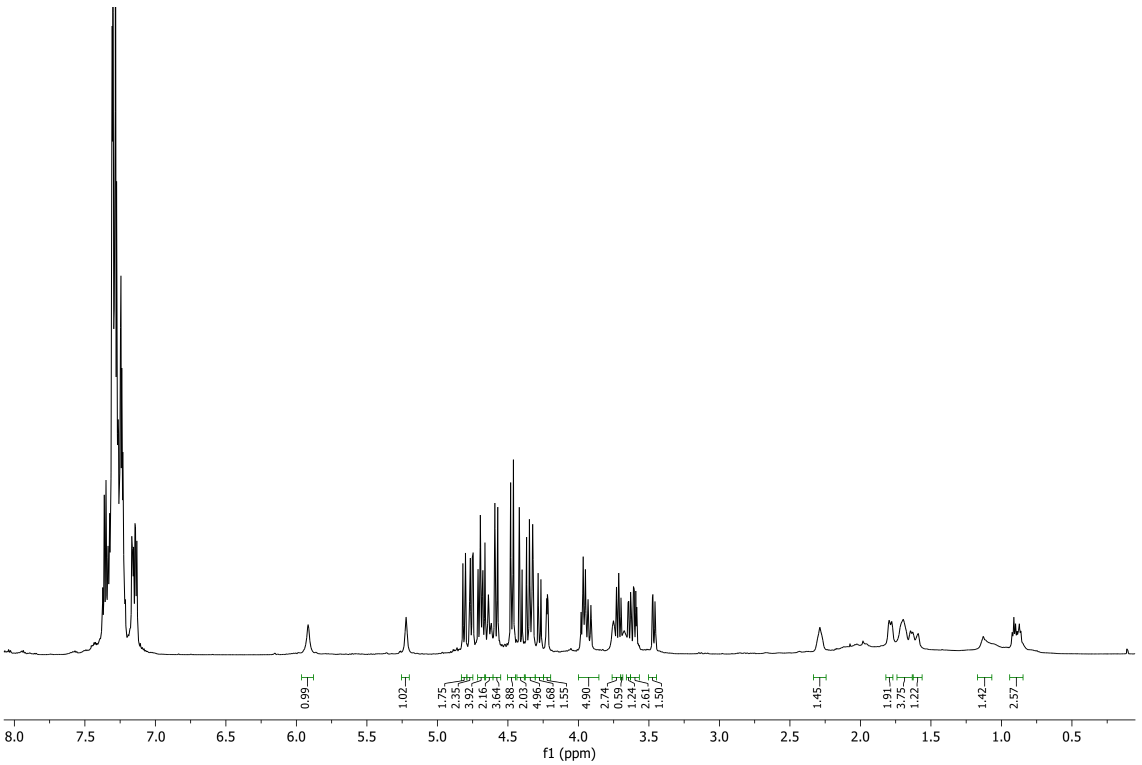

^{13}C NMR (1S,4R,5S,6S)-5,6-bis(benzyloxy)-3-((benzyloxy)methyl)-N-cyclohexyl-4-(((2S,3R,4S,5R,6R)-3,4,5-tris(benzyloxy)-6-((benzyloxy)methyl)tetrahydro-2H-pyran-2-yl)oxy)cyclohex-2-en-1-amine (**27**)

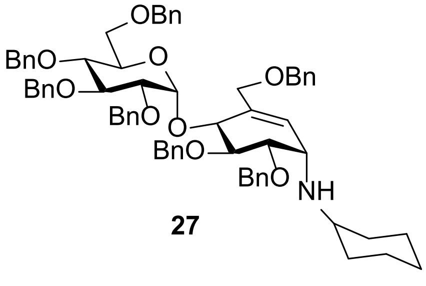

27

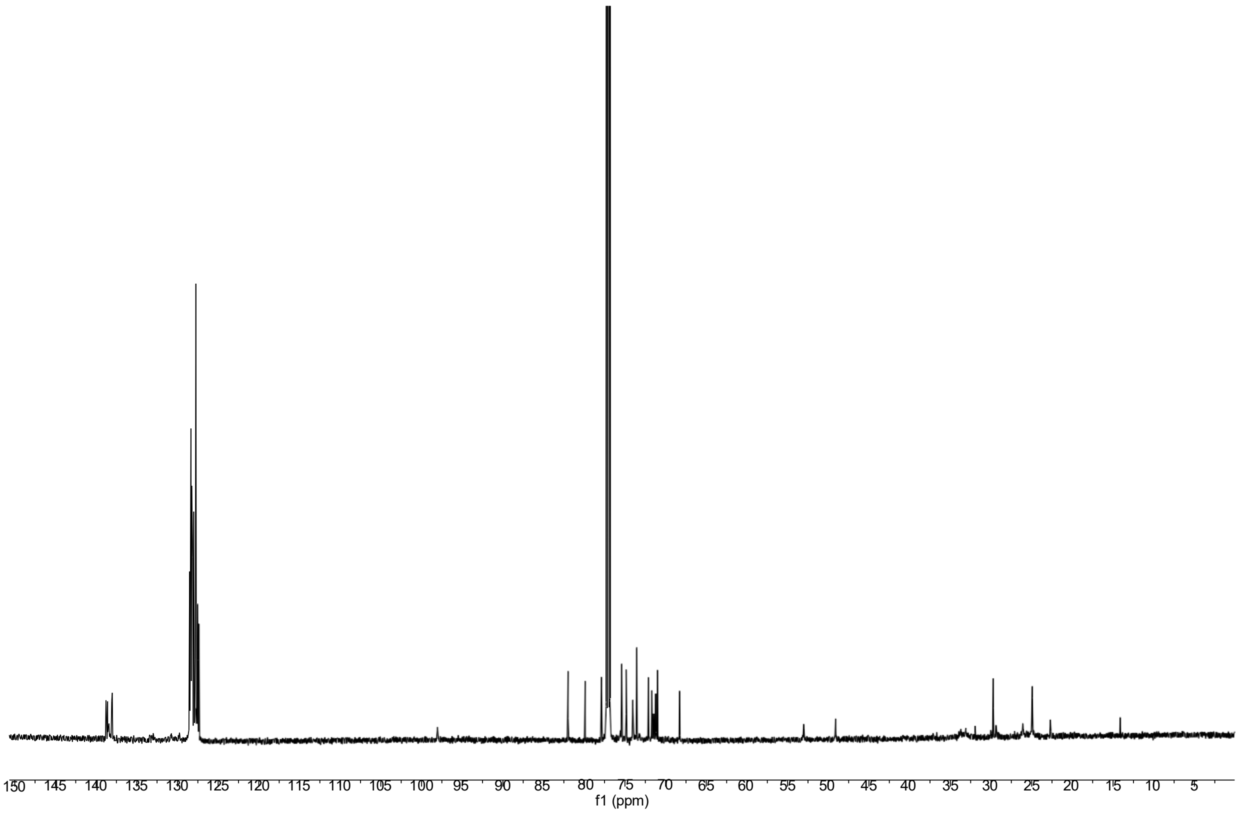

^{1}H NMR (2S,3R,4S,5S,6R)-2-(((1R,4S,5S,6R)-4-(cyclohexylamino)-5,6-dihydroxy-2-(hydroxymethyl)cyclohex-2-en-1-yl)oxy)-6-(hydroxymethyl)tetrahydro-2H-pyran-3,4,5-triol (**13**)

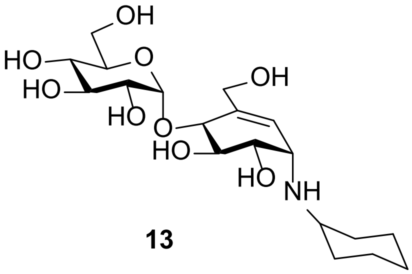

13

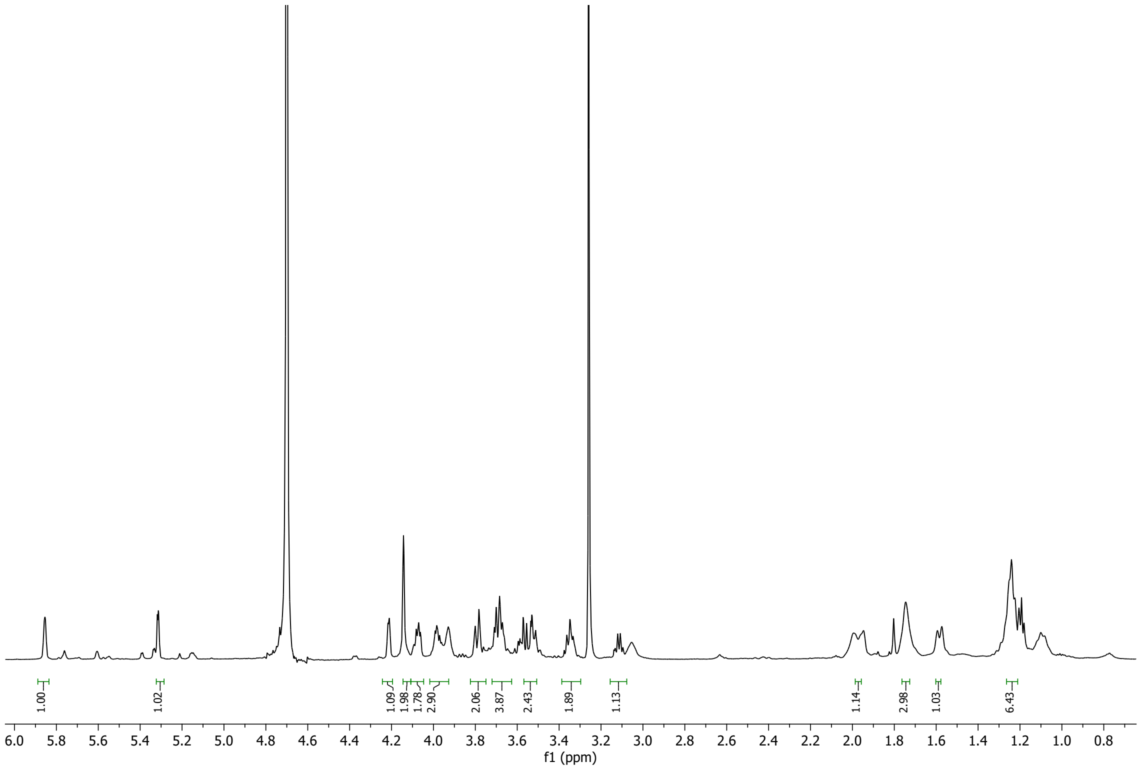

^{13}C NMR (2S,3R,4S,5S,6R)-2-(((1R,4S,5S,6R)-4-(cyclohexylamino)-5,6-dihydroxy-2-(hydroxymethyl)cyclohex-2-en-1-yl)oxy)-6-(hydroxymethyl)tetrahydro-2H-pyran-3,4,5-triol (**13**)

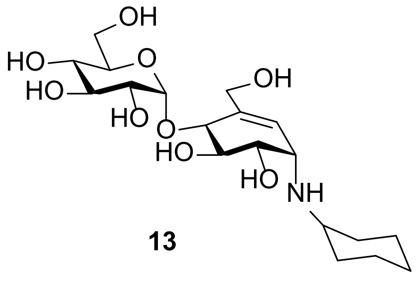

13

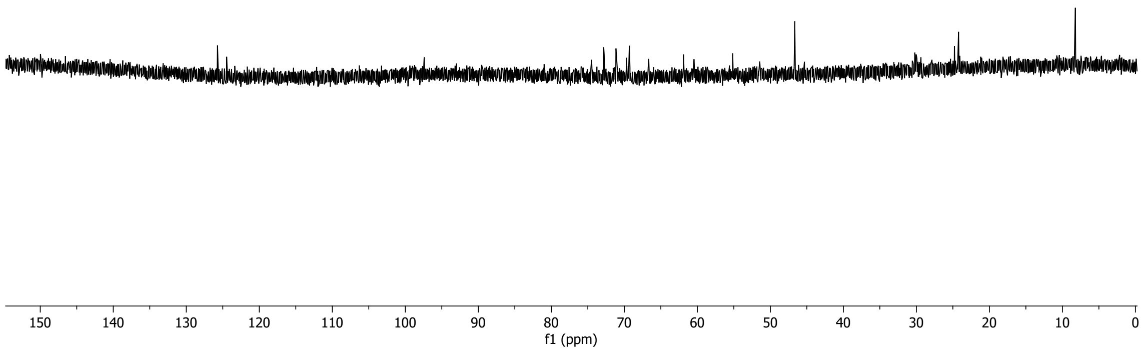

References

Si, A., Jayasinghe, T.D., Thanvi, R., Ronning, D.R., and Sucheck, S.J. (2021). Stereoselective synthesis of a 4-α-glucoside of valienamine and its X-ray structure in complex with Streptomyces coelicolor GlgE1-V279S. *Sci Rep* e13413.
